# Highly Pathogenic Avian Influenza A (H5N1) Virus in Swans, Central China, 2021

**DOI:** 10.1128/spectrum.02315-22

**Published:** 2022-09-14

**Authors:** Xianliang Ke, Zhongzi Yao, Yangyu Tang, Mengting Yang, Yong Li, Guoxiang Yang, Jing Chen, Guang Chen, Wei Feng, Hesong Zheng, Quanjiao Chen

**Affiliations:** a CAS Key Laboratory of Special Pathogens and Biosafety, Wuhan Institute of Virology, CAS Center for Influenza Research and Early Warning, Center for Biosafety Mega-Science, Chinese Academy of Sciences, Wuhan, China; b University of Chinese Academy of Sciences, Beijing, China; c The Monitoring Center of Wildlife Diseases and Resource of Hubei Province, Wuhan; d Wang Lake Wetland Nature Reserve Administration of Huangshi City, Huang shi, Hubei, China; Changchun Veterinary Research Institute

**Keywords:** H5N1, HPAIV, clade 2.3.4.4b, wild birds

## Abstract

Six highly pathogenic avian influenza (HPAI) H5N1 viruses (clade 2.3.4.4b) were detected in migratory birds in Hubei Province in November 2021. Phylogenetic analysis indicated that the viruses in the study included two different reassortants between H5N1 viruses that were circulating in Eurasia and low-pathogenic avian influenza viruses (LPAIVs). Several amino acid substitutions that contributed to the enhanced replication or virulence in mammals were observed in these viruses, suggesting a potential threat of the H5N1 viruses to human health.

**IMPORTANCE** Here, we obtained the whole-genomes of six H5N1 viruses from dead or rescued wild birds in Hubei Province. These viruses were divided into two genotypes and had different evolutionary trajectories from previously reported H5N1 viruses in China. Extensive reassortment events between high-pathogenic (HP) and low-pathogenic (LP) avian influenza viruses (AIVs) were observed in these viruses. Moreover, a key amino acid analysis also suggests a potential threat of H5N1 viruses to public health. Our work explored the prevalent patterns of H5N1 viruses in wild birds and replenished the viral population data of H5N1 viruses in central China.

## OBSERVATION

From when the highly pathogenic avian influenza (HPAI) H5N1 virus was first detected in chickens in Scotland in 1959 (1) to now, H5 HPAIVs have evolved into 10 clades (clades 0 to 9) (2). Reassortants of the H5 viruses circulate in wild birds and domestic poultry and have caused numerous outbreaks over the past 2 decades (3). In May 2020, a new H5 2.3.4.4b variant of the H5N8 virus was detected in Iraq (4), followed by outbreaks caused by H5Nx viruses in many Eurasian, Middle Eastern, and African countries, including the HPAI H5N1 viruses detected in Eurasian wigeons in the Netherlands on October 16, 2020 (4). The HA and M genes of these viruses are derived from “Iraq-like” isolates, and the remaining genes originate from the low-pathogenic avian influenza (LPAI) Eurasian avian lineage (4). After emerging in the Netherlands in 2020, H5N1 viruses from the 2.3.4.4b clade continue to circulate and spread worldwide (5–7). Beside outbreaks among bird species, human infection by HPAI H5N6 viruses bearing the clade 2.3.4.4b HA gene were reported in 2020 and 2021 in China (8, 9), raising serious concerns about the risk of HPAIVs to human health.

In November 2021, samples of 29 swans (Cygnus columbianus, 28 dead and one rescued) collected from the Longgan Lake National Nature Reserve Administration by the Monitoring Center of Wildlife Diseases and Resources of Hubei Province, China were sent to our laboratory. Samples included oropharyngeal and cloacal swabs as well as lung, liver, heart, etc. A total of eight samples were identified as H5N1 positive (Table S1). All eight viruses were sequenced using next-generation sequencing, and among them, the whole-genomes of six viruses were successfully obtained.

**FIG 1:**
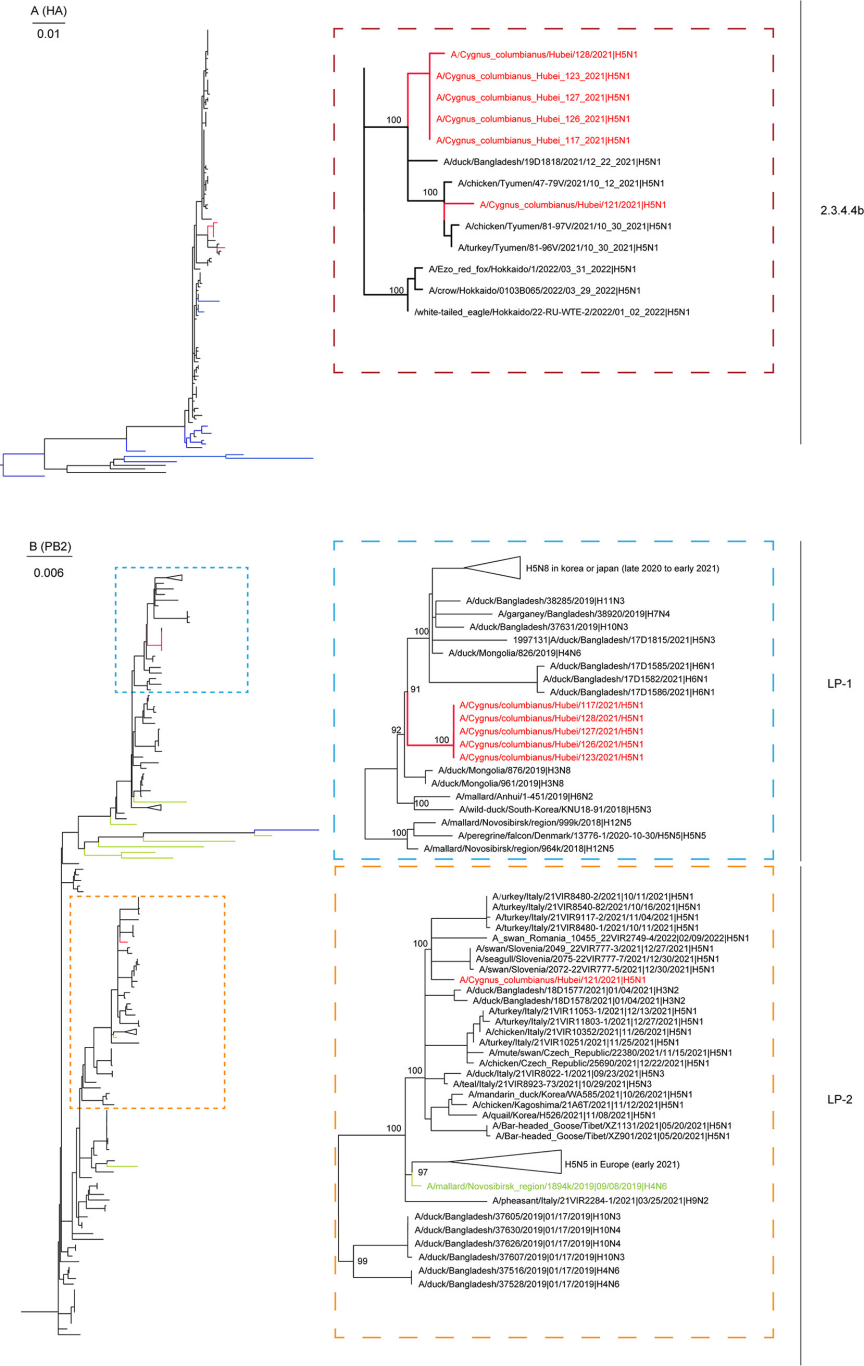
The maximum likelihood phylogenetic analyses of the HPAIV H5N1 in migratory birds in Hubei Province, Central China. Sequences identified in this study are marked in red, and sequences from human-origin H5 viruses are marked in blue. Sequences marked in green indicate genes of the donor-like strains from the study of Cui et al. (10). Larger versions of these phylogenetic trees are provided in Fig. S1. LP-1/2 means two different LPAIV gene pools. LP, low pathogenic.

**FIG 2:**
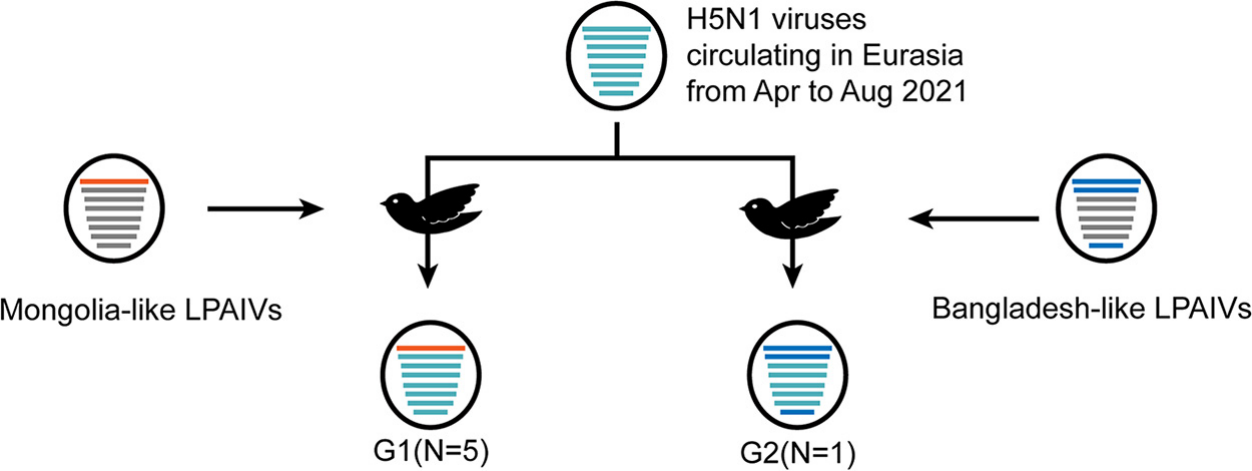
Hypothetical reassortment events of H5N1 viruses. The eight gene segments are indicated by horizontal bars within the ovals (from top to bottom: PB2, PB1, PA, HA, NP, NA, M, and NS). The colors of the bars indicate the different sources of the gene segments.

To reveal the genetic distribution of the six viruses in this study, maximum likelihood (ML) trees of all eight genes were constructed (Fig. 1; Fig. S1). According to the different gene distributions, the six viruses were divided into two genotypes: G1 and G2 (Fig. 2). The phylogenetic tree of HA showed that G1 and G2 clustered with those isolated from poultry in Russia and Bangladesh in 2021, and they were grouped into clade 2.3.4.4b (Fig. 1A).

The PB2 genes of G1 are clustered with low-pathogenic avian influenza viruses (LPAIVs) and shared the highest nucleotide identity with the H3N8 virus isolated from ducks in Mongolia in 2019 (99% to 99.1%). Except for PB2, the other genes of G1 were clustered together with those of H5Nx viruses circulating in Eurasia from 2021 to 2022 (Fig. S1).

The PB2 of G2, together with a number of H5Nx viruses circulating from late 2021 to early 2022, was closely related to the H3N2 AIVs isolated from Bangladesh, which indicated that the PB2 of G2 might ultimately be derived from LPAIVs (Fig. 1A).

The PB1 and NS genes of G2 gathered with those of duck viruses from Bangladesh, and they shared the highest nucleotide identity with those of A/duck/Bangladesh/18D1811/2022 (H5N3) (98.8%) and A/duck/Bangladesh/38557/2019 (H8N4) (98.3%), respectively. The PA/HA/NP/NA/M genes of G2 clustered with other H5Nx viruses circulating in Eurasia from 2021 to 2022, like those of G1 (Fig. S1). Recently, Cui et al. performed a phylogenic analysis of 233 representatives of globally circulating H5N1 strains and grouped these viruses into 16 genotypes (10). To analyze the phylogenetic relationship between the viruses in this study and those from the study of Cui et al. (10), we used the sequences of the donor strains from their research as the reference sequences for our genetic analysis (Fig. 1; Fig. S1). The PB1/PA/HA/NP/NA/M/NS genes of our G1 and the PA/HA/NP/NA/M genes of our G2 clustered with those of the Cui-G1, and the PB2 gene of our G2 clustered with those of the Cui-G5/7/12 (Fig. 1; Fig. S1). However, the PB2 gene of G1 and the PB1/NS genes of G2 showed different origins from the other 16 genotypes (Fig. S1), indicating that the H5N1 viruses in this study are novel reassortants.

To further explore the sources of these H5N1 viruses, we performed molecular dating analyses with maximum clade credibility (MCC) trees (Fig. S2; Table S2). The time to the most recent common ancestor (tMRCA) of the PB2 genes of G1 was dated to be July 2021 (95% highest posterior density from May 2021 to September 2021), and the tMRCA of the remaining seven genes of G1 were estimated to be between February and September 2021, indicating that the reassortment events might occur in a short term in mid-2021. The lack of sufficient viruses isolated from wild birds precludes the accurate dating of the reassortment event of the G2 virus, but the tMRCA of the PB2 and PB1 genes of the G1 virus with its closest donor strain, dated between January and May 2021, indicates that the most recent reassortment event likely occurred after January 2021.

The typical polybasic amino acid cleavage site (PLREKRRKR/G) of the HA protein indicated that all six strains were HPAIVs. The D94N/S123P/T160A mutations in the HA protein indicated that these viruses possibly have strong binding activity to α-2,6-linked SA receptors, which might increase their transmission ability in mammals (Table S3) (11–13). For PB2, PB1, and PA, several amino acid sites related to increased polymerase activity and enhanced pathogenicity in mammals were detected in all of the isolates (Table S3). Moreover, a few molecular markers that could cause increased virulence in mammals were detected in M1 and NS1 (Table S3).

## Discussion. 

Here, the whole-genomes of six HPAI H5N1 viruses were obtained. The HA genes of these viruses belong to clade 2.3.4.4b, and the viruses are identified as two novel reassortants (G1 and G2) between H5N1 viruses circulating in Eurasia and LPAIVs. G1 and G2 may be the products of a common precursor H5 virus that experienced different reassortment processes. After the reassortment, the G1 viruses obtained PB2 from LPAIVs, and the G2 virus obtained PB2/PB1/NS from LPAIVs. The molecular dating analysis showed that the precursor H5 virus circulated from February to September 2021, when migratory birds were resting and breeding in the breeding ground. Lakes and wetlands in Mongolia, as well as other high latitude areas, are ideal breeding grounds for birds. As indicated by the tMRCA analysis, the reassortment events between the precursor H5 virus and the LPAIVs circulating in the breeding ground, which generated the G1 viruses, happened in mid-2021. It is hard to trace back the complex reassortment process of the G2 virus. However, we noticed that the PB2 gene of the G2 virus, together with a number of H5Nx viruses circulating in Eurasia, formed a small monophylogenic subclade, the tMRCA of which is March 2019, which may indicate that the PB2 genes from the LPAIVs had circulated in H5Nx viruses in 2019.

The risk of human infection by the detected viruses was also evaluated. HA genes of the H5N1 viruses are closely related to those of the H5N6 human cases (Fig. S1) recently reported in China (8, 9), and they carried mutations that can increase their viral replication ability or virulence in mammals (13), suggesting a potential threat of the circulating H5N1 viruses to humans. Meanwhile, the H5 gene of two vaccine strains, A/whooper/swan/Shanxi4-1/2020 (H5N8) (14) and A/Astrakhan/3212/2020 (H5N8) (15), clustered with those of the detected H5N1 viruses, implicating their potential usage for zoonotic vaccination.

Longgan Lake Nature Reserve is an important stopover site along the East Asian-Australia flyway. Migratory birds travel between breeding and wintering grounds, share habitats with other bird flocks, and promote the transmission and reassortment of AIVs. So, we need to strengthen AIV surveillance in wild birds and poultry and take extra precautions to avoid potential threats to farm and human health caused by HPAIV H5N1.

### Data availability.

The whole-genome sequences of six H5N1 strains were submitted to GISAID (accession numbers: EPI2008854-861, EPI2008862-868, EPI2008870, EPI2009493-2009500, EPI2009516-2009523, EPI2009508-2009515, EPI2009524-2009531).
