# Supplementary material for: Highly Pathogenic Avian Influenza A (H5N1) Virus in Swans, Central China, 2021
Source: Microbiol Spectr. 2022 Sep 14;10(5):e02315-22. doi: 10.1128/spectrum.02315-22 (PMC9602517; doi:10.1128/spectrum.02315-22)
Supplement: Supplemental file 1 — Download spectrum.02315-22-s0001.pdf, PDF file, 2.5 MB [file spectrum.02315-22-s0001.pdf]

Supplementary Materials

Materials and Methods

Identification of avian influenza virus

RNA was extracted from oropharyngeal/cloacal swabs of rescued migratory birds or organs (lung, liver, kidney, spleen, and heart) of dead migrated birds in a Biosafety level-2 laboratory. A Nucleic Acid Extraction System with matched EX-RNA/DNA viral nucleic acid extraction kits (Tianlong Science and Technology, Co., Ltd.) was used for RNA extraction. One-step RT-PCR kits (Vazyme Biotech Co., Ltd. Nanjing, China) were used to detect the influenza A virus. Subtype identification was conducted using H5 and N1-specific primers as described previously (1).

Next generation sequencing (NGS)

The vRNA of H5N1 positive isolates were reverse transcribed to synthesize cDNA using MBT uni-12/13 primer (2). And then, NGS was used to obtain the eight gene sequences of viruses. Sequencing libraries were prepared and sequenced on MGISEQ-2000 (PE150) platform as described previously (3). Briefly, sequencing libraries were generated using an MGIEasy DNA Adapters-96 (Plate) kit (MGI Tech Co., Ltd, Shenzhen, China) and quantified using a Qubit 3.0 Fluorometer (Life Technologies, Grand Island, NY, USA) and an Agilent 4200 system (Agilent, Santa Clara, CA, USA). Sequencing depth for all isolates was 0.2G per sample.

Sequencing data assembly

The raw NGS reads were disposed by filtering out low-quality reads (10 bases with quality <10), adaptor-contaminated reads (with > 15 bp matched to the adapter sequence), poly-Ns (with eight Ns), duplication, and host contaminated reads (SOAP2 version 2.21; less than five mismatches)(4). The filtered reads were subjected to de novo assembly using Megahit (v1.2.9)(5), and the assembled short reads were mapped to the INFLUENZA database and the best-matched reference sequences were chosen. And then MAQ were used to perform reference-based assembly (6).

Phylogenetic analyses

All influenza A viruses from the GISAID and GenBank databases were downloaded to create a local influenza A virus database (updated to May 20, 2022). Blastn was performed locally with default parameters against the local influenza A virus database, using each segment of the six H5N1 viruses in the study as a query. Subsequently, the first 100 gene sequences in the output were collected and integrated into one dataset with the gene sequences in this study. Sequences of gene donor sequences in (7), HA gene of human H5N6 cases in China (8), and WHO-recommended vaccine strains (9) were added to the dataset to better understand the phylogenic relationship. Sequences in each dataset were aligned using MAFFT (v7.407) (10). After this, the gene coding sequence was selected to create

a new dataset for each of the six segments with duplicate sequences removed. Maximum likelihood trees were generated by IQ-tree software under the GTR-GAMMA model with 1000 bootstrap replicates(11).

The maximum clade credibility (MCC) trees of eight gene sequences were generated using BEAST (v1.10.4) (12)under the best substitution model, with an uncorrelated relaxed clock and chain length of 100,000,000. The best nucleotide substitution model of each tree was selected by the ModelFinder function of the IQ-tree according to the value of AIC (PB2, GTR+F+I+G4; PB1, GTR+F+I+G4; PA, GTR+F+I; HA, GTR+F+G4; NP, HKY+F+I; M, GTR+F+I; N1, GTR+F+G4; NS, GTR+F+G4.).

Reference

1. Chen Q, Wang H, Zhao L, Ma L, Wang R, Lei Y, et al. First documented case of avian influenza (H5N1) virus infection in a lion. Emerg Microbes Infect. 2016 Dec 21;5(12):e125.

2. Bi Y, Chen Q, Wang Q, Chen J, Jin T, Wong G, et al. Genesis, Evolution and Prevalence of H5N6 Avian Influenza Viruses in China. Cell Host & Microbe. 2016 Dec;20(6):810–21.

3. Xiong J, Zhou H, Fan L, Zhu G, Li Y, Chen G, et al. Emerging highly pathogenic avian influenza (H5N8) virus in migratory birds in Central China, 2020. Emerg Microbes Infect. 2021 Dec;10(1):1503–6.

4. Li R, Yu C, Li Y, Lam TW, Yiu SM, Kristiansen K, et al. SOAP2: an improved ultrafast tool for short read alignment. Bioinformatics. 2009 Aug 1;25(15):1966–7.

5. Li D, Luo R, Liu CM, Leung CM, Ting HF, Sadakane K, et al. MEGAHIT v1.0: A fast and scalable metagenome assembler driven by advanced methodologies and community practices. Methods. 2016 Jun 1;102:3–11.

6. Li H, Ruan J, Durbin R. Mapping short DNA sequencing reads and calling variants using mapping quality scores. Genome Res. 2008 Nov;18(11):1851–8.

7. Cui P, Shi J, Wang C, Zhang Y, Xing X, Kong H, et al. Global dissemination of H5N1 influenza viruses bearing the clade 2.3.4.4b HA gene and biologic analysis of the ones detected in China: Evolution of the globally circulating H5N1 viruses bearing the clade 2.3.4.4b HA. Emerging Microbes & Infections. 2022 Jun 14;1–41.

8. Gu W, Shi J, Cui P, Yan C, Zhang Y, Wang C, et al. Novel H5N6 reassortants bearing the clade 2.3.4.4b HA gene of H5N8 virus have been detected in poultry and caused multiple human infections in China. Emerging Microbes & Infections. 2022 Dec 31;11(1):1174–85.

9. World Health Organization. Antigenic and genetic characteristics of

zoonotic influenza A viruses and development of candidate vaccine viruses for pandemic preparedness.

10. Katoh K, Standley DM. MAFFT Multiple Sequence Alignment Software Version 7: Improvements in Performance and Usability. Molecular Biology and Evolution. 2013 Apr 1;30(4):772–80.

11. Nguyen LT, Schmidt HA, von Haeseler A, Minh BQ. IQ-TREE: A Fast and Effective Stochastic Algorithm for Estimating Maximum-Likelihood Phylogenies. Molecular Biology and Evolution. 2015 Jan;32(1):268–74.

12. Drummond AJ, Rambaut A. BEAST: Bayesian evolutionary analysis by sampling trees. BMC Evol Biol. 2007;7(1):214.

Fig S1. Phylogeny of eight gene segments of six H5N1 viruses. Maximum likelihood trees were generated by IQ-tree software under the GTR-GAMMA model with 1000 bootstrap replicates. Only CDS regions of gene sequences were used for the phylogenetic analyses. Sequences reported in this study are colored red. Sequences identified from human H5 cases are colored in blue. Sequences marked in green indicate genes of the donor-like strains in Cui's study. The black dot indicates H5 vaccine strains or candidate vaccine strains recommended by WHO.

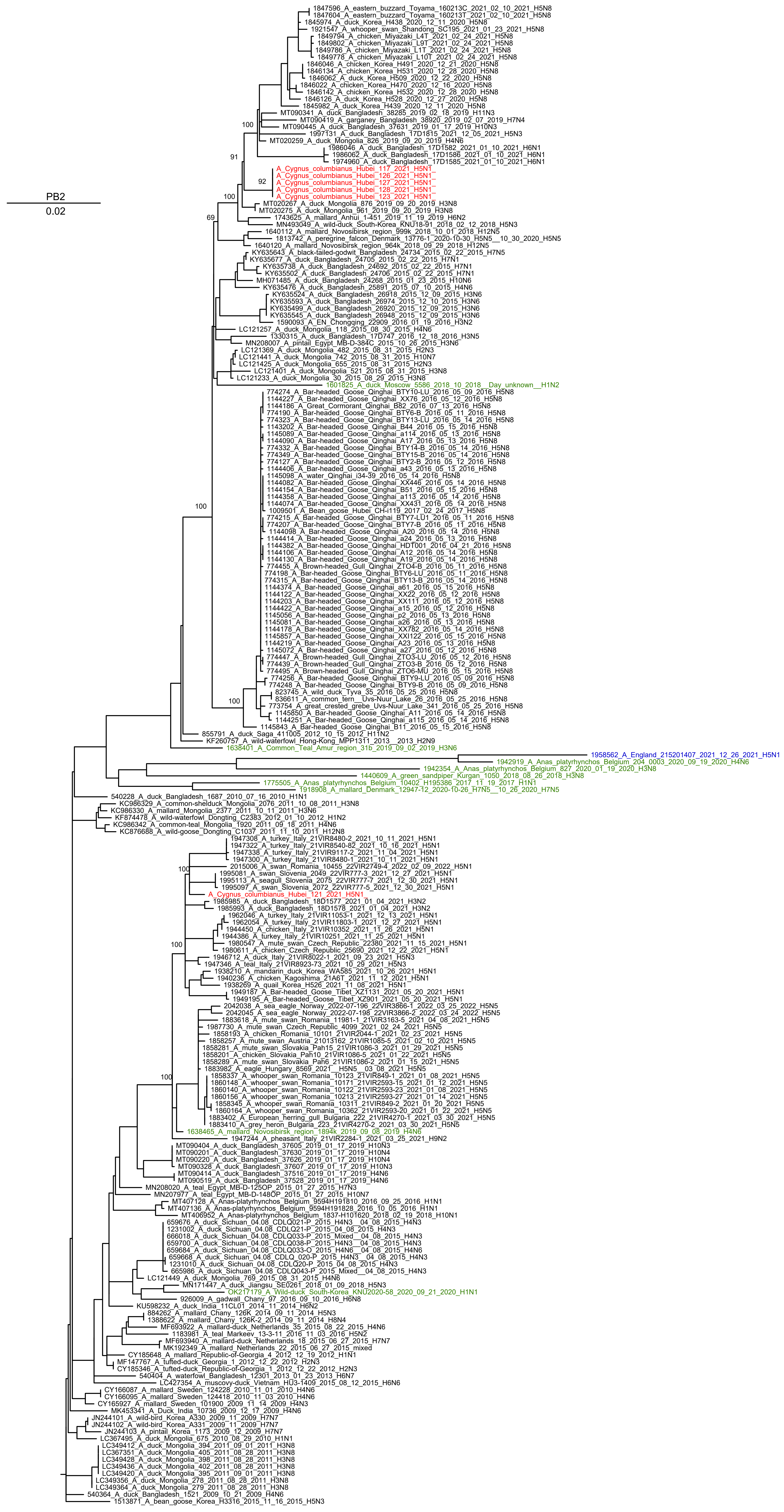
PB2
0.02
100
91
92
100
69
100
100
100
100
100

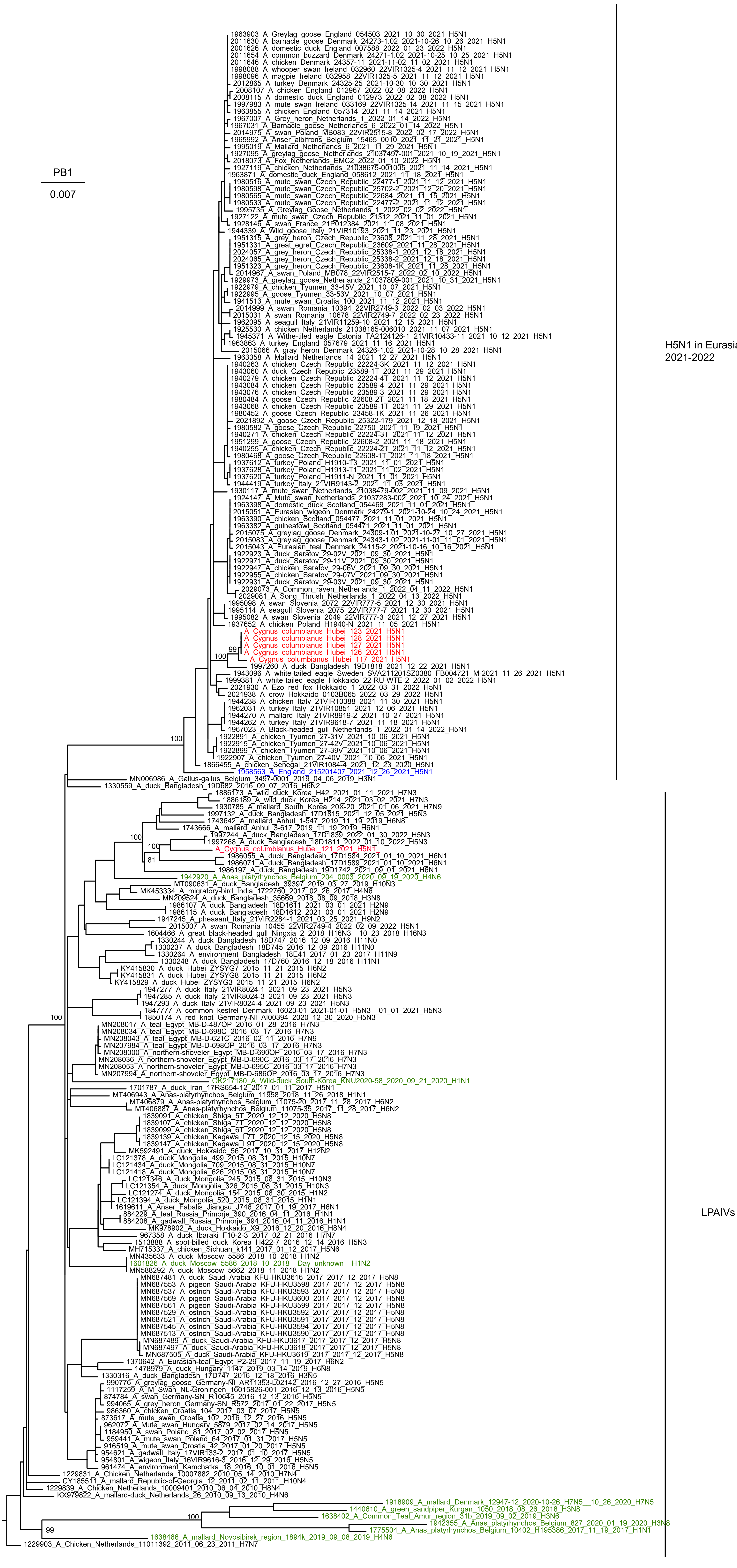

PB1
0.007
H5N1 in Eurasia 2021-2022
LPAIVs

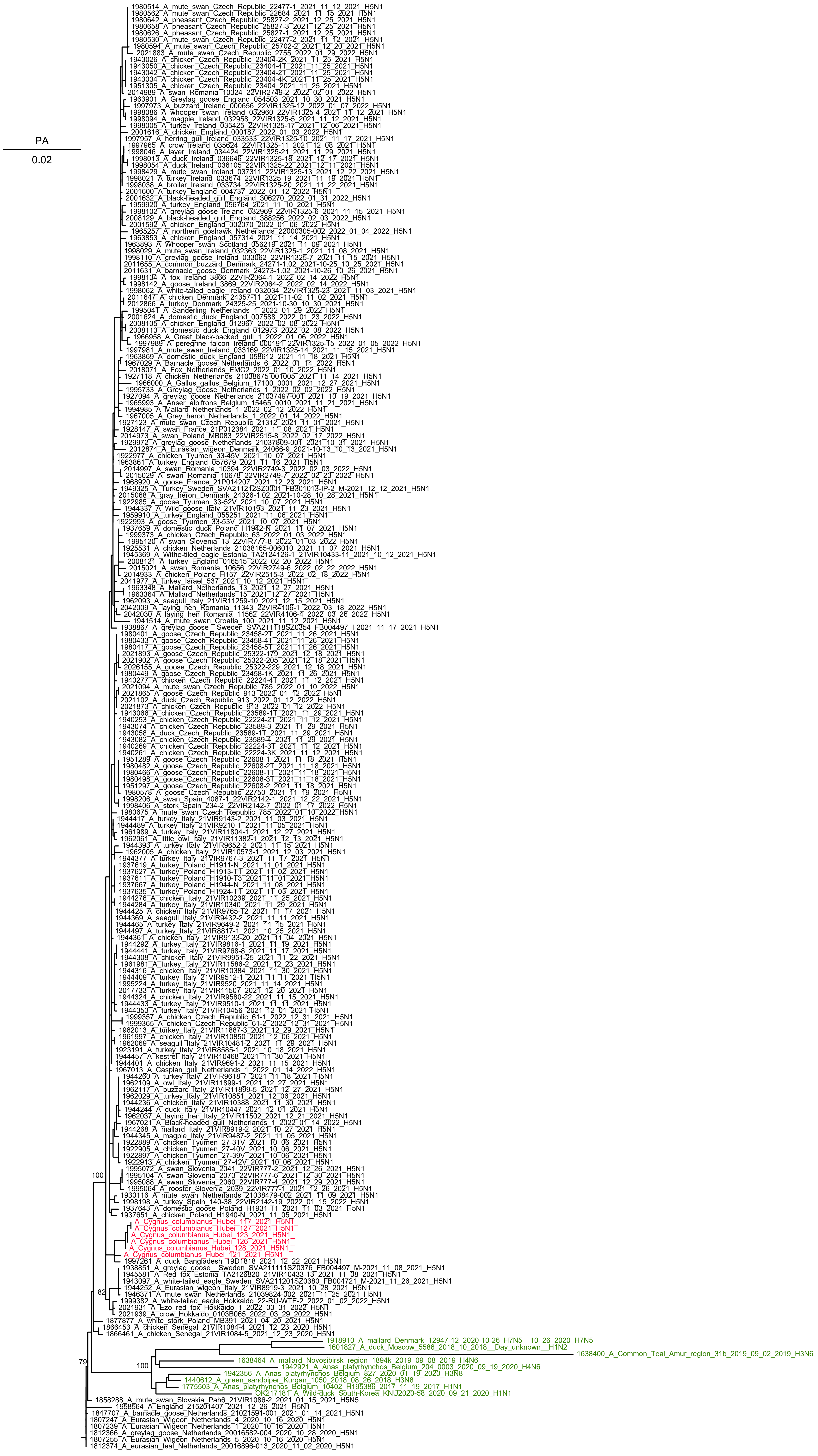

PA
0.02
1980514_A_mute_swan_Czech_Republic_22477-1_2021_11_12_2021_H5N1
1980562_A_mute_swan_Czech_Republic_22684_2021_11_15_2021_H5N1
1980642_A_pheasant_Czech_Republic_25827-2_2021_12_25_2021_H5N1
1980658_A_pheasant_Czech_Republic_25827-3_2021_12_25_2021_H5N1
1980626_A_pheasant_Czech_Republic_25827-1_2021_12_25_2021_H5N1
1980530_A_mute_swan_Czech_Republic_22477-2_2021_11_12_2021_H5N1
1980594_A_mute_swan_Czech_Republic_25702-2_2021_12_20_2021_H5N1
2021883_A_mute_swan_Czech_Republic_2755_2022_01_29_2022_H5N1
1943026_A_chicken_Czech_Republic_23404-2K_2021_11_25_2021_H5N1
1943050_A_chicken_Czech_Republic_23404-4T_2021_11_25_2021_H5N1
1943042_A_chicken_Czech_Republic_23404-2T_2021_11_25_2021_H5N1
1943034_A_chicken_Czech_Republic_23404-4K_2021_11_25_2021_H5N1
1951305_A_chicken_Czech_Republic_23404_2021_11_25_2021_H5N1
2014989_A_swan_Romania_10324_22VIR2749-2_2022_02_01_2022_H5N1
1963901_A_Greylag_goose_England_054503_2021_10_30_2021_H5N1
1997973_A_buzzard_Ireland_000656_22VIR1325-12_2022_01_07_2022_H5N1
1998086_A_whooper_swan_Ireland_032960_22VIR1325-4_2021_11_12_2021_H5N1
1998094_A_magpie_Ireland_032958_22VIR1325-5_2021_11_12_2021_H5N1
1998005_A_turkey_Ireland_035425_22VIR1325-17_2021_12_06_2021_H5N1
2001616_A_chicken_England_000187_2022_01_03_2022_H5N1
1997957_A_herring_gull_Ireland_033533_22VIR1325-10_2021_11_17_2021_H5N1
1997965_A_crow_Ireland_035624_22VIR1325-11_2021_12_08_2021_H5N1
1998046_A_layer_Ireland_034424_22VIR1325-21_2021_11_29_2021_H5N1
1998013_A_duck_Ireland_036646_22VIR1325-18_2021_12_17_2021_H5N1
1998054_A_duck_Ireland_036105_22VIR1325-22_2021_12_11_2021_H5N1
1998429_A_mute_swan_Ireland_037311_22VIR1325-13_2021_12_22_2021_H5N1
1998021_A_turkey_Ireland_033674_22VIR1325-19_2021_11_19_2021_H5N1
1998038_A_broiler_Ireland_033734_22VIR1325-20_2021_11_22_2021_H5N1
2001600_A_turkey_England_004737_2022_01_12_2022_H5N1
2001632_A_black-headed_gull_England_306270_2022_01_31_2022_H5N1
1959920_A_turkey_England_056764_2021_11_10_2021_H5N1
1998102_A_greylag_goose_Ireland_032969_22VIR1325-6_2021_11_15_2021_H5N1
2008129_A_black-headed_gull_England_388256_2022_02_03_2022_H5N1
2001592_A_chicken_England_002070_2022_01_06_2022_H5N1
1965257_A_northern_goshawk_Netherlands_22000305-002_2022_01_04_2022_H5N1
1963853_A_chicken_England_057314_2021_11_14_2021_H5N1
1963893_A_Whooper_swan_Scotland_056219_2021_11_09_2021_H5N1
1998029_A_mute_swan_Ireland_032363_22VIR1325-1_2021_11_08_2021_H5N1
1998110_A_greylag_goose_Ireland_033062_22VIR1325-7_2021_11_15_2021_H5N1
2011655_A_common_buzzard_Denmark_24271-1.02_2021-10-25_10_25_2021_H5N1
2011631_A_barnacle_goose_Denmark_24273-1.02_2021-10-26_10_26_2021_H5N1
1998134_A_fox_Ireland_3866_22VIR2064-1_2022_02_14_2022_H5N1
1998142_A_goose_Ireland_3869_22VIR2064-2_2022_02_14_2022_H5N1
1998062_A_white-tailed_eagle_Ireland_032034_22VIR1325-23_2021_11_03_2021_H5N1
2011647_A_chicken_Denmark_24357-11_2021-11-02_11_02_2021_H5N1
2012866_A_turkey_Denmark_24325-25_2021-10-30_10_30_2021_H5N1
1995041_A_Sanderling_Netherlands_1_2022_01_29_2022_H5N1
2001624_A_domestic_duck_England_007588_2022_01_23_2022_H5N1
2008105_A_chicken_England_012967_2022_02_08_2022_H5N1
2008113_A_domestic_duck_England_012973_2022_02_08_2022_H5N1
1966958_A_Great_black-backed_gull_1_2022_01_06_2022_H5N1
1997989_A_peregrine_falcon_Ireland_000191_22VIR1325-15_2022_01_05_2022_H5N1
1997981_A_mute_swan_Ireland_033169_22VIR1325-14_2021_11_15_2021_H5N1
1963869_A_domestic_duck_England_058612_2021_11_18_2021_H5N1
1967029_A_Barnacle_goose_Netherlands_6_2022_01_14_2022_H5N1
2018071_A_Fox_Netherlands_EMC2_2022_01_10_2022_H5N1
1927118_A_chicken_Netherlands_21038675-001005_2021_11_14_2021_H5N1
1966000_A_Gallus_gallus_Belgium_17100_0001_2021_12_27_2021_H5N1
1995733_A_Greylag_Goose_Netherlands_1_2022_02_02_2022_H5N1
1927094_A_greylag_goose_Netherlands_21037497-001_2021_10_19_2021_H5N1
1965993_A_Anser_albifrons_Belgium_15465_0010_2021_11_21_2021_H5N1
1994985_A_Mallard_Netherlands_1_2022_02_12_2022_H5N1
1967005_A_Grey_heron_Netherlands_1_2022_01_14_2022_H5N1
1927123_A_mute_swan_Czech_Republic_21312_2021_11_01_2021_H5N1
1928147_A_swan_France_21P012384_2021_11_08_2021_H5N1
2014973_A_swan_Poland_MB083_22VIR2515-8_2022_02_17_2022_H5N1
1929972_A_greylag_goose_Netherlands_21037809-001_2021_10_31_2021_H5N1
2012874_A_Eurasian_wigeon_Denmark_24066-9_2021-10-13_10_13_2021_H5N1
1922977_A_chicken_Tyumen_33-45V_2021_10_07_2021_H5N1
1963861_A_turkey_England_057679_2021_11_16_2021_H5N1
2014997_A_swan_Romania_10394_22VIR2749-3_2022_02_03_2022_H5N1
2015029_A_swan_Romania_10678_22VIR2749-7_2022_02_23_2022_H5N1
1968920_A_goose_France_21P014207_2021_12_23_2021_H5N1
1949325_A_Turkey_Sweden_SVA211212SZ0001_FB301013-IP-2_M-2021_12_12_2021_H5N1
2015068_A_gray_heron_Denmark_24326-1.02_2021-10-28_10_28_2021_H5N1
1922985_A_goose_Tyumen_33-52V_2021_10_07_2021_H5N1
1944337_A_Wild_goose_Italy_21VIR10193_2021_11_23_2021_H5N1
1959910_A_turkey_England_055251_2021_11_06_2021_H5N1
1922993_A_goose_Tyumen_33-53V_2021_10_07_2021_H5N1
1937659_A_domestic_duck_Poland_H1942-N_2021_11_07_2021_H5N1
1999373_A_chicken_Czech_Republic_63_2022_01_03_2022_H5N1
1995120_A_swan_Slovenia_13_22VIR777-8_2022_01_03_2022_H5N1
1925531_A_chicken_Netherlands_21038165-006010_2021_11_07_2021_H5N1
1945369_A_Withe-tiled_eagle_Estonia_TA2124126-1_21VIR10433-11_2021_10_12_2021_H5N1
2008121_A_turkey_England_016515_2022_02_20_2022_H5N1
2015021_A_swan_Romania_10656_22VIR2749-6_2022_02_22_2022_H5N1
2014933_A_chicken_Poland_H157_22VIR2515-3_2022_02_18_2022_H5N1
2041977_A_turkey_Israel_537_2021_10_12_2021_H5N1
1963348_A_Mallard_Netherlands_13_2021_12_27_2021_H5N1
1963364_A_Mallard_Netherlands_15_2021_12_27_2021_H5N1
1962093_A_seagull_Italy_21VIR11259-10_2021_12_15_2021_H5N1
2042009_A_laying_hen_Romania_11343_22VIR4106-1_2022_03_18_2022_H5N1
2042030_A_laying_hen_Romania_11562_22VIR4106-4_2022_03_26_2022_H5N1
1941514_A_mute_swan_Croatia_100_2021_11_12_2021_H5N1
1938867_A_greylag_goose__Sweden_SVA211118SZ0354_FB004497_I-2021_11_17_2021_H5N1
1980401_A_goose_Czech_Republic_23458-2T_2021_11_26_2021_H5N1
1980433_A_goose_Czech_Republic_23458-4T_2021_11_26_2021_H5N1
1980417_A_goose_Czech_Republic_23458-5T_2021_11_26_2021_H5N1
2021893_A_goose_Czech_Republic_25322-179_2021_12_18_2021_H5N1
2021902_A_goose_Czech_Republic_25322-205_2021_12_18_2021_H5N1
2026155_A_goose_Czech_Republic_25322-229_2021_12_18_2021_H5N1
1980449_A_goose_Czech_Republic_23458-1K_2021_11_26_2021_H5N1
1940277_A_chicken_Czech_Republic_22224-4T_2021_11_12_2021_H5N1
2021094_A_mute_swan_Czech_Republic_785_2022_01_10_2022_H5N1
2021865_A_goose_Czech_Republic_913_2022_01_12_2022_H5N1
2021102_A_duck_Czech_Republic_913_2022_01_12_2022_H5N1
2021873_A_chicken_Czech_Republic_913_2022_01_12_2022_H5N1
1943066_A_chicken_Czech_Republic_23589-1T_2021_11_29_2021_H5N1
1940253_A_chicken_Czech_Republic_22224-2T_2021_11_12_2021_H5N1
1943074_A_chicken_Czech_Republic_23589-3_2021_11_29_2021_H5N1
1943058_A_duck_Czech_Republic_23589-1T_2021_11_29_2021_H5N1
1943082_A_chicken_Czech_Republic_23589-4_2021_11_29_2021_H5N1
1940269_A_chicken_Czech_Republic_22224-3T_2021_11_12_2021_H5N1
1940261_A_chicken_Czech_Republic_22224-3K_2021_11_12_2021_H5N1
1951289_A_goose_Czech_Republic_22608-1_2021_11_18_2021_H5N1
1980482_A_goose_Czech_Republic_22608-2T_2021_11_18_2021_H5N1
1980466_A_goose_Czech_Republic_22608-1T_2021_11_18_2021_H5N1
1980498_A_goose_Czech_Republic_22608-3T_2021_11_18_2021_H5N1
1951297_A_goose_Czech_Republic_22608-2_2021_11_18_2021_H5N1
1980578_A_goose_Czech_Republic_22750_2021_11_19_2021_H5N1
1998206_A_swan_Spain_4087-1_22VIR2142-1_2021_12_22_2021_H5N1
1998406_A_stork_Spain_234-2_22VIR2142-7_2022_01_17_2022_H5N1
1980675_A_mute_swan_Czech_Republic_785_2022_01_10_2022_H5N1
1944417_A_turkey_Italy_21VIR9143-2_2021_11_03_2021_H5N1
1944489_A_turkey_Italy_21VIR9210-1_2021_11_05_2021_H5N1
1961989_A_turkey_Italy_21VIR11804-1_2021_12_27_2021_H5N1
1962061_A_little_owl_Italy_21VIR11382-1_2021_12_13_2021_H5N1
1944393_A_turkey_Italy_21VIR9652-2_2021_11_15_2021_H5N1
1962005_A_chicken_Italy_21VIR10573-1_2021_12_03_2021_H5N1
1944377_A_turkey_Italy_21VIR9767-3_2021_11_17_2021_H5N1
1937619_A_turkey_Poland_H1911-N_2021_11_01_2021_H5N1
1937627_A_turkey_Poland_H1913-T1_2021_11_02_2021_H5N1
1937611_A_turkey_Poland_H1910-T3_2021_11_01_2021_H5N1
1937667_A_turkey_Poland_H1944-N_2021_11_08_2021_H5N1
1937635_A_turkey_Poland_H1924-T1_2021_11_03_2021_H5N1
1944276_A_chicken_Italy_21VIR10239_2021_11_25_2021_H5N1
1944284_A_turkey_Italy_21VIR10340_2021_11_29_2021_H5N1
1944425_A_chicken_Italy_21VIR9765-12_2021_11_17_2021_H5N1
1944369_A_seagull_Italy_21VIR9432-2_2021_11_11_2021_H5N1
1944465_A_turkey_Italy_21VIR9649-2_2021_11_15_2021_H5N1
1944497_A_turkey_Italy_21VIR8817-1_2021_10_25_2021_H5N1
1944361_A_chicken_Italy_21VIR9133-20_2021_11_04_2021_H5N1
1944292_A_turkey_Italy_21VIR9816-1_2021_11_19_2021_H5N1
1944441_A_turkey_Italy_21VIR9768-8_2021_11_17_2021_H5N1
1944308_A_chicken_Italy_21VIR9951-25_2021_11_22_2021_H5N1
1961981_A_turkey_Italy_21VIR11586-2_2021_12_23_2021_H5N1
1944316_A_chicken_Italy_21VIR10384_2021_11_30_2021_H5N1
1944409_A_turkey_Italy_21VIR9512-1_2021_11_11_2021_H5N1
1995224_A_turkey_Italy_21VIR9520_2021_11_14_2021_H5N1
2017733_A_turkey_Italy_21VIR11507_2021_12_20_2021_H5N1
1944324_A_chicken_Italy_21VIR9580-22_2021_11_15_2021_H5N1
1944433_A_turkey_Italy_21VIR9510-1_2021_11_11_2021_H5N1
1944353_A_turkey_Italy_21VIR10456_2021_12_01_2021_H5N1
1999357_A_chicken_Czech_Republic_61-1_2022_12_31_2021_H5N1
1999365_A_chicken_Czech_Republic_61-2_2022_12_31_2021_H5N1
1962013_A_turkey_Italy_21VIR11887-3_2021_12_29_2021_H5N1
1961997_A_chicken_Italy_21VIR10850_2021_12_06_2021_H5N1
1962069_A_seagull_Italy_21VIR10481-2_2021_11_29_2021_H5N1
1923191_A_turkey_Italy_21VIR8585-1_2021_10_18_2021_H5N1
1944457_A_kestrel_Italy_21VIR10468_2021_11_30_2021_H5N1
1944401_A_chicken_Italy_21VIR9691-2_2021_11_15_2021_H5N1
1967013_A_Caspian_gull_Netherlands_1_2022_01_14_2022_H5N1
1944260_A_turkey_Italy_21VIR9618-7_2021_11_18_2021_H5N1
1962109_A_owl_Italy_21VIR11899-1_2021_12_27_2021_H5N1
1962117_A_buzzard_Italy_21VIR11899-5_2021_12_27_2021_H5N1
1962029_A_turkey_Italy_21VIR10851_2021_12_06_2021_H5N1
1944236_A_chicken_Italy_21VIR10388_2021_11_30_2021_H5N1
1944244_A_duck_Italy_21VIR10447_2021_12_01_2021_H5N1
1962037_A_laying_hen_Italy_21VIR11502_2021_12_21_2021_H5N1
1967021_A_Black-headed_gull_Netherlands_1_2022_01_14_2022_H5N1
1944268_A_mallard_Italy_21VIR8919-2_2021_10_27_2021_H5N1
1944345_A_magpie_Italy_21VIR9487-2_2021_11_05_2021_H5N1
1922889_A_chicken_Tyumen_27-31V_2021_10_06_2021_H5N1
1922905_A_chicken_Tyumen_27-40V_2021_10_06_2021_H5N1
1922897_A_chicken_Tyumen_27-39V_2021_10_06_2021_H5N1
1922913_A_chicken_Tyumen_27-42V_2021_10_06_2021_H5N1
100
1995072_A_swan_Slovenia_2041_22VIR777-2_2021_12_26_2021_H5N1
1995104_A_swan_Slovenia_2073_22VIR777-6_2021_12_30_2021_H5N1
1995088_A_swan_Slovenia_2060_22VIR777-4_2021_12_29_2021_H5N1
1995064_A_rooster_Slovenia_2039_22VIR777-1_2021_12_26_2021_H5N1
1930116_A_mute_swan_Netherlands_21038479-002_2021_11_09_2021_H5N1
1998198_A_turkey_Spain_140-38_22VIR2142-19_2022_01_15_2022_H5N1
1937643_A_domestic_goose_Poland_H1931-T1_2021_11_03_2021_H5N1
1937651_A_chicken_Poland_H1940-N_2021_11_05_2021_H5N1
A_Cygnus_columbianus_Hubei_117_2021_H5N1_
A_Cygnus_columbianus_Hubei_127_2021_H5N1_
A_Cygnus_columbianus_Hubei_123_2021_H5N1_
A_Cygnus_columbianus_Hubei_126_2021_H5N1_
A_Cygnus_columbianus_Hubei_128_2021_H5N1_
A_Cygnus_columbianus_Hubei_121_2021_H5N1_
1997261_A_duck_Bangladesh_19D1818_2021_12_22_2021_H5N1
1938851_A_greylag_goose__Sweden_SVA211111SZ0376_FB004497_M-2021_11_08_2021_H5N1
1945581_A_Red_fox_Estonia_TA2126820_21VIR10433-13_2021_11_08_2021_H5N1
1943097_A_white-tailed_eagle_Sweden_SVA211201SZ0380_FB004721_M-2021_11_26_2021_H5N1
1944252_A_Eurasian_wigeon_Italy_21VIR8919-3_2021_10_28_2021_H5N1
82
1946371_A_mute_swan_Netherlands_21039824-002_2021_11_25_2021_H5N1
1999382_A_white-tailed_eagle_Hokkaido_22-RU-WTE-2_2022_01_02_2022_H5N1
2021931_A_Ezo_red_fox_Hokkaido_1_2022_03_31_2022_H5N1
2021939_A_crow_Hokkaido_0103B065_2022_03_29_2022_H5N1
1877877_A_white_stork_Poland_MB391_2021_04_20_2021_H5N1
1866453_A_chicken_Senegal_21VIR1084-4_2021_12_23_2020_H5N1
1866461_A_chicken_Senegal_21VIR1084-5_2021_12_23_2020_H5N1
1918910_A_mallard_Denmark_12947-12_2020-10-26_H7N5__10_26_2020_H7N5
1601827_A_duck_Moscow_5586_2018_10_2018__Day_unknown__H1N2
1638400_A_Common_Teal_Amur_region_31b_2019_09_02_2019_H3N6
1638464_A_mallard_Novosibirsk_region_1894k_2019_09_08_2019_H4N6
79
100
1942921_A_Anas_platyrhynchos_Belgium_204_0003_2020_09_19_2020_H4N6
1942356_A_Anas_platyrhynchos_Belgium_827_2020_01_19_2020_H3N8
1440612_A_green_sandpiper_Kurgan_1050_2018_08_26_2018_H3N8
1775503_A_Anas_platyrhynchos_Belgium_10402_H195386_2017_11_19_2017_H1N1
OK217181_A_Wild-duck_South-Korea_KNU2020-58_2020_09_21_2020_H1N1
1858288_A_mute_swan_Slovakia_Pah6_21VIR1086-2_2021_01_15_2021_H5N5
1958564_A_England_215201407_2021_12_26_2021_H5N1
1847707_A_barnacle_goose_Netherlands_21021591-001_2021_01_14_2021_H5N1
1807247_A_Eurasian_Wigeon_Netherlands_4_2020_10_16_2020_H5N1
1807239_A_Eurasian_Wigeon_Netherlands_1_2020_10_16_2020_H5N1
1812366_A_greylag_goose_Netherlands_20016582-004_2020_10_28_2020_H5N1
1807255_A_Eurasian_Wigeon_Netherlands_5_2020_10_16_2020_H5N1
1812374_A_eurasian_teal_Netherlands_20016896-013_2020_11_02_2020_H5N1

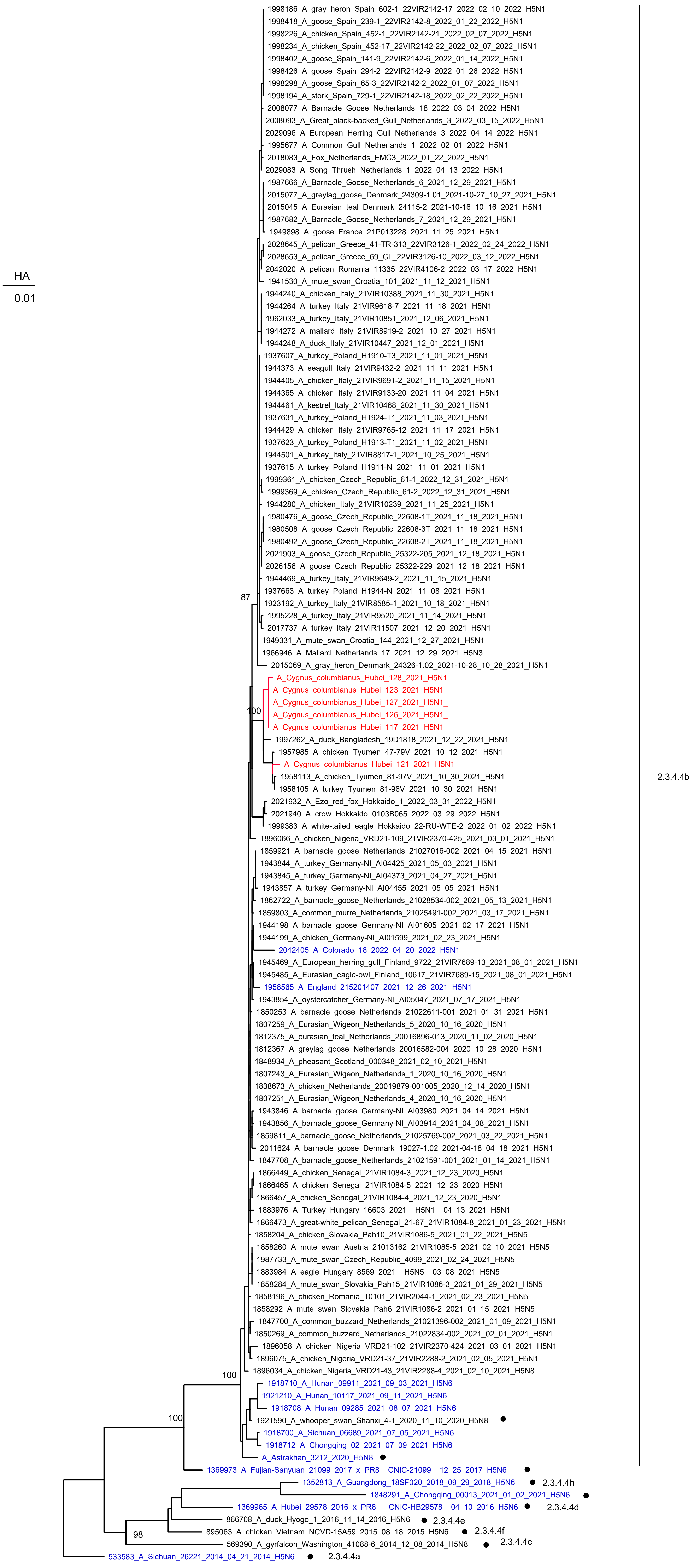
HA
0.01
1998186_A_gray_heron_Spain_602-1_22VIR2142-17_2022_02_10_2022_H5N1
1998418_A_goose_Spain_239-1_22VIR2142-8_2022_01_22_2022_H5N1
1998226_A_chicken_Spain_452-1_22VIR2142-21_2022_02_07_2022_H5N1
1998234_A_chicken_Spain_452-17_22VIR2142-22_2022_02_07_2022_H5N1
1998402_A_goose_Spain_141-9_22VIR2142-6_2022_01_14_2022_H5N1
1998426_A_goose_Spain_294-2_22VIR2142-9_2022_01_26_2022_H5N1
1998298_A_goose_Spain_65-3_22VIR2142-2_2022_01_07_2022_H5N1
1998194_A_stork_Spain_729-1_22VIR2142-18_2022_02_22_2022_H5N1
2008077_A_Barnacle_Goose_Netherlands_18_2022_03_04_2022_H5N1
2008093_A_Great_black-backed_Gull_Netherlands_3_2022_03_15_2022_H5N1
2029096_A_European_Herring_Gull_Netherlands_3_2022_04_14_2022_H5N1
1995677_A_Common_Gull_Netherlands_1_2022_02_01_2022_H5N1
2018083_A_Fox_Netherlands_EMC3_2022_01_22_2022_H5N1
2029083_A_Song_Thrush_Netherlands_1_2022_04_13_2022_H5N1
1987666_A_Barnacle_Goose_Netherlands_6_2021_12_29_2021_H5N1
2015077_A_greylag_goose_Denmark_24309-1.01_2021-10-27_10_27_2021_H5N1
2015045_A_Eurasian_teal_Denmark_24115-2_2021-10-16_10_16_2021_H5N1
1987682_A_Barnacle_Goose_Netherlands_7_2021_12_29_2021_H5N1
1949898_A_goose_France_21P013228_2021_11_25_2021_H5N1
2028645_A_pelican_Greece_41-TR-313_22VIR3126-1_2022_02_24_2022_H5N1
2028653_A_pelican_Greece_69_CL_22VIR3126-10_2022_03_12_2022_H5N1
2042020_A_pelican_Romania_11335_22VIR4106-2_2022_03_17_2022_H5N1
1941530_A_mute_swan_Croatia_101_2021_11_12_2021_H5N1
1944240_A_chicken_Italy_21VIR10388_2021_11_30_2021_H5N1
1944264_A_turkey_Italy_21VIR9618-7_2021_11_18_2021_H5N1
1962033_A_turkey_Italy_21VIR10851_2021_12_06_2021_H5N1
1944272_A_mallard_Italy_21VIR8919-2_2021_10_27_2021_H5N1
1944248_A_duck_Italy_21VIR10447_2021_12_01_2021_H5N1
1937607_A_turkey_Poland_H1910-T3_2021_11_01_2021_H5N1
1944373_A_seagull_Italy_21VIR9432-2_2021_11_11_2021_H5N1
1944405_A_chicken_Italy_21VIR9691-2_2021_11_15_2021_H5N1
1944365_A_chicken_Italy_21VIR9133-20_2021_11_04_2021_H5N1
1944461_A_kestrel_Italy_21VIR10468_2021_11_30_2021_H5N1
1937631_A_turkey_Poland_H1924-T1_2021_11_03_2021_H5N1
1944429_A_chicken_Italy_21VIR9765-12_2021_11_17_2021_H5N1
1937623_A_turkey_Poland_H1913-T1_2021_11_02_2021_H5N1
1944501_A_turkey_Italy_21VIR8817-1_2021_10_25_2021_H5N1
1937615_A_turkey_Poland_H1911-N_2021_11_01_2021_H5N1
1999361_A_chicken_Czech_Republic_61-1_2022_12_31_2021_H5N1
1999369_A_chicken_Czech_Republic_61-2_2022_12_31_2021_H5N1
1944280_A_chicken_Italy_21VIR10239_2021_11_25_2021_H5N1
1980476_A_goose_Czech_Republic_22608-1T_2021_11_18_2021_H5N1
1980508_A_goose_Czech_Republic_22608-3T_2021_11_18_2021_H5N1
1980492_A_goose_Czech_Republic_22608-2T_2021_11_18_2021_H5N1
2021903_A_goose_Czech_Republic_25322-205_2021_12_18_2021_H5N1
2026156_A_goose_Czech_Republic_25322-229_2021_12_18_2021_H5N1
1944469_A_turkey_Italy_21VIR9649-2_2021_11_15_2021_H5N1
87
1937663_A_turkey_Poland_H1944-N_2021_11_08_2021_H5N1
1923192_A_turkey_Italy_21VIR8585-1_2021_10_18_2021_H5N1
1995228_A_turkey_Italy_21VIR9520_2021_11_14_2021_H5N1
2017737_A_turkey_Italy_21VIR11507_2021_12_20_2021_H5N1
1949331_A_mute_swan_Croatia_144_2021_12_27_2021_H5N1
1966946_A_Mallard_Netherlands_17_2021_12_29_2021_H5N3
2015069_A_gray_heron_Denmark_24326-1.02_2021-10-28_10_28_2021_H5N1
A_Cygnus_columbianus_Hubei_128_2021_H5N1
A_Cygnus_columbianus_Hubei_123_2021_H5N1_
A_Cygnus_columbianus_Hubei_127_2021_H5N1_
100
A_Cygnus_columbianus_Hubei_126_2021_H5N1_
A_Cygnus_columbianus_Hubei_117_2021_H5N1_
1997262_A_duck_Bangladesh_19D1818_2021_12_22_2021_H5N1
1957985_A_chicken_Tyumen_47-79V_2021_10_12_2021_H5N1
A_Cygnus_columbianus_Hubei_121_2021_H5N1_
1958113_A_chicken_Tyumen_81-97V_2021_10_30_2021_H5N1
1958105_A_turkey_Tyumen_81-96V_2021_10_30_2021_H5N1
2021932_A_Ezo_red_fox_Hokkaido_1_2022_03_31_2022_H5N1
2021940_A_crow_Hokkaido_0103B065_2022_03_29_2022_H5N1
1999383_A_white-tailed_eagle_Hokkaido_22-RU-WTE-2_2022_01_02_2022_H5N1
1896066_A_chicken_Nigeria_VRD21-109_21VIR2370-425_2021_03_01_2021_H5N1
1859921_A_barnacle_goose_Netherlands_21027016-002_2021_04_15_2021_H5N1
1943844_A_turkey_Germany-NI_AI04425_2021_05_03_2021_H5N1
1943845_A_turkey_Germany-NI_AI04373_2021_04_27_2021_H5N1
1943857_A_turkey_Germany-NI_AI04455_2021_05_05_2021_H5N1
1862722_A_barnacle_goose_Netherlands_21028534-002_2021_05_13_2021_H5N1
1859803_A_common_murre_Netherlands_21025491-002_2021_03_17_2021_H5N1
1944198_A_barnacle_goose_Germany-NI_AI01605_2021_02_17_2021_H5N1
1944199_A_chicken_Germany-NI_AI01599_2021_02_23_2021_H5N1
2042405_A_Colorado_18_2022_04_20_2022_H5N1
1945469_A_European_herring_gull_Finland_9722_21VIR7689-13_2021_08_01_2021_H5N1
1945485_A_Eurasian_eagle-owl_Finland_10617_21VIR7689-15_2021_08_01_2021_H5N1
1958565_A_England_215201407_2021_12_26_2021_H5N1
1943854_A_oystercatcher_Germany-NI_AI05047_2021_07_17_2021_H5N1
1850253_A_barnacle_goose_Netherlands_21022611-001_2021_01_31_2021_H5N1
1807259_A_Eurasian_Wigeon_Netherlands_5_2020_10_16_2020_H5N1
1812375_A_eurasian_teal_Netherlands_20016896-013_2020_11_02_2020_H5N1
1812367_A_greylag_goose_Netherlands_20016582-004_2020_10_28_2020_H5N1
1848934_A_pheasant_Scotland_000348_2021_02_10_2021_H5N1
1807243_A_Eurasian_Wigeon_Netherlands_1_2020_10_16_2020_H5N1
1838673_A_chicken_Netherlands_20019879-001005_2020_12_14_2020_H5N1
1807251_A_Eurasian_Wigeon_Netherlands_4_2020_10_16_2020_H5N1
1943846_A_barnacle_goose_Germany-NI_AI03980_2021_04_14_2021_H5N1
1943856_A_barnacle_goose_Germany-NI_AI03914_2021_04_08_2021_H5N1
1859811_A_barnacle_goose_Netherlands_21025769-002_2021_03_22_2021_H5N1
2011624_A_barnacle_goose_Denmark_19027-1.02_2021-04-18_04_18_2021_H5N1
1847708_A_barnacle_goose_Netherlands_21021591-001_2021_01_14_2021_H5N1
1866449_A_chicken_Senegal_21VIR1084-3_2021_12_23_2020_H5N1
1866465_A_chicken_Senegal_21VIR1084-5_2021_12_23_2020_H5N1
1866457_A_chicken_Senegal_21VIR1084-4_2021_12_23_2020_H5N1
1883976_A_Turkey_Hungary_16603_2021__H5N1__04_13_2021_H5N1
1866473_A_great-white_pelican_Senegal_21-67_21VIR1084-8_2021_01_23_2021_H5N1
1858204_A_chicken_Slovakia_Pah10_21VIR1086-5_2021_01_22_2021_H5N5
1858260_A_mute_swan_Austria_21013162_21VIR1085-5_2021_02_10_2021_H5N5
1987733_A_mute_swan_Czech_Republic_4099_2021_02_24_2021_H5N5
1883984_A_eagle_Hungary_8569_2021__H5N5__03_08_2021_H5N5
1858284_A_mute_swan_Slovakia_Pah15_21VIR1086-3_2021_01_29_2021_H5N5
1858196_A_chicken_Romania_10101_21VIR2044-1_2021_02_23_2021_H5N5
1858292_A_mute_swan_Slovakia_Pah6_21VIR1086-2_2021_01_15_2021_H5N5
1847700_A_common_buzzard_Netherlands_21021396-002_2021_01_09_2021_H5N1
1850269_A_common_buzzard_Netherlands_21022834-002_2021_02_01_2021_H5N1
1896058_A_chicken_Nigeria_VRD21-102_21VIR2370-424_2021_03_01_2021_H5N1
1896075_A_chicken_Nigeria_VRD21-37_21VIR2288-2_2021_02_05_2021_H5N1
1896034_A_chicken_Nigeria_VRD21-43_21VIR2288-4_2021_02_10_2021_H5N8
100
1918710_A_Hunan_09911_2021_09_03_2021_H5N6
1921210_A_Hunan_10117_2021_09_11_2021_H5N6
1918708_A_Hunan_09285_2021_08_07_2021_H5N6
100
1921590_A_whooper_swan_Shanxi_4-1_2020_11_10_2020_H5N8
1918700_A_Sichuan_06689_2021_07_05_2021_H5N6
1918712_A_Chongqing_02_2021_07_09_2021_H5N6
A_Astrakhan_3212_2020_H5N8
1369973_A_Fujian-Sanyuan_21099_2017_x_PR8__CNIC-21099__12_25_2017_H5N6
2.3.4.4b
1352813_A_Guangdong_18SF020_2018_09_29_2018_H5N6
2.3.4.4h
1848291_A_Chongqing_00013_2021_01_02_2021_H5N6
1369965_A_Hubei_29578_2016_x_PR8___CNIC-HB29578__04_10_2016_H5N6
2.3.4.4d
866708_A_duck_Hyogo_1_2016_11_14_2016_H5N6
2.3.4.4e
98
895063_A_chicken_Vietnam_NCVD-15A59_2015_08_18_2015_H5N6
2.3.4.4f
569390_A_gyrfalcon_Washington_41088-6_2014_12_08_2014_H5N8
2.3.4.4c
533583_A_Sichuan_26221_2014_04_21_2014_H5N6
2.3.4.4a

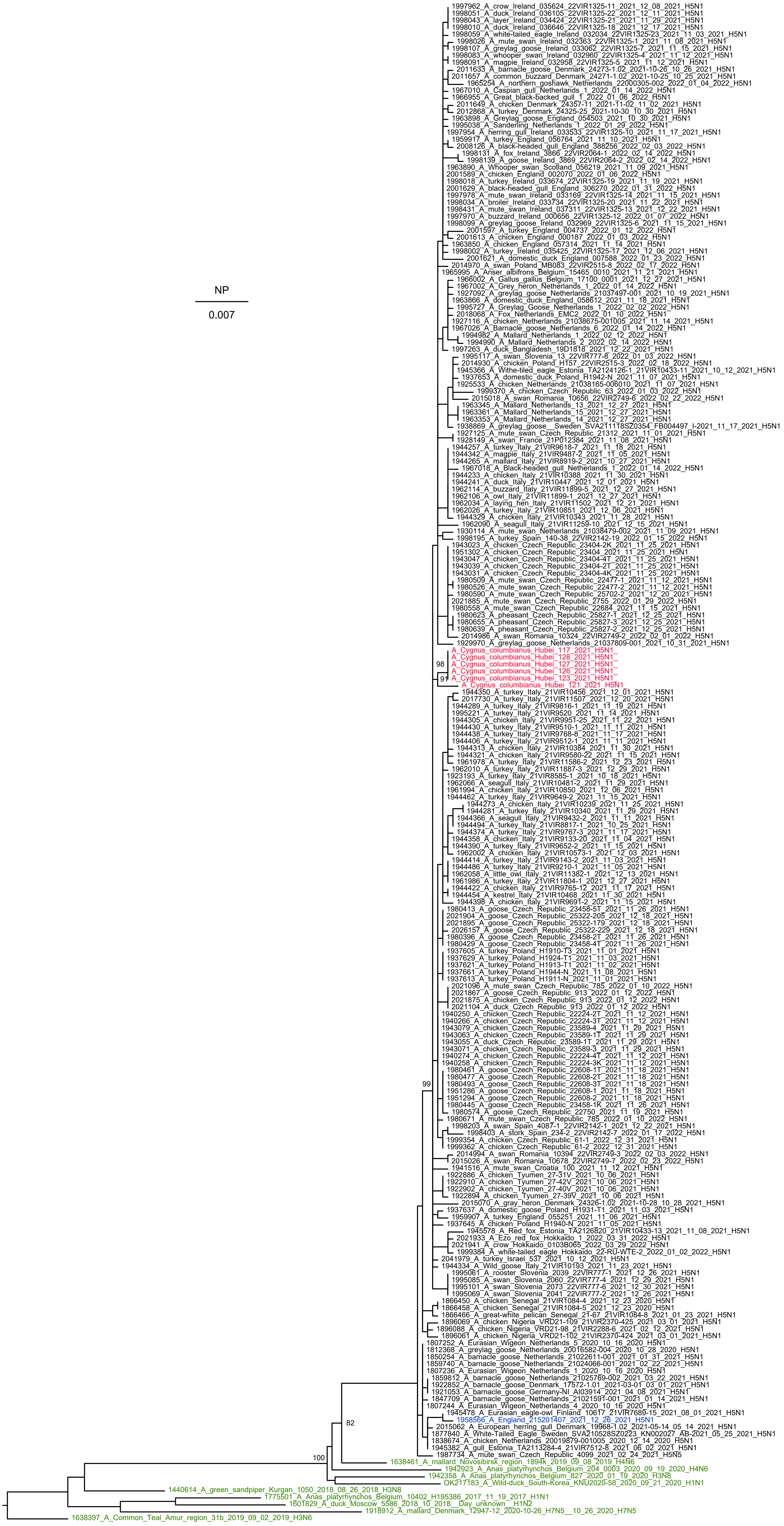

NP
0.007
1997962_A_crow_Ireland_035624_22VIR1325-11_2021_12_08_2021_H5N1
1998051_A_duck_Ireland_036105_22VIR1325-22_2021_12_11_2021_H5N1
1998043_A_layer_Ireland_034424_22VIR1325-21_2021_11_29_2021_H5N1
1998010_A_duck_Ireland_036646_22VIR1325-18_2021_12_17_2021_H5N1
1998059_A_white-tailed_eagle_Ireland_032034_22VIR1325-23_2021_11_03_2021_H5N1
1998026_A_mute_swan_Ireland_032363_22VIR1325-1_2021_11_08_2021_H5N1
1998107_A_greylag_goose_Ireland_033062_22VIR1325-7_2021_11_15_2021_H5N1
1998083_A_whooper_swan_Ireland_032960_22VIR1325-4_2021_11_12_2021_H5N1
1998091_A_magpie_Ireland_032958_22VIR1325-5_2021_11_12_2021_H5N1
2011633_A_barnacle_goose_Denmark_24273-1.02_2021-10-26_10_26_2021_H5N1
2011657_A_common_buzzard_Denmark_24271-1.02_2021-10-25_10_25_2021_H5N1
1965254_A_northern_goshawk_Netherlands_22000305-002_2022_01_04_2022_H5N1
1967010_A_Caspian_gull_Netherlands_1_2022_01_14_2022_H5N1
1966955_A_Great_black-backed_gull_1_2022_01_06_2022_H5N1
2011649_A_chicken_Denmark_24357-11_2021-11-02_11_02_2021_H5N1
2012868_A_turkey_Denmark_24325-25_2021-10-30_10_30_2021_H5N1
1963898_A_Greylag_goose_England_054503_2021_10_30_2021_H5N1
1995038_A_Sanderling_Netherlands_1_2022_01_29_2022_H5N1
1997954_A_herring_gull_Ireland_033533_22VIR1325-10_2021_11_17_2021_H5N1
1959917_A_turkey_England_056764_2021_11_10_2021_H5N1
2008126_A_black-headed_gull_England_388256_2022_02_03_2022_H5N1
1998131_A_fox_Ireland_3866_22VIR2064-1_2022_02_14_2022_H5N1
1998139_A_goose_Ireland_3869_22VIR2064-2_2022_02_14_2022_H5N1
1963890_A_Whooper_swan_Scotland_056219_2021_11_09_2021_H5N1
2001589_A_chicken_England_002070_2022_01_06_2022_H5N1
1998018_A_turkey_Ireland_033674_22VIR1325-19_2021_11_19_2021_H5N1
2001629_A_black-headed_gull_England_306270_2022_01_31_2022_H5N1
1997978_A_mute_swan_Ireland_033169_22VIR1325-14_2021_11_15_2021_H5N1
1998034_A_broiler_Ireland_033734_22VIR1325-20_2021_11_22_2021_H5N1
1998431_A_mute_swan_Ireland_037311_22VIR1325-13_2021_12_22_2021_H5N1
1997970_A_buzzard_Ireland_000656_22VIR1325-12_2022_01_07_2022_H5N1
1998099_A_greylag_goose_Ireland_032969_22VIR1325-6_2021_11_15_2021_H5N1
2001597_A_turkey_England_004737_2022_01_12_2022_H5N1
2001613_A_chicken_England_000187_2022_01_03_2022_H5N1
1963850_A_chicken_England_057314_2021_11_14_2021_H5N1
1998002_A_turkey_Ireland_035425_22VIR1325-17_2021_12_06_2021_H5N1
2001621_A_domestic_duck_England_007588_2022_01_23_2022_H5N1
2014970_A_swan_Poland_MB083_22VIR2515-8_2022_02_17_2022_H5N1
1965995_A_Anser_albifrons_Belgium_15465_0010_2021_11_21_2021_H5N1
1966002_A_Gallus_gallus_Belgium_17100_0001_2021_12_27_2021_H5N1
1967002_A_Grey_heron_Netherlands_1_2022_01_14_2022_H5N1
1927092_A_greylag_goose_Netherlands_21037497-001_2021_10_19_2021_H5N1
1963866_A_domestic_duck_England_058612_2021_11_18_2021_H5N1
1995727_A_Greylag_Goose_Netherlands_1_2022_02_02_2022_H5N1
2018068_A_Fox_Netherlands_EMC2_2022_01_10_2022_H5N1
1927116_A_chicken_Netherlands_21038675-001005_2021_11_14_2021_H5N1
1967026_A_Barnacle_goose_Netherlands_6_2022_01_14_2022_H5N1
1994982_A_Mallard_Netherlands_1_2022_02_12_2022_H5N1
1994990_A_Mallard_Netherlands_2_2022_02_14_2022_H5N1
1997263_A_duck_Bangladesh_19D1818_2021_12_22_2021_H5N1
1995117_A_swan_Slovenia_13_22VIR777-8_2022_01_03_2022_H5N1
2014930_A_chicken_Poland_H157_22VIR2515-3_2022_02_18_2022_H5N1
1945366_A_Withe-tiled_eagle_Estonia_TA2124126-1_21VIR10433-11_2021_10_12_2021_H5N1
1937653_A_domestic_duck_Poland_H1942-N_2021_11_07_2021_H5N1
1925533_A_chicken_Netherlands_21038165-006010_2021_11_07_2021_H5N1
1999370_A_chicken_Czech_Republic_63_2022_01_03_2022_H5N1
2015018_A_swan_Romania_10656_22VIR2749-6_2022_02_22_2022_H5N1
1963345_A_Mallard_Netherlands_13_2021_12_27_2021_H5N1
1963361_A_Mallard_Netherlands_15_2021_12_27_2021_H5N1
1963353_A_Mallard_Netherlands_14_2021_12_27_2021_H5N1
1938869_A_greylag_goose__Sweden_SVA211118SZ0354_FB004497_I-2021_11_17_2021_H5N1
1927125_A_mute_swan_Czech_Republic_21312_2021_11_01_2021_H5N1
1928149_A_swan_France_21P012384_2021_11_08_2021_H5N1
1944257_A_turkey_Italy_21VIR9618-7_2021_11_18_2021_H5N1
1944342_A_magpie_Italy_21VIR9487-2_2021_11_05_2021_H5N1
1944265_A_mallard_Italy_21VIR8919-2_2021_10_27_2021_H5N1
1967018_A_Black-headed_gull_Netherlands_1_2022_01_14_2022_H5N1
1944233_A_chicken_Italy_21VIR10388_2021_11_30_2021_H5N1
1944241_A_duck_Italy_21VIR10447_2021_12_01_2021_H5N1
1962114_A_buzzard_Italy_21VIR11899-5_2021_12_27_2021_H5N1
1962106_A_owl_Italy_21VIR11899-1_2021_12_27_2021_H5N1
1962034_A_laying_hen_Italy_21VIR11502_2021_12_21_2021_H5N1
1962026_A_turkey_Italy_21VIR10851_2021_12_06_2021_H5N1
1944329_A_chicken_Italy_21VIR10343_2021_11_28_2021_H5N1
1962090_A_seagull_Italy_21VIR11259-10_2021_12_15_2021_H5N1
1930114_A_mute_swan_Netherlands_21038479-002_2021_11_09_2021_H5N1
1998195_A_turkey_Spain_140-38_22VIR2142-19_2022_01_15_2022_H5N1
1943023_A_chicken_Czech_Republic_23404-2K_2021_11_25_2021_H5N1
1951302_A_chicken_Czech_Republic_23404_2021_11_25_2021_H5N1
1943047_A_chicken_Czech_Republic_23404-4T_2021_11_25_2021_H5N1
1943039_A_chicken_Czech_Republic_23404-2T_2021_11_25_2021_H5N1
1943031_A_chicken_Czech_Republic_23404-4K_2021_11_25_2021_H5N1
1980509_A_mute_swan_Czech_Republic_22477-1_2021_11_12_2021_H5N1
1980526_A_mute_swan_Czech_Republic_22477-2_2021_11_12_2021_H5N1
1980590_A_mute_swan_Czech_Republic_25702-2_2021_12_20_2021_H5N1
2021885_A_mute_swan_Czech_Republic_2755_2022_01_29_2022_H5N1
1980558_A_mute_swan_Czech_Republic_22684_2021_11_15_2021_H5N1
1980623_A_pheasant_Czech_Republic_25827-1_2021_12_25_2021_H5N1
1980655_A_pheasant_Czech_Republic_25827-3_2021_12_25_2021_H5N1
1980639_A_pheasant_Czech_Republic_25827-2_2021_12_25_2021_H5N1
2014986_A_swan_Romania_10324_22VIR2749-2_2022_02_01_2022_H5N1
1929970_A_greylag_goose_Netherlands_21037809-001_2021_10_31_2021_H5N1
A_Cygnus_columbianus_Hubei_117_2021_H5N1
A_Cygnus_columbianus_Hubei_128_2021_H5N1
98
A_Cygnus_columbianus_Hubei_127_2021_H5N1
A_Cygnus_columbianus_Hubei_126_2021_H5N1
A_Cygnus_columbianus_Hubei_123_2021_H5N1
91
A_Cygnus_columbianus_Hubei_121_2021_H5N1
1944350_A_turkey_Italy_21VIR10456_2021_12_01_2021_H5N1
2017730_A_turkey_Italy_21VIR11507_2021_12_20_2021_H5N1
1944289_A_turkey_Italy_21VIR9816-1_2021_11_19_2021_H5N1
1995221_A_turkey_Italy_21VIR9520_2021_11_14_2021_H5N1
1944305_A_chicken_Italy_21VIR9951-25_2021_11_22_2021_H5N1
1944430_A_turkey_Italy_21VIR9510-1_2021_11_11_2021_H5N1
1944438_A_turkey_Italy_21VIR9768-8_2021_11_17_2021_H5N1
1944406_A_turkey_Italy_21VIR9512-1_2021_11_11_2021_H5N1
1944313_A_chicken_Italy_21VIR10384_2021_11_30_2021_H5N1
1944321_A_chicken_Italy_21VIR9580-22_2021_11_15_2021_H5N1
1961978_A_turkey_Italy_21VIR11586-2_2021_12_23_2021_H5N1
1962010_A_turkey_Italy_21VIR11887-3_2021_12_29_2021_H5N1
1923193_A_turkey_Italy_21VIR8585-1_2021_10_18_2021_H5N1
1962066_A_seagull_Italy_21VIR10481-2_2021_11_29_2021_H5N1
1961994_A_chicken_Italy_21VIR10850_2021_12_06_2021_H5N1
1944462_A_turkey_Italy_21VIR9649-2_2021_11_15_2021_H5N1
1944273_A_chicken_Italy_21VIR10239_2021_11_25_2021_H5N1
1944281_A_turkey_Italy_21VIR10340_2021_11_29_2021_H5N1
1944366_A_seagull_Italy_21VIR9432-2_2021_11_11_2021_H5N1
1944494_A_turkey_Italy_21VIR8817-1_2021_10_25_2021_H5N1
1944374_A_turkey_Italy_21VIR9767-3_2021_11_17_2021_H5N1
1944358_A_chicken_Italy_21VIR9133-20_2021_11_04_2021_H5N1
1944390_A_turkey_Italy_21VIR9652-2_2021_11_15_2021_H5N1
1962002_A_chicken_Italy_21VIR10573-1_2021_12_03_2021_H5N1
1944414_A_turkey_Italy_21VIR9143-2_2021_11_03_2021_H5N1
1944486_A_turkey_Italy_21VIR9210-1_2021_11_05_2021_H5N1
1962058_A_little_owl_Italy_21VIR11382-1_2021_12_13_2021_H5N1
1961986_A_turkey_Italy_21VIR11804-1_2021_12_27_2021_H5N1
1944422_A_chicken_Italy_21VIR9765-12_2021_11_17_2021_H5N1
1944454_A_kestrel_Italy_21VIR10468_2021_11_30_2021_H5N1
1944398_A_chicken_Italy_21VIR9691-2_2021_11_15_2021_H5N1
1980413_A_goose_Czech_Republic_23458-5T_2021_11_26_2021_H5N1
2021904_A_goose_Czech_Republic_25322-205_2021_12_18_2021_H5N1
2021895_A_goose_Czech_Republic_25322-179_2021_12_18_2021_H5N1
2026157_A_goose_Czech_Republic_25322-229_2021_12_18_2021_H5N1
1980396_A_goose_Czech_Republic_23458-2T_2021_11_26_2021_H5N1
1980429_A_goose_Czech_Republic_23458-4T_2021_11_26_2021_H5N1
1937605_A_turkey_Poland_H1910-T3_2021_11_01_2021_H5N1
1937629_A_turkey_Poland_H1924-T1_2021_11_03_2021_H5N1
1937621_A_turkey_Poland_H1913-T1_2021_11_02_2021_H5N1
1937661_A_turkey_Poland_H1944-N_2021_11_08_2021_H5N1
1937613_A_turkey_Poland_H1911-N_2021_11_01_2021_H5N1
2021096_A_mute_swan_Czech_Republic_785_2022_01_10_2022_H5N1
2021867_A_goose_Czech_Republic_913_2022_01_12_2022_H5N1
2021875_A_chicken_Czech_Republic_913_2022_01_12_2022_H5N1
2021104_A_duck_Czech_Republic_913_2022_01_12_2022_H5N1
1940250_A_chicken_Czech_Republic_22224-2T_2021_11_12_2021_H5N1
1940266_A_chicken_Czech_Republic_22224-3T_2021_11_12_2021_H5N1
1943079_A_chicken_Czech_Republic_23589-4_2021_11_29_2021_H5N1
1943063_A_chicken_Czech_Republic_23589-1T_2021_11_29_2021_H5N1
1943055_A_duck_Czech_Republic_23589-1T_2021_11_29_2021_H5N1
1943071_A_chicken_Czech_Republic_23589-3_2021_11_29_2021_H5N1
1940274_A_chicken_Czech_Republic_22224-4T_2021_11_12_2021_H5N1
1940258_A_chicken_Czech_Republic_22224-3K_2021_11_12_2021_H5N1
1980461_A_goose_Czech_Republic_22608-1T_2021_11_18_2021_H5N1
1980477_A_goose_Czech_Republic_22608-2T_2021_11_18_2021_H5N1
1980493_A_goose_Czech_Republic_22608-3T_2021_11_18_2021_H5N1
1951286_A_goose_Czech_Republic_22608-1_2021_11_18_2021_H5N1
1951294_A_goose_Czech_Republic_22608-2_2021_11_18_2021_H5N1
1980445_A_goose_Czech_Republic_23458-1K_2021_11_26_2021_H5N1
1980574_A_goose_Czech_Republic_22750_2021_11_19_2021_H5N1
99
1980671_A_mute_swan_Czech_Republic_785_2022_01_10_2022_H5N1
1998203_A_swan_Spain_4087-1_22VIR2142-1_2021_12_22_2021_H5N1
1998403_A_stork_Spain_234-2_22VIR2142-7_2022_01_17_2022_H5N1
1999354_A_chicken_Czech_Republic_61-1_2022_12_31_2021_H5N1
1999362_A_chicken_Czech_Republic_61-2_2022_12_31_2021_H5N1
2014994_A_swan_Romania_10394_22VIR2749-3_2022_02_03_2022_H5N1
2015026_A_swan_Romania_10678_22VIR2749-7_2022_02_23_2022_H5N1
1941516_A_mute_swan_Croatia_100_2021_11_12_2021_H5N1
1922886_A_chicken_Tyumen_27-31V_2021_10_06_2021_H5N1
1922910_A_chicken_Tyumen_27-42V_2021_10_06_2021_H5N1
1922902_A_chicken_Tyumen_27-40V_2021_10_06_2021_H5N1
1922894_A_chicken_Tyumen_27-39V_2021_10_06_2021_H5N1
2015070_A_gray_heron_Denmark_24326-1.02_2021-10-28_10_28_2021_H5N1
1937637_A_domestic_goose_Poland_H1931-T1_2021_11_03_2021_H5N1
1959907_A_turkey_England_055251_2021_11_06_2021_H5N1
1937645_A_chicken_Poland_H1940-N_2021_11_05_2021_H5N1
1945578_A_Red_fox_Estonia_TA2126820_21VIR10433-13_2021_11_08_2021_H5N1
2021933_A_Ezo_red_fox_Hokkaido_1_2022_03_31_2022_H5N1
2021941_A_crow_Hokkaido_0103B065_2022_03_29_2022_H5N1
1999384_A_white-tailed_eagle_Hokkaido_22-RU-WTE-2_2022_01_02_2022_H5N1
2041979_A_turkey_Israel_537_2021_10_12_2021_H5N1
1944334_A_Wild_goose_Italy_21VIR10193_2021_11_23_2021_H5N1
1995061_A_rooster_Slovenia_2039_22VIR777-1_2021_12_26_2021_H5N1
1995085_A_swan_Slovenia_2060_22VIR777-4_2021_12_29_2021_H5N1
1995101_A_swan_Slovenia_2073_22VIR777-6_2021_12_30_2021_H5N1
1995069_A_swan_Slovenia_2041_22VIR777-2_2021_12_26_2021_H5N1
1866450_A_chicken_Senegal_21VIR1084-4_2021_12_23_2020_H5N1
1866458_A_chicken_Senegal_21VIR1084-5_2021_12_23_2020_H5N1
1866466_A_great-white_pelican_Senegal_21-67_21VIR1084-8_2021_01_23_2021_H5N1
1896069_A_chicken_Nigeria_VRD21-109_21VIR2370-425_2021_03_01_2021_H5N1
1896088_A_chicken_Nigeria_VRD21-98_21VIR2288-6_2021_02_12_2021_H5N1
1896061_A_chicken_Nigeria_VRD21-102_21VIR2370-424_2021_03_01_2021_H5N1
1807252_A_Eurasian_Wigeon_Netherlands_5_2020_10_16_2020_H5N1
1812368_A_greylag_goose_Netherlands_20016582-004_2020_10_28_2020_H5N1
1850254_A_barnacle_goose_Netherlands_21022611-001_2021_01_31_2021_H5N1
1859740_A_barnacle_goose_Netherlands_21024066-001_2021_02_22_2021_H5N1
1807236_A_Eurasian_Wigeon_Netherlands_1_2020_10_16_2020_H5N1
1859812_A_barnacle_goose_Netherlands_21025769-002_2021_03_22_2021_H5N1
1922852_A_barnacle_goose_Denmark_17572-1.01_2021-03-01_03_01_2021_H5N1
1921053_A_barnacle_goose_Germany-NI_AI03914_2021_04_08_2021_H5N1
1847709_A_barnacle_goose_Netherlands_21021591-001_2021_01_14_2021_H5N1
1807244_A_Eurasian_Wigeon_Netherlands_4_2020_10_16_2020_H5N1
1945478_A_Eurasian_eagle-owl_Finland_10617_21VIR7689-15_2021_08_01_2021_H5N1
82
1958566_A_England_215201407_2021_12_26_2021_H5N1
2015062_A_European_herring_gull_Denmark_19968-1.02_2021-05-14_05_14_2021_H5N1
1877840_A_White-Tailed_Eagle_Sweden_SVA210528SZ0223_KN002027_AB-2021_05_25_2021_H5N1
1838674_A_chicken_Netherlands_20019879-001005_2020_12_14_2020_H5N1
1945382_A_gull_Estonia_TA2113284-4_21VIR7512-8_2021_06_02_2021_H5N1
1987734_A_mute_swan_Czech_Republic_4099_2021_02_24_2021_H5N5
100
1638461_A_mallard_Novosibirsk_region_1894k_2019_09_08_2019_H4N6
1942923_A_Anas_platyrhynchos_Belgium_204_0003_2020_09_19_2020_H4N6
1942358_A_Anas_platyrhynchos_Belgium_827_2020_01_19_2020_H3N8
OK217183_A_Wild-duck_South-Korea_KNU2020-58_2020_09_21_2020_H1N1
1440614_A_green_sandpiper_Kurgan_1050_2018_08_26_2018_H3N8
1775501_A_Anas_platyrhynchos_Belgium_10402_H195386_2017_11_19_2017_H1N1
1801829_A_duck_Moscow_5586_2018_10_2018__Day_unknown__H1N2
1918912_A_mallard_Denmark_12947-12_2020-10-26_H7N5__10_26_2020_H7N5
1638397_A_Common_Teal_Amur_region_31b_2019_09_02_2019_H3N6

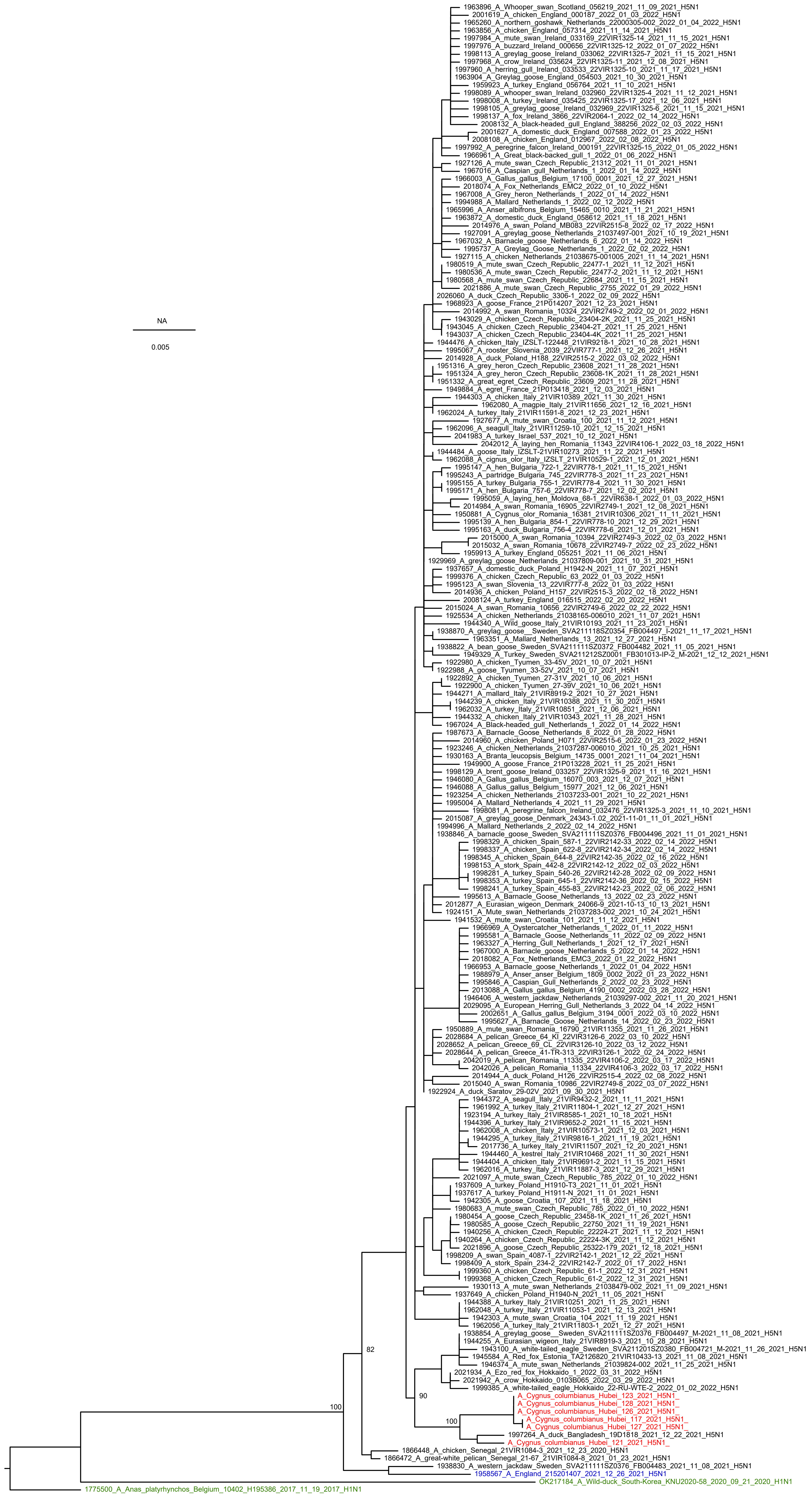
NA
0.005
1963896_A_Whooper_swan_Scotland_056219_2021_11_09_2021_H5N1
2001619_A_chicken_England_000187_2022_01_03_2022_H5N1
1965260_A_northern_goshawk_Netherlands_22000305-002_2022_01_04_2022_H5N1
1963856_A_chicken_England_057314_2021_11_14_2021_H5N1
1997984_A_mute_swan_Ireland_033169_22VIR1325-14_2021_11_15_2021_H5N1
1997976_A_buzzard_Ireland_000656_22VIR1325-12_2022_01_07_2022_H5N1
1998113_A_greylag_goose_Ireland_033062_22VIR1325-7_2021_11_15_2021_H5N1
1997968_A_crow_Ireland_035624_22VIR1325-11_2021_12_08_2021_H5N1
1997960_A_herring_gull_Ireland_033533_22VIR1325-10_2021_11_17_2021_H5N1
1963904_A_Greylag_goose_England_054503_2021_10_30_2021_H5N1
1959923_A_turkey_England_056764_2021_11_10_2021_H5N1
1998089_A_whooper_swan_Ireland_032960_22VIR1325-4_2021_11_12_2021_H5N1
1998008_A_turkey_Ireland_035425_22VIR1325-17_2021_12_06_2021_H5N1
1998105_A_greylag_goose_Ireland_032969_22VIR1325-6_2021_11_15_2021_H5N1
1998137_A_fox_Ireland_3866_22VIR2064-1_2022_02_14_2022_H5N1
2008132_A_black-headed_gull_England_388256_2022_02_03_2022_H5N1
2001627_A_domestic_duck_England_007588_2022_01_23_2022_H5N1
2008108_A_chicken_England_012967_2022_02_08_2022_H5N1
1997992_A_peregrine_falcon_Ireland_000191_22VIR1325-15_2022_01_05_2022_H5N1
1966961_A_Great_black-backed_gull_1_2022_01_06_2022_H5N1
1927126_A_mute_swan_Czech_Republic_21312_2021_11_01_2021_H5N1
1967016_A_Caspian_gull_Netherlands_1_2022_01_14_2022_H5N1
1966003_A_Gallus_gallus_Belgium_17100_0001_2021_12_27_2021_H5N1
2018074_A_Fox_Netherlands_EMC2_2022_01_10_2022_H5N1
1967008_A_Grey_heron_Netherlands_1_2022_01_14_2022_H5N1
1994988_A_Mallard_Netherlands_1_2022_02_12_2022_H5N1
1965996_A_Anser_albifrons_Belgium_15465_0010_2021_11_21_2021_H5N1
1963872_A_domestic_duck_England_058612_2021_11_18_2021_H5N1
2014976_A_swan_Poland_MB083_22VIR2515-8_2022_02_17_2022_H5N1
1927091_A_greylag_goose_Netherlands_21037497-001_2021_10_19_2021_H5N1
1967032_A_Barnacle_goose_Netherlands_6_2022_01_14_2022_H5N1
1995737_A_Greylag_Goose_Netherlands_1_2022_02_02_2022_H5N1
1927115_A_chicken_Netherlands_21038675-001005_2021_11_14_2021_H5N1
1980519_A_mute_swan_Czech_Republic_22477-1_2021_11_12_2021_H5N1
1980536_A_mute_swan_Czech_Republic_22477-2_2021_11_12_2021_H5N1
1980568_A_mute_swan_Czech_Republic_22684_2021_11_15_2021_H5N1
2021886_A_mute_swan_Czech_Republic_2755_2022_01_29_2022_H5N1
2026060_A_duck_Czech_Republic_3306-1_2022_02_09_2022_H5N1
1968923_A_goose_France_21P014207_2021_12_23_2021_H5N1
2014992_A_swan_Romania_10324_22VIR2749-2_2022_02_01_2022_H5N1
1943029_A_chicken_Czech_Republic_23404-2K_2021_11_25_2021_H5N1
1943045_A_chicken_Czech_Republic_23404-2T_2021_11_25_2021_H5N1
1943037_A_chicken_Czech_Republic_23404-4K_2021_11_25_2021_H5N1
1944476_A_chicken_Italy_IZSLT-122448_21VIR9218-1_2021_10_28_2021_H5N1
1995067_A_rooster_Slovenia_2039_22VIR777-1_2021_12_26_2021_H5N1
2014928_A_duck_Poland_H188_22VIR2515-2_2022_03_02_2022_H5N1
1951316_A_grey_heron_Czech_Republic_23608_2021_11_28_2021_H5N1
1951324_A_grey_heron_Czech_Republic_23608-1K_2021_11_28_2021_H5N1
1951332_A_great_egret_Czech_Republic_23609_2021_11_28_2021_H5N1
1949884_A_egret_France_21P013418_2021_12_03_2021_H5N1
1944303_A_chicken_Italy_21VIR10389_2021_11_30_2021_H5N1
1962080_A_magpie_Italy_21VIR11656_2021_12_16_2021_H5N1
1962024_A_turkey_Italy_21VIR11591-8_2021_12_23_2021_H5N1
1927677_A_mute_swan_Croatia_100_2021_11_12_2021_H5N1
1962096_A_seagull_Italy_21VIR11259-10_2021_12_15_2021_H5N1
2041983_A_turkey_Israel_537_2021_10_12_2021_H5N1
2042012_A_laying_hen_Romania_11343_22VIR4106-1_2022_03_18_2022_H5N1
1944484_A_goose_Italy_IZSLT-21VIR10273_2021_11_22_2021_H5N1
1962088_A_cignus_olor_Italy_IZSLT_21VIR10529-1_2021_12_01_2021_H5N1
1995147_A_hen_Bulgaria_722-1_22VIR778-1_2021_11_15_2021_H5N1
1995243_A_partridge_Bulgaria_745_22VIR778-3_2021_11_23_2021_H5N1
1995155_A_turkey_Bulgaria_755-1_22VIR778-4_2021_11_30_2021_H5N1
1995171_A_hen_Bulgaria_757-6_22VIR778-7_2021_12_02_2021_H5N1
1995059_A_laying_hen_Moldova_68-1_22VIR638-1_2022_01_03_2022_H5N1
2014984_A_swan_Romania_16905_22VIR2749-1_2021_12_08_2021_H5N1
1950881_A_Cygnus_olor_Romania_16381_21VIR10306_2021_11_11_2021_H5N1
1995139_A_hen_Bulgaria_854-1_22VIR778-10_2021_12_29_2021_H5N1
1995163_A_duck_Bulgaria_756-4_22VIR778-6_2021_12_01_2021_H5N1
2015000_A_swan_Romania_10394_22VIR2749-3_2022_02_03_2022_H5N1
2015032_A_swan_Romania_10678_22VIR2749-7_2022_02_23_2022_H5N1
1959913_A_turkey_England_055251_2021_11_06_2021_H5N1
1929969_A_greylag_goose_Netherlands_21037809-001_2021_10_31_2021_H5N1
1937657_A_domestic_duck_Poland_H1942-N_2021_11_07_2021_H5N1
1999376_A_chicken_Czech_Republic_63_2022_01_03_2022_H5N1
1995123_A_swan_Slovenia_13_22VIR777-8_2022_01_03_2022_H5N1
2014936_A_chicken_Poland_H157_22VIR2515-3_2022_02_18_2022_H5N1
2008124_A_turkey_England_016515_2022_02_20_2022_H5N1
2015024_A_swan_Romania_10656_22VIR2749-6_2022_02_22_2022_H5N1
1925534_A_chicken_Netherlands_21038165-006010_2021_11_07_2021_H5N1
1944340_A_Wild_goose_Italy_21VIR10193_2021_11_23_2021_H5N1
1938870_A_greylag_goose__Sweden_SVA211118SZ0354_FB004497_I-2021_11_17_2021_H5N1
1963351_A_Mallard_Netherlands_13_2021_12_27_2021_H5N1
1938822_A_bean_goose_Sweden_SVA211111SZ0372_FB004482_2021_11_05_2021_H5N1
1949329_A_Turkey_Sweden_SVA211212SZ0001_FB301013-IP-2_M-2021_12_12_2021_H5N1
1922980_A_chicken_Tyumen_33-45V_2021_10_07_2021_H5N1
1922988_A_goose_Tyumen_33-52V_2021_10_07_2021_H5N1
1922892_A_chicken_Tyumen_27-31V_2021_10_06_2021_H5N1
1922900_A_chicken_Tyumen_27-39V_2021_10_06_2021_H5N1
1944271_A_mallard_Italy_21VIR8919-2_2021_10_27_2021_H5N1
1944239_A_chicken_Italy_21VIR10388_2021_11_30_2021_H5N1
1962032_A_turkey_Italy_21VIR10851_2021_12_06_2021_H5N1
1944332_A_chicken_Italy_21VIR10343_2021_11_28_2021_H5N1
1967024_A_Black-headed_gull_Netherlands_1_2022_01_14_2022_H5N1
1987673_A_Barnacle_Goose_Netherlands_8_2022_01_28_2022_H5N1
2014960_A_chicken_Poland_H071_22VIR2515-6_2022_01_23_2022_H5N1
1923246_A_chicken_Netherlands_21037287-006010_2021_10_25_2021_H5N1
1930163_A_Branta_leucopsis_Belgium_14735_0001_2021_11_04_2021_H5N1
1949900_A_goose_France_21P013228_2021_11_25_2021_H5N1
1998129_A_brent_goose_Ireland_033257_22VIR1325-9_2021_11_16_2021_H5N1
1946080_A_Gallus_gallus_Belgium_16070_003_2021_12_07_2021_H5N1
1946088_A_Gallus_gallus_Belgium_15977_2021_12_06_2021_H5N1
1923254_A_chicken_Netherlands_21037233-001_2021_10_22_2021_H5N1
1995004_A_Mallard_Netherlands_4_2021_11_29_2021_H5N1
1998081_A_peregrine_falcon_Ireland_032476_22VIR1325-3_2021_11_10_2021_H5N1
2015087_A_greylag_goose_Denmark_24343-1.02_2021-11-01_11_01_2021_H5N1
1994996_A_Mallard_Netherlands_2_2022_02_14_2022_H5N1
1938846_A_barnacle_goose_Sweden_SVA211111SZ0376_FB004496_2021_11_01_2021_H5N1
1998329_A_chicken_Spain_587-1_22VIR2142-33_2022_02_14_2022_H5N1
1998337_A_chicken_Spain_622-8_22VIR2142-34_2022_02_14_2022_H5N1
1998345_A_chicken_Spain_644-8_22VIR2142-35_2022_02_16_2022_H5N1
1998153_A_stork_Spain_442-8_22VIR2142-12_2022_02_03_2022_H5N1
1998281_A_turkey_Spain_540-26_22VIR2142-28_2022_02_09_2022_H5N1
1998353_A_turkey_Spain_645-1_22VIR2142-36_2022_02_15_2022_H5N1
1998241_A_turkey_Spain_455-83_22VIR2142-23_2022_02_06_2022_H5N1
1995613_A_Barnacle_Goose_Netherlands_13_2022_02_23_2022_H5N1
2012877_A_Eurasian_wigeon_Denmark_24066-9_2021-10-13_10_13_2021_H5N1
1924151_A_Mute_swan_Netherlands_21037283-002_2021_10_24_2021_H5N1
1941532_A_mute_swan_Croatia_101_2021_11_12_2021_H5N1
1966969_A_Oystercatcher_Netherlands_1_2022_01_11_2022_H5N1
1995581_A_Barnacle_Goose_Netherlands_11_2022_02_09_2022_H5N1
1963327_A_Herring_Gull_Netherlands_1_2021_12_17_2021_H5N1
1967000_A_Barnacle_goose_Netherlands_5_2022_01_14_2022_H5N1
2018082_A_Fox_Netherlands_EMC3_2022_01_22_2022_H5N1
1966953_A_Barnacle_goose_Netherlands_1_2022_01_04_2022_H5N1
1988979_A_Anser_anser_Belgium_1809_0002_2022_01_23_2022_H5N1
1995846_A_Caspian_Gull_Netherlands_2_2022_02_23_2022_H5N1
2013088_A_Gallus_gallus_Belgium_4190_0002_2022_03_28_2022_H5N1
1946406_A_western_jackdaw_Netherlands_21039297-002_2021_11_20_2021_H5N1
2029095_A_European_Herring_Gull_Netherlands_3_2022_04_14_2022_H5N1
2002651_A_Gallus_gallus_Belgium_3194_0001_2022_03_10_2022_H5N1
1995627_A_Barnacle_Goose_Netherlands_14_2022_02_23_2022_H5N1
1950889_A_mute_swan_Romania_16790_21VIR11355_2021_11_26_2021_H5N1
2028684_A_pelican_Greece_64_KI_22VIR3126-6_2022_03_10_2022_H5N1
2028652_A_pelican_Greece_69_CL_22VIR3126-10_2022_03_12_2022_H5N1
2028644_A_pelican_Greece_41-TR-313_22VIR3126-1_2022_02_24_2022_H5N1
2042019_A_pelican_Romania_11335_22VIR4106-2_2022_03_17_2022_H5N1
2042026_A_pelican_Romania_11334_22VIR4106-3_2022_03_17_2022_H5N1
2014944_A_duck_Poland_H126_22VIR2515-4_2022_02_08_2022_H5N1
2015040_A_swan_Romania_10986_22VIR2749-8_2022_03_07_2022_H5N1
1922924_A_duck_Saratov_29-02V_2021_09_30_2021_H5N1
1944372_A_seagull_Italy_21VIR9432-2_2021_11_11_2021_H5N1
1961992_A_turkey_Italy_21VIR11804-1_2021_12_27_2021_H5N1
1923194_A_turkey_Italy_21VIR8585-1_2021_10_18_2021_H5N1
1944396_A_turkey_Italy_21VIR9652-2_2021_11_15_2021_H5N1
1962008_A_chicken_Italy_21VIR10573-1_2021_12_03_2021_H5N1
1944295_A_turkey_Italy_21VIR9816-1_2021_11_19_2021_H5N1
2017736_A_turkey_Italy_21VIR11507_2021_12_20_2021_H5N1
1944460_A_kestrel_Italy_21VIR10468_2021_11_30_2021_H5N1
1944404_A_chicken_Italy_21VIR9691-2_2021_11_15_2021_H5N1
1962016_A_turkey_Italy_21VIR11887-3_2021_12_29_2021_H5N1
2021097_A_mute_swan_Czech_Republic_785_2022_01_10_2022_H5N1
1937609_A_turkey_Poland_H1910-T3_2021_11_01_2021_H5N1
1937617_A_turkey_Poland_H1911-N_2021_11_01_2021_H5N1
1942305_A_goose_Croatia_107_2021_11_18_2021_H5N1
1980683_A_mute_swan_Czech_Republic_785_2022_01_10_2022_H5N1
1980454_A_goose_Czech_Republic_23458-1K_2021_11_26_2021_H5N1
1980585_A_goose_Czech_Republic_22750_2021_11_19_2021_H5N1
1940256_A_chicken_Czech_Republic_22224-2T_2021_11_12_2021_H5N1
1940264_A_chicken_Czech_Republic_22224-3K_2021_11_12_2021_H5N1
2021896_A_goose_Czech_Republic_25322-179_2021_12_18_2021_H5N1
1998209_A_swan_Spain_4087-1_22VIR2142-1_2021_12_22_2021_H5N1
1998409_A_stork_Spain_234-2_22VIR2142-7_2022_01_17_2022_H5N1
1999360_A_chicken_Czech_Republic_61-1_2022_12_31_2021_H5N1
1999368_A_chicken_Czech_Republic_61-2_2022_12_31_2021_H5N1
1930113_A_mute_swan_Netherlands_21038479-002_2021_11_09_2021_H5N1
1937649_A_chicken_Poland_H1940-N_2021_11_05_2021_H5N1
1944388_A_turkey_Italy_21VIR10251_2021_11_25_2021_H5N1
1962048_A_turkey_Italy_21VIR11053-1_2021_12_13_2021_H5N1
1942303_A_mute_swan_Croatia_104_2021_11_19_2021_H5N1
1962056_A_turkey_Italy_21VIR11803-1_2021_12_27_2021_H5N1
1938854_A_greylag_goose__Sweden_SVA211111SZ0376_FB004497_M-2021_11_08_2021_H5N1
1944255_A_Eurasian_wigeon_Italy_21VIR8919-3_2021_10_28_2021_H5N1
1943100_A_white-tailed_eagle_Sweden_SVA211201SZ0380_FB004721_M-2021_11_26_2021_H5N1
1945584_A_Red_fox_Estonia_TA2126820_21VIR10433-13_2021_11_08_2021_H5N1
1946374_A_mute_swan_Netherlands_21039824-002_2021_11_25_2021_H5N1
2021934_A_Ezo_red_fox_Hokkaido_1_2022_03_31_2022_H5N1
2021942_A_crow_Hokkaido_0103B065_2022_03_29_2022_H5N1
1999385_A_white-tailed_eagle_Hokkaido_22-RU-WTE-2_2022_01_02_2022_H5N1
82
90
A_Cygnus_columbianus_Hubei_123_2021_H5N1_
A_Cygnus_columbianus_Hubei_128_2021_H5N1_
A_Cygnus_columbianus_Hubei_126_2021_H5N1_
100
A_Cygnus_columbianus_Hubei_117_2021_H5N1_
A_Cygnus_columbianus_Hubei_127_2021_H5N1_
1997264_A_duck_Bangladesh_19D1818_2021_12_22_2021_H5N1
A_Cygnus_columbianus_Hubei_121_2021_H5N1_
100
1866448_A_chicken_Senegal_21VIR1084-3_2021_12_23_2020_H5N1
1866472_A_great-white_pelican_Senegal_21-67_21VIR1084-8_2021_01_23_2021_H5N1
1938830_A_western_jackdaw_Sweden_SVA211111SZ0376_FB004483_2021_11_08_2021_H5N1
1958567_A_England_215201407_2021_12_26_2021_H5N1
OK217184_A_Wild-duck_South-Korea_KNU2020-58_2020_09_21_2020_H1N1
1775500_A_Anas_platyrhynchos_Belgium_10402_H195386_2017_11_19_2017_H1N1

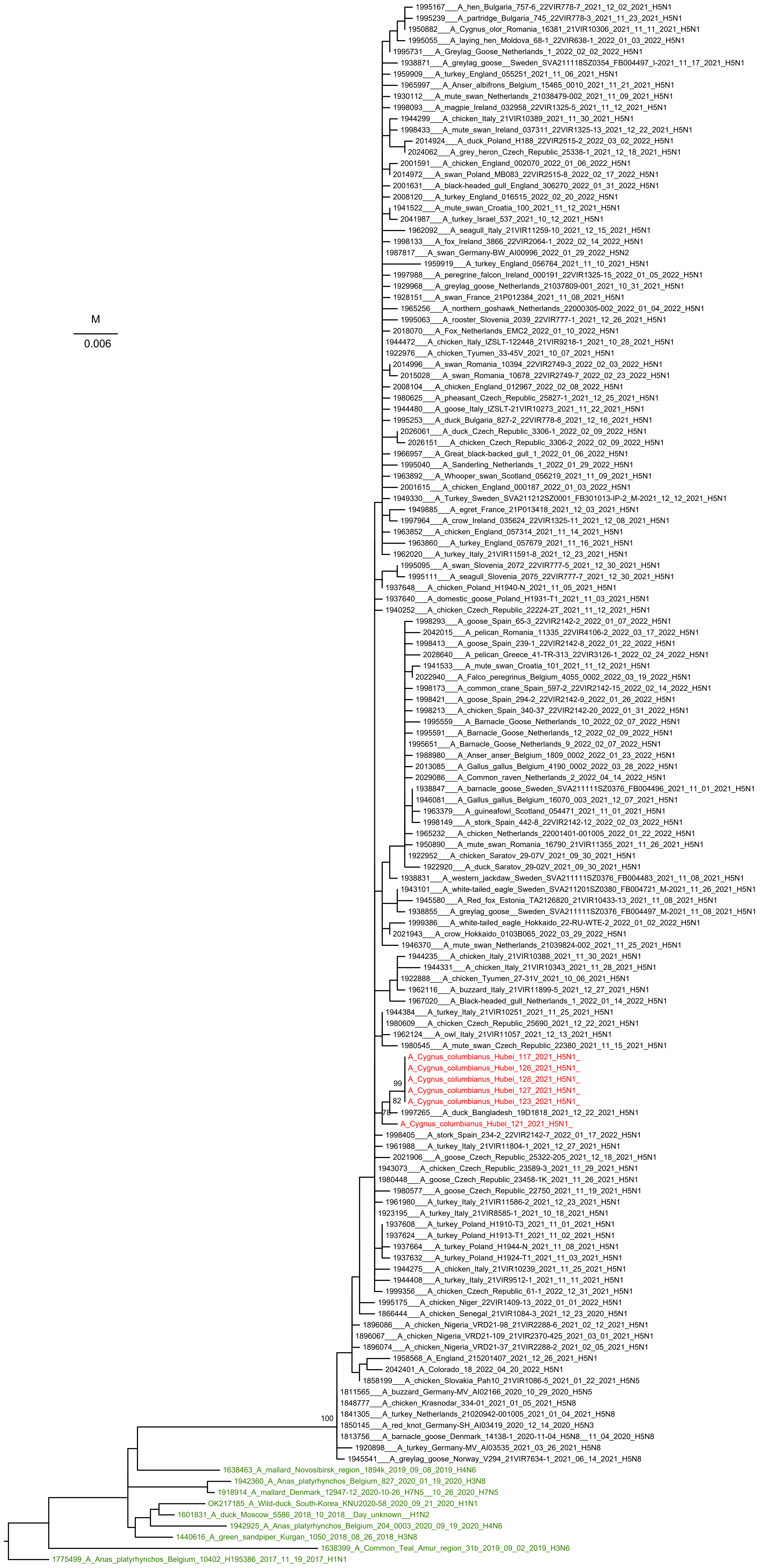

M
0.006
1995167___A_hen_Bulgaria_757-6_22VIR778-7_2021_12_02_2021_H5N1
1995239___A_partridge_Bulgaria_745_22VIR778-3_2021_11_23_2021_H5N1
1950882___A_Cygnus_olor_Romania_16381_21VIR10306_2021_11_11_2021_H5N1
1995055___A_laying_hen_Moldova_68-1_22VIR638-1_2022_01_03_2022_H5N1
1995731___A_Greylag_Goose_Netherlands_1_2022_02_02_2022_H5N1
1938871___A_greylag_goose__Sweden_SVA211118SZ0354_FB004497_I-2021_11_17_2021_H5N1
1959909___A_turkey_England_055251_2021_11_06_2021_H5N1
1965997___A_Anser_albifrons_Belgium_15465_0010_2021_11_21_2021_H5N1
1930112___A_mute_swan_Netherlands_21038479-002_2021_11_09_2021_H5N1
1998093___A_magpie_Ireland_032958_22VIR1325-5_2021_11_12_2021_H5N1
1944299___A_chicken_Italy_21VIR10389_2021_11_30_2021_H5N1
1998433___A_mute_swan_Ireland_037311_22VIR1325-13_2021_12_22_2021_H5N1
2014924___A_duck_Poland_H188_22VIR2515-2_2022_03_02_2022_H5N1
2024062___A_grey_heron_Czech_Republic_25338-1_2021_12_18_2021_H5N1
2001591___A_chicken_England_002070_2022_01_06_2022_H5N1
2014972___A_swan_Poland_MB083_22VIR2515-8_2022_02_17_2022_H5N1
2001631___A_black-headed_gull_England_306270_2022_01_31_2022_H5N1
2008120___A_turkey_England_016515_2022_02_20_2022_H5N1
1941522___A_mute_swan_Croatia_100_2021_11_12_2021_H5N1
2041987___A_turkey_Israel_537_2021_10_12_2021_H5N1
1962092___A_seagull_Italy_21VIR11259-10_2021_12_15_2021_H5N1
1998133___A_fox_Ireland_3866_22VIR2064-1_2022_02_14_2022_H5N1
1987817___A_swan_Germany-BW_AI00996_2022_01_29_2022_H5N2
1959919___A_turkey_England_056764_2021_11_10_2021_H5N1
1997988___A_peregrine_falcon_Ireland_000191_22VIR1325-15_2022_01_05_2022_H5N1
1929968___A_greylag_goose_Netherlands_21037809-001_2021_10_31_2021_H5N1
1928151___A_swan_France_21P012384_2021_11_08_2021_H5N1
1965256___A_northern_goshawk_Netherlands_22000305-002_2022_01_04_2022_H5N1
1995063___A_rooster_Slovenia_2039_22VIR777-1_2021_12_26_2021_H5N1
2018070___A_Fox_Netherlands_EMC2_2022_01_10_2022_H5N1
1944472___A_chicken_Italy_IZSLT-122448_21VIR9218-1_2021_10_28_2021_H5N1
1922976___A_chicken_Tyumen_33-45V_2021_10_07_2021_H5N1
2014996___A_swan_Romania_10394_22VIR2749-3_2022_02_03_2022_H5N1
2015028___A_swan_Romania_10678_22VIR2749-7_2022_02_23_2022_H5N1
2008104___A_chicken_England_012967_2022_02_08_2022_H5N1
1980625___A_pheasant_Czech_Republic_25827-1_2021_12_25_2021_H5N1
1944480___A_goose_Italy_IZSLT-21VIR10273_2021_11_22_2021_H5N1
1995253___A_duck_Bulgaria_827-2_22VIR778-8_2021_12_16_2021_H5N1
2026061___A_duck_Czech_Republic_3306-1_2022_02_09_2022_H5N1
2026151___A_chicken_Czech_Republic_3306-2_2022_02_09_2022_H5N1
1966957___A_Great_black-backed_gull_1_2022_01_06_2022_H5N1
1995040___A_Sanderling_Netherlands_1_2022_01_29_2022_H5N1
1963892___A_Whooper_swan_Scotland_056219_2021_11_09_2021_H5N1
2001615___A_chicken_England_000187_2022_01_03_2022_H5N1
1949330___A_Turkey_Sweden_SVA211212SZ0001_FB301013-IP-2_M-2021_12_12_2021_H5N1
1949885___A_egret_France_21P013418_2021_12_03_2021_H5N1
1997964___A_crow_Ireland_035624_22VIR1325-11_2021_12_08_2021_H5N1
1963852___A_chicken_England_057314_2021_11_14_2021_H5N1
1963860___A_turkey_England_057679_2021_11_16_2021_H5N1
1962020___A_turkey_Italy_21VIR11591-8_2021_12_23_2021_H5N1
1995095___A_swan_Slovenia_2072_22VIR777-5_2021_12_30_2021_H5N1
1995111___A_seagull_Slovenia_2075_22VIR777-7_2021_12_30_2021_H5N1
1937648___A_chicken_Poland_H1940-N_2021_11_05_2021_H5N1
1937640___A_domestic_goose_Poland_H1931-T1_2021_11_03_2021_H5N1
1940252___A_chicken_Czech_Republic_22224-2T_2021_11_12_2021_H5N1
1998293___A_goose_Spain_65-3_22VIR2142-2_2022_01_07_2022_H5N1
2042015___A_pelican_Romania_11335_22VIR4106-2_2022_03_17_2022_H5N1
1998413___A_goose_Spain_239-1_22VIR2142-8_2022_01_22_2022_H5N1
2028640___A_pelican_Greece_41-TR-313_22VIR3126-1_2022_02_24_2022_H5N1
1941533___A_mute_swan_Croatia_101_2021_11_12_2021_H5N1
2022940___A_Falco_peregrinus_Belgium_4055_0002_2022_03_19_2022_H5N1
1998173___A_common_crane_Spain_597-2_22VIR2142-15_2022_02_14_2022_H5N1
1998421___A_goose_Spain_294-2_22VIR2142-9_2022_01_26_2022_H5N1
1998213___A_chicken_Spain_340-37_22VIR2142-20_2022_01_31_2022_H5N1
1995559___A_Barnacle_Goose_Netherlands_10_2022_02_07_2022_H5N1
1995591___A_Barnacle_Goose_Netherlands_12_2022_02_09_2022_H5N1
1995651___A_Barnacle_Goose_Netherlands_9_2022_02_07_2022_H5N1
1988980___A_Anser_anser_Belgium_1809_0002_2022_01_23_2022_H5N1
2013085___A_Gallus_gallus_Belgium_4190_0002_2022_03_28_2022_H5N1
2029086___A_Common_raven_Netherlands_2_2022_04_14_2022_H5N1
1938847___A_barnacle_goose_Sweden_SVA211111SZ0376_FB004496_2021_11_01_2021_H5N1
1946081___A_Gallus_gallus_Belgium_16070_003_2021_12_07_2021_H5N1
1963379___A_guineafowl_Scotland_054471_2021_11_01_2021_H5N1
1998149___A_stork_Spain_442-8_22VIR2142-12_2022_02_03_2022_H5N1
1965232___A_chicken_Netherlands_22001401-001005_2022_01_22_2022_H5N1
1950890___A_mute_swan_Romania_16790_21VIR11355_2021_11_26_2021_H5N1
1922952___A_chicken_Saratov_29-07V_2021_09_30_2021_H5N1
1922920___A_duck_Saratov_29-02V_2021_09_30_2021_H5N1
1938831___A_western_jackdaw_Sweden_SVA211111SZ0376_FB004483_2021_11_08_2021_H5N1
1943101___A_white-tailed_eagle_Sweden_SVA211201SZ0380_FB004721_M-2021_11_26_2021_H5N1
1945580___A_Red_fox_Estonia_TA2126820_21VIR10433-13_2021_11_08_2021_H5N1
1938855___A_greylag_goose__Sweden_SVA211111SZ0376_FB004497_M-2021_11_08_2021_H5N1
1999386___A_white-tailed_eagle_Hokkaido_22-RU-WTE-2_2022_01_02_2022_H5N1
2021943___A_crow_Hokkaido_0103B065_2022_03_29_2022_H5N1
1946370___A_mute_swan_Netherlands_21039824-002_2021_11_25_2021_H5N1
1944235___A_chicken_Italy_21VIR10388_2021_11_30_2021_H5N1
1944331___A_chicken_Italy_21VIR10343_2021_11_28_2021_H5N1
1922888___A_chicken_Tyumen_27-31V_2021_10_06_2021_H5N1
1962116___A_buzzard_Italy_21VIR11899-5_2021_12_27_2021_H5N1
1967020___A_Black-headed_gull_Netherlands_1_2022_01_14_2022_H5N1
1944384___A_turkey_Italy_21VIR10251_2021_11_25_2021_H5N1
1980609___A_chicken_Czech_Republic_25690_2021_12_22_2021_H5N1
1962124___A_owl_Italy_21VIR11057_2021_12_13_2021_H5N1
1980545___A_mute_swan_Czech_Republic_22380_2021_11_15_2021_H5N1
A_Cygnus_columbianus_Hubei_117_2021_H5N1_
A_Cygnus_columbianus_Hubei_126_2021_H5N1_
99
A_Cygnus_columbianus_Hubei_128_2021_H5N1_
A_Cygnus_columbianus_Hubei_127_2021_H5N1_
82
A_Cygnus_columbianus_Hubei_123_2021_H5N1_
78
1997265___A_duck_Bangladesh_19D1818_2021_12_22_2021_H5N1
A_Cygnus_columbianus_Hubei_121_2021_H5N1_
1998405___A_stork_Spain_234-2_22VIR2142-7_2022_01_17_2022_H5N1
1961988___A_turkey_Italy_21VIR11804-1_2021_12_27_2021_H5N1
2021906___A_goose_Czech_Republic_25322-205_2021_12_18_2021_H5N1
1943073___A_chicken_Czech_Republic_23589-3_2021_11_29_2021_H5N1
1980448___A_goose_Czech_Republic_23458-1K_2021_11_26_2021_H5N1
1980577___A_goose_Czech_Republic_22750_2021_11_19_2021_H5N1
1961980___A_turkey_Italy_21VIR11586-2_2021_12_23_2021_H5N1
1923195___A_turkey_Italy_21VIR8585-1_2021_10_18_2021_H5N1
1937608___A_turkey_Poland_H1910-T3_2021_11_01_2021_H5N1
1937624___A_turkey_Poland_H1913-T1_2021_11_02_2021_H5N1
1937664___A_turkey_Poland_H1944-N_2021_11_08_2021_H5N1
1937632___A_turkey_Poland_H1924-T1_2021_11_03_2021_H5N1
1944275___A_chicken_Italy_21VIR10239_2021_11_25_2021_H5N1
1944408___A_turkey_Italy_21VIR9512-1_2021_11_11_2021_H5N1
1999356___A_chicken_Czech_Republic_61-1_2022_12_31_2021_H5N1
1995175___A_chicken_Niger_22VIR1409-13_2022_01_01_2022_H5N1
1866444___A_chicken_Senegal_21VIR1084-3_2021_12_23_2020_H5N1
1896086___A_chicken_Nigeria_VRD21-98_21VIR2288-6_2021_02_12_2021_H5N1
1896067___A_chicken_Nigeria_VRD21-109_21VIR2370-425_2021_03_01_2021_H5N1
1896074___A_chicken_Nigeria_VRD21-37_21VIR2288-2_2021_02_05_2021_H5N1
1958568_A_England_215201407_2021_12_26_2021_H5N1
2042401_A_Colorado_18_2022_04_20_2022_H5N1
1858199___A_chicken_Slovakia_Pah10_21VIR1086-5_2021_01_22_2021_H5N5
1811565___A_buzzard_Germany-MV_AI02166_2020_10_29_2020_H5N5
1848777___A_chicken_Krasnodar_334-01_2021_01_05_2021_H5N8
1841305___A_turkey_Netherlands_21020942-001005_2021_01_04_2021_H5N8
100
1850145___A_red_knot_Germany-SH_AI03419_2020_12_14_2020_H5N3
1813756___A_barnacle_goose_Denmark_14138-1_2020-11-04_H5N8__11_04_2020_H5N8
1920898___A_turkey_Germany-MV_AI03535_2021_03_26_2021_H5N8
1945541___A_greylag_goose_Norway_V294_21VIR7634-1_2021_06_14_2021_H5N8
1638463_A_mallard_Novosibirsk_region_1894k_2019_09_08_2019_H4N6
1942360_A_Anas_platyrhynchos_Belgium_827_2020_01_19_2020_H3N8
1918914_A_mallard_Denmark_12947-12_2020-10-26_H7N5__10_26_2020_H7N5
OK217185_A_Wild-duck_South-Korea_KNU2020-58_2020_09_21_2020_H1N1
1601831_A_duck_Moscow_5586_2018_10_2018__Day_unknown__H1N2
1942925_A_Anas_platyrhynchos_Belgium_204_0003_2020_09_19_2020_H4N6
1440616_A_green_sandpiper_Kurgan_1050_2018_08_26_2018_H3N8
1638399_A_Common_Teal_Amur_region_31b_2019_09_02_2019_H3N6
1775499_A_Anas_platyrhynchos_Belgium_10402_H195386_2017_11_19_2017_H1N1

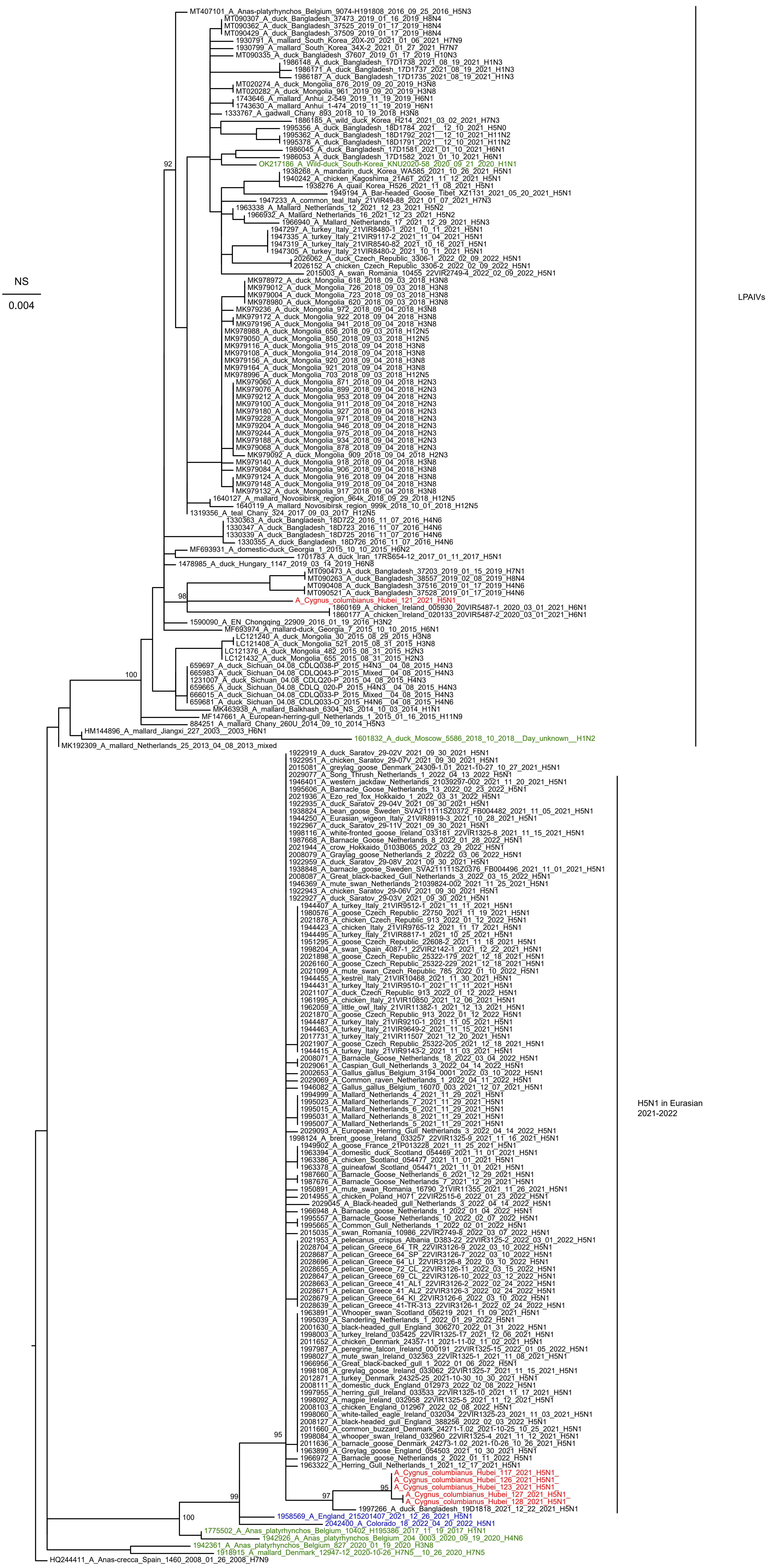

NS
0.004
LPAIVs
H5N1 in Eurasian 2021-2022
92
98
100
95
99
97
100
MT407101_A_Anas-platyrhynchos_Belgium_9074-H191808_2016_09_25_2016_H5N3
MT090307_A_duck_Bangladesh_37473_2019_01_16_2019_H8N4
MT090362_A_duck_Bangladesh_37525_2019_01_17_2019_H8N4
MT090429_A_duck_Bangladesh_37509_2019_01_17_2019_H8N4
1930791_A_mallard_South_Korea_20X-20_2021_01_06_2021_H7N9
1930799_A_mallard_South_Korea_34X-2_2021_01_27_2021_H7N7
MT090335_A_duck_Bangladesh_37607_2019_01_17_2019_H10N3
1986148_A_duck_Bangladesh_17D1738_2021_08_19_2021_H1N3
1986171_A_duck_Bangladesh_17D1737_2021_08_19_2021_H1N3
1986187_A_duck_Bangladesh_17D1735_2021_08_19_2021_H1N3
MT020274_A_duck_Mongolia_876_2019_09_20_2019_H3N8
MT020282_A_duck_Mongolia_961_2019_09_20_2019_H3N8
1743646_A_mallard_Anhui_2-549_2019_11_19_2019_H6N1
1743630_A_mallard_Anhui_1-474_2019_11_19_2019_H6N1
1333767_A_gadwall_Chany_893_2018_10_19_2018_H3N8
1886185_A_wild_duck_Korea_H214_2021_03_02_2021_H7N3
1995356_A_duck_Bangladesh_18D1784_2021__12_10_2021_H5N0
1995362_A_duck_Bangladesh_18D1792_2021__12_10_2021_H11N2
1995378_A_duck_Bangladesh_18D1791_2021__12_10_2021_H11N2
1986045_A_duck_Bangladesh_17D1581_2021_01_10_2021_H6N1
1986053_A_duck_Bangladesh_17D1582_2021_01_10_2021_H6N1
OK217186_A_Wild-duck_South-Korea_KNU2020-58_2020_09_21_2020_H1N1
1938268_A_mandarin_duck_Korea_WA585_2021_10_26_2021_H5N1
1940242_A_chicken_Kagoshima_21A6T_2021_11_12_2021_H5N1
1938276_A_quail_Korea_H526_2021_11_08_2021_H5N1
1949194_A_Bar-headed_Goose_Tibet_XZ1131_2021_05_20_2021_H5N1
1947233_A_common_teal_Italy_21VIR49-88_2021_01_07_2021_H7N3
1963338_A_Mallard_Netherlands_12_2021_12_23_2021_H5N2
1966932_A_Mallard_Netherlands_16_2021_12_23_2021_H5N2
1966940_A_Mallard_Netherlands_17_2021_12_29_2021_H5N3
1947297_A_turkey_Italy_21VIR8480-1_2021_10_11_2021_H5N1
1947335_A_turkey_Italy_21VIR9117-2_2021_11_04_2021_H5N1
1947319_A_turkey_Italy_21VIR8540-82_2021_10_16_2021_H5N1
1947305_A_turkey_Italy_21VIR8480-2_2021_10_11_2021_H5N1
2026062_A_duck_Czech_Republic_3306-1_2022_02_09_2022_H5N1
2026152_A_chicken_Czech_Republic_3306-2_2022_02_09_2022_H5N1
2015003_A_swan_Romania_10455_22VIR2749-4_2022_02_09_2022_H5N1
MK978972_A_duck_Mongolia_618_2018_09_03_2018_H3N8
MK979012_A_duck_Mongolia_726_2018_09_03_2018_H3N8
MK979004_A_duck_Mongolia_723_2018_09_03_2018_H3N8
MK978980_A_duck_Mongolia_620_2018_09_03_2018_H3N8
MK979236_A_duck_Mongolia_972_2018_09_04_2018_H3N8
MK979172_A_duck_Mongolia_922_2018_09_04_2018_H3N8
MK979196_A_duck_Mongolia_941_2018_09_04_2018_H3N8
MK978988_A_duck_Mongolia_656_2018_09_03_2018_H12N5
MK979050_A_duck_Mongolia_850_2018_09_03_2018_H12N5
MK979116_A_duck_Mongolia_915_2018_09_04_2018_H3N8
MK979108_A_duck_Mongolia_914_2018_09_04_2018_H3N8
MK979156_A_duck_Mongolia_920_2018_09_04_2018_H3N8
MK979164_A_duck_Mongolia_921_2018_09_04_2018_H3N8
MK978996_A_duck_Mongolia_703_2018_09_03_2018_H12N5
MK979060_A_duck_Mongolia_871_2018_09_04_2018_H2N3
MK979076_A_duck_Mongolia_899_2018_09_04_2018_H2N3
MK979212_A_duck_Mongolia_953_2018_09_04_2018_H2N3
MK979100_A_duck_Mongolia_911_2018_09_04_2018_H2N3
MK979180_A_duck_Mongolia_927_2018_09_04_2018_H2N3
MK979228_A_duck_Mongolia_971_2018_09_04_2018_H2N3
MK979204_A_duck_Mongolia_946_2018_09_04_2018_H2N3
MK979244_A_duck_Mongolia_975_2018_09_04_2018_H2N3
MK979188_A_duck_Mongolia_934_2018_09_04_2018_H2N3
MK979068_A_duck_Mongolia_878_2018_09_04_2018_H2N3
MK979092_A_duck_Mongolia_909_2018_09_04_2018_H2N3
MK979140_A_duck_Mongolia_918_2018_09_04_2018_H3N8
MK979084_A_duck_Mongolia_906_2018_09_04_2018_H3N8
MK979124_A_duck_Mongolia_916_2018_09_04_2018_H3N8
MK979148_A_duck_Mongolia_919_2018_09_04_2018_H3N8
MK979132_A_duck_Mongolia_917_2018_09_04_2018_H3N8
1640127_A_mallard_Novosibirsk_region_964k_2018_09_29_2018_H12N5
1640119_A_mallard_Novosibirsk_region_999k_2018_10_01_2018_H12N5
1319356_A_teal_Chany_324_2017_09_03_2017_H12N5
1330363_A_duck_Bangladesh_18D722_2016_11_07_2016_H4N6
1330347_A_duck_Bangladesh_18D723_2016_11_07_2016_H4N6
1330339_A_duck_Bangladesh_18D725_2016_11_07_2016_H4N6
1330355_A_duck_Bangladesh_18D726_2016_11_07_2016_H4N6
MF693931_A_domestic-duck_Georgia_1_2015_10_10_2015_H6N2
1701783_A_duck_Iran_17RS654-12_2017_01_11_2017_H5N1
1478985_A_duck_Hungary_1147_2019_03_14_2019_H6N8
MT090473_A_duck_Bangladesh_37203_2019_01_15_2019_H7N1
MT090263_A_duck_Bangladesh_38557_2019_02_08_2019_H8N4
MT090408_A_duck_Bangladesh_37516_2019_01_17_2019_H4N6
MT090521_A_duck_Bangladesh_37528_2019_01_17_2019_H4N6
A_Cygnus_columbianus_Hubei_121_2021_H5N1_
1860169_A_chicken_Ireland_005930_20VIR5487-1_2020_03_01_2021_H6N1
1860177_A_chicken_Ireland_020133_20VIR5487-2_2020_03_01_2021_H6N1
1590090_A_EN_Chongqing_22909_2016_01_19_2016_H3N2
MF693974_A_mallard-duck_Georgia_7_2015_10_10_2015_H6N1
LC121240_A_duck_Mongolia_30_2015_08_29_2015_H3N8
LC121408_A_duck_Mongolia_521_2015_08_31_2015_H3N8
LC121376_A_duck_Mongolia_482_2015_08_31_2015_H2N3
LC121432_A_duck_Mongolia_655_2015_08_31_2015_H2N3
659697_A_duck_Sichuan_04.08_CDLQ038-P_2015_H4N3__04_08_2015_H4N3
665983_A_duck_Sichuan_04.08_CDLQ043-P_2015_Mixed__04_08_2015_H4N3
1231007_A_duck_Sichuan_04.08_CDLQ20-P_2015_04_08_2015_H4N3
659665_A_duck_Sichuan_04.08_CDLQ_020-P_2015_H4N3__04_08_2015_H4N3
666015_A_duck_Sichuan_04.08_CDLQ033-P_2015_Mixed__04_08_2015_H4N3
659681_A_duck_Sichuan_04.08_CDLQ033-O_2015_H4N6__04_08_2015_H4N6
MK463938_A_mallard_Balkhash_6304_NS_2014_10_03_2014_H1N1
MF147661_A_European-herring-gull_Netherlands_1_2015_01_16_2015_H11N9
884251_A_mallard_Chany_260U_2014_09_10_2014_H5N3
HM144896_A_mallard_Jiangxi_227_2003__2003_H6N1
1601832_A_duck_Moscow_5586_2018_10_2018__Day_unknown__H1N2
MK192309_A_mallard_Netherlands_25_2013_04_08_2013_mixed
1922919_A_duck_Saratov_29-02V_2021_09_30_2021_H5N1
1922951_A_chicken_Saratov_29-07V_2021_09_30_2021_H5N1
2015081_A_greylag_goose_Denmark_24309-1.01_2021-10-27_10_27_2021_H5N1
2029077_A_Song_Thrush_Netherlands_1_2022_04_13_2022_H5N1
1946401_A_western_jackdaw_Netherlands_21039297-002_2021_11_20_2021_H5N1
1995606_A_Barnacle_Goose_Netherlands_13_2022_02_23_2022_H5N1
2021936_A_Ezo_red_fox_Hokkaido_1_2022_03_31_2022_H5N1
1922935_A_duck_Saratov_29-04V_2021_09_30_2021_H5N1
1938824_A_bean_goose_Sweden_SVA211111SZ0372_FB004482_2021_11_05_2021_H5N1
1944250_A_Eurasian_wigeon_Italy_21VIR8919-3_2021_10_28_2021_H5N1
1922967_A_duck_Saratov_29-11V_2021_09_30_2021_H5N1
1998116_A_white-fronted_goose_Ireland_033181_22VIR1325-8_2021_11_15_2021_H5N1
1987668_A_Barnacle_Goose_Netherlands_8_2022_01_28_2022_H5N1
2021944_A_crow_Hokkaido_0103B065_2022_03_29_2022_H5N1
2008079_A_Graylag_goose_Netherlands_2_20222_03_06_2022_H5N1
1922959_A_duck_Saratov_29-08V_2021_09_30_2021_H5N1
1938848_A_barnacle_goose_Sweden_SVA211111SZ0376_FB004496_2021_11_01_2021_H5N1
2008087_A_Great_black-backed_Gull_Netherlands_3_2022_03_15_2022_H5N1
1946369_A_mute_swan_Netherlands_21039824-002_2021_11_25_2021_H5N1
1922943_A_chicken_Saratov_29-06V_2021_09_30_2021_H5N1
1922927_A_duck_Saratov_29-03V_2021_09_30_2021_H5N1
1944407_A_turkey_Italy_21VIR9512-1_2021_11_11_2021_H5N1
1980576_A_goose_Czech_Republic_22750_2021_11_19_2021_H5N1
2021878_A_chicken_Czech_Republic_913_2022_01_12_2022_H5N1
1944423_A_chicken_Italy_21VIR9765-12_2021_11_17_2021_H5N1
1944495_A_turkey_Italy_21VIR8817-1_2021_10_25_2021_H5N1
1951295_A_goose_Czech_Republic_22608-2_2021_11_18_2021_H5N1
1998204_A_swan_Spain_4087-1_22VIR2142-1_2021_12_22_2021_H5N1
2021898_A_goose_Czech_Republic_25322-179_2021_12_18_2021_H5N1
2026160_A_goose_Czech_Republic_25322-229_2021_12_18_2021_H5N1
2021099_A_mute_swan_Czech_Republic_785_2022_01_10_2022_H5N1
1944455_A_kestrel_Italy_21VIR10468_2021_11_30_2021_H5N1
1944431_A_turkey_Italy_21VIR9510-1_2021_11_11_2021_H5N1
2021107_A_duck_Czech_Republic_913_2022_01_12_2022_H5N1
1961995_A_chicken_Italy_21VIR10850_2021_12_06_2021_H5N1
1962059_A_little_owl_Italy_21VIR11382-1_2021_12_13_2021_H5N1
2021870_A_goose_Czech_Republic_913_2022_01_12_2022_H5N1
1944487_A_turkey_Italy_21VIR9210-1_2021_11_05_2021_H5N1
1944463_A_turkey_Italy_21VIR9649-2_2021_11_15_2021_H5N1
2017731_A_turkey_Italy_21VIR11507_2021_12_20_2021_H5N1
2021907_A_goose_Czech_Republic_25322-205_2021_12_18_2021_H5N1
1944415_A_turkey_Italy_21VIR9143-2_2021_11_03_2021_H5N1
2008071_A_Barnacle_Goose_Netherlands_18_2022_03_04_2022_H5N1
2029061_A_Caspian_Gull_Netherlands_3_2022_04_14_2022_H5N1
2002653_A_Gallus_gallus_Belgium_3194_0001_2022_03_10_2022_H5N1
2029069_A_Common_raven_Netherlands_1_2022_04_11_2022_H5N1
1946082_A_Gallus_gallus_Belgium_16070_003_2021_12_07_2021_H5N1
1994999_A_Mallard_Netherlands_4_2021_11_29_2021_H5N1
1995023_A_Mallard_Netherlands_7_2021_11_29_2021_H5N1
1995015_A_Mallard_Netherlands_6_2021_11_29_2021_H5N1
1995031_A_Mallard_Netherlands_8_2021_11_29_2021_H5N1
1995007_A_Mallard_Netherlands_5_2021_11_29_2021_H5N1
2029093_A_European_Herring_Gull_Netherlands_3_2022_04_14_2022_H5N1
1998124_A_brent_goose_Ireland_033257_22VIR1325-9_2021_11_16_2021_H5N1
1949902_A_goose_France_21P013228_2021_11_25_2021_H5N1
1963394_A_domestic_duck_Scotland_054469_2021_11_01_2021_H5N1
1963386_A_chicken_Scotland_054477_2021_11_01_2021_H5N1
1963378_A_guineafowl_Scotland_054471_2021_11_01_2021_H5N1
1987660_A_Barnacle_Goose_Netherlands_6_2021_12_29_2021_H5N1
1987676_A_Barnacle_Goose_Netherlands_7_2021_12_29_2021_H5N1
1950891_A_mute_swan_Romania_16790_21VIR11355_2021_11_26_2021_H5N1
2014955_A_chicken_Poland_H071_22VIR2515-6_2022_01_23_2022_H5N1
2029045_A_Black-headed_gull_Netherlands_3_2022_04_14_2022_H5N1
1966948_A_Barnacle_goose_Netherlands_1_2022_01_04_2022_H5N1
1995557_A_Barnacle_Goose_Netherlands_10_2022_02_07_2022_H5N1
1995665_A_Common_Gull_Netherlands_1_2022_02_01_2022_H5N1
2015035_A_swan_Romania_10986_22VIR2749-8_2022_03_07_2022_H5N1
2021953_A_pelecanus_crispus_Albania_D383-22_22VIR3125-2_2022_03_01_2022_H5N1
2028704_A_pelican_Greece_64_TR_22VIR3126-9_2022_03_10_2022_H5N1
2028687_A_pelican_Greece_64_SP_22VIR3126-7_2022_03_10_2022_H5N1
2028696_A_pelican_Greece_64_LI_22VIR3126-8_2022_03_10_2022_H5N1
2028655_A_pelican_Greece_72_CL_22VIR3126-11_2022_03_15_2022_H5N1
2028647_A_pelican_Greece_69_CL_22VIR3126-10_2022_03_12_2022_H5N1
2028663_A_pelican_Greece_41_AL1_22VIR3126-2_2022_02_24_2022_H5N1
2028671_A_pelican_Greece_41_AL2_22VIR3126-3_2022_02_24_2022_H5N1
2028679_A_pelican_Greece_64_KI_22VIR3126-6_2022_03_10_2022_H5N1
2028639_A_pelican_Greece_41-TR-313_22VIR3126-1_2022_02_24_2022_H5N1
1963891_A_Whooper_swan_Scotland_056219_2021_11_09_2021_H5N1
1995039_A_Sanderling_Netherlands_1_2022_01_29_2022_H5N1
2001630_A_black-headed_gull_England_306270_2022_01_31_2022_H5N1
1998003_A_turkey_Ireland_035425_22VIR1325-17_2021_12_06_2021_H5N1
2011652_A_chicken_Denmark_24357-11_2021-11-02_11_02_2021_H5N1
1997987_A_peregrine_falcon_Ireland_000191_22VIR1325-15_2022_01_05_2022_H5N1
1998027_A_mute_swan_Ireland_032363_22VIR1325-1_2021_11_08_2021_H5N1
1966956_A_Great_black-backed_gull_1_2022_01_06_2022_H5N1
1998108_A_greylag_goose_Ireland_033062_22VIR1325-7_2021_11_15_2021_H5N1
2012871_A_turkey_Denmark_24325-25_2021-10-30_10_30_2021_H5N1
2008111_A_domestic_duck_England_012973_2022_02_08_2022_H5N1
1997955_A_herring_gull_Ireland_033533_22VIR1325-10_2021_11_17_2021_H5N1
1998092_A_magpie_Ireland_032958_22VIR1325-5_2021_11_12_2021_H5N1
2008103_A_chicken_England_012967_2022_02_08_2022_H5N1
1998060_A_white-tailed_eagle_Ireland_032034_22VIR1325-23_2021_11_03_2021_H5N1
2008127_A_black-headed_gull_England_388256_2022_02_03_2022_H5N1
2011660_A_common_buzzard_Denmark_24271-1.02_2021-10-25_10_25_2021_H5N1
1998084_A_whooper_swan_Ireland_032960_22VIR1325-4_2021_11_12_2021_H5N1
2011636_A_barnacle_goose_Denmark_24273-1.02_2021-10-26_10_26_2021_H5N1
1963899_A_Greylag_goose_England_054503_2021_10_30_2021_H5N1
1966972_A_Barnacle_goose_Netherlands_2_2022_01_11_2022_H5N1
1963322_A_Herring_Gull_Netherlands_1_2021_12_17_2021_H5N1
A_Cygnus_columbianus_Hubei_117_2021_H5N1_
A_Cygnus_columbianus_Hubei_126_2021_H5N1_
A_Cygnus_columbianus_Hubei_123_2021_H5N1_
A_Cygnus_columbianus_Hubei_127_2021_H5N1_
A_Cygnus_columbianus_Hubei_128_2021_H5N1_
1997266_A_duck_Bangladesh_19D1818_2021_12_22_2021_H5N1
1958569_A_England_215201407_2021_12_26_2021_H5N1
2042400_A_Colorado_18_2022_04_20_2022_H5N1
1775502_A_Anas_platyrhynchos_Belgium_10402_H195386_2017_11_19_2017_H1N1
1942926_A_Anas_platyrhynchos_Belgium_204_0003_2020_09_19_2020_H4N6
1942361_A_Anas_platyrhynchos_Belgium_827_2020_01_19_2020_H3N8
1918915_A_mallard_Denmark_12947-12_2020-10-26_H7N5__10_26_2020_H7N5
HQ244411_A_Anas-crecca_Spain_1460_2008_01_26_2008_H7N9

Fig S2. Molecular dating analysis of six H5N1 viruses. Maximum clade credibility trees of each gene segment were established from Bayesian Evolutionary Analysis using Sampling Trees (BEAST) v1.10.4 under the substitution models selected by the ModelFinder function of IQ-tree according to the value of AIC, with an uncorrelated relaxed clock and chain length of 100,000,000. Furthermore, Tracer v1.7 was used to confirm the reliability of the results. The trees were summarized by Tree Annotator with 10% burn-in cutoffs. Sequences reported in this study are marked in red. 95% highest posterior density (HPD) of age estimates of nodes are shown as horizontal bars.

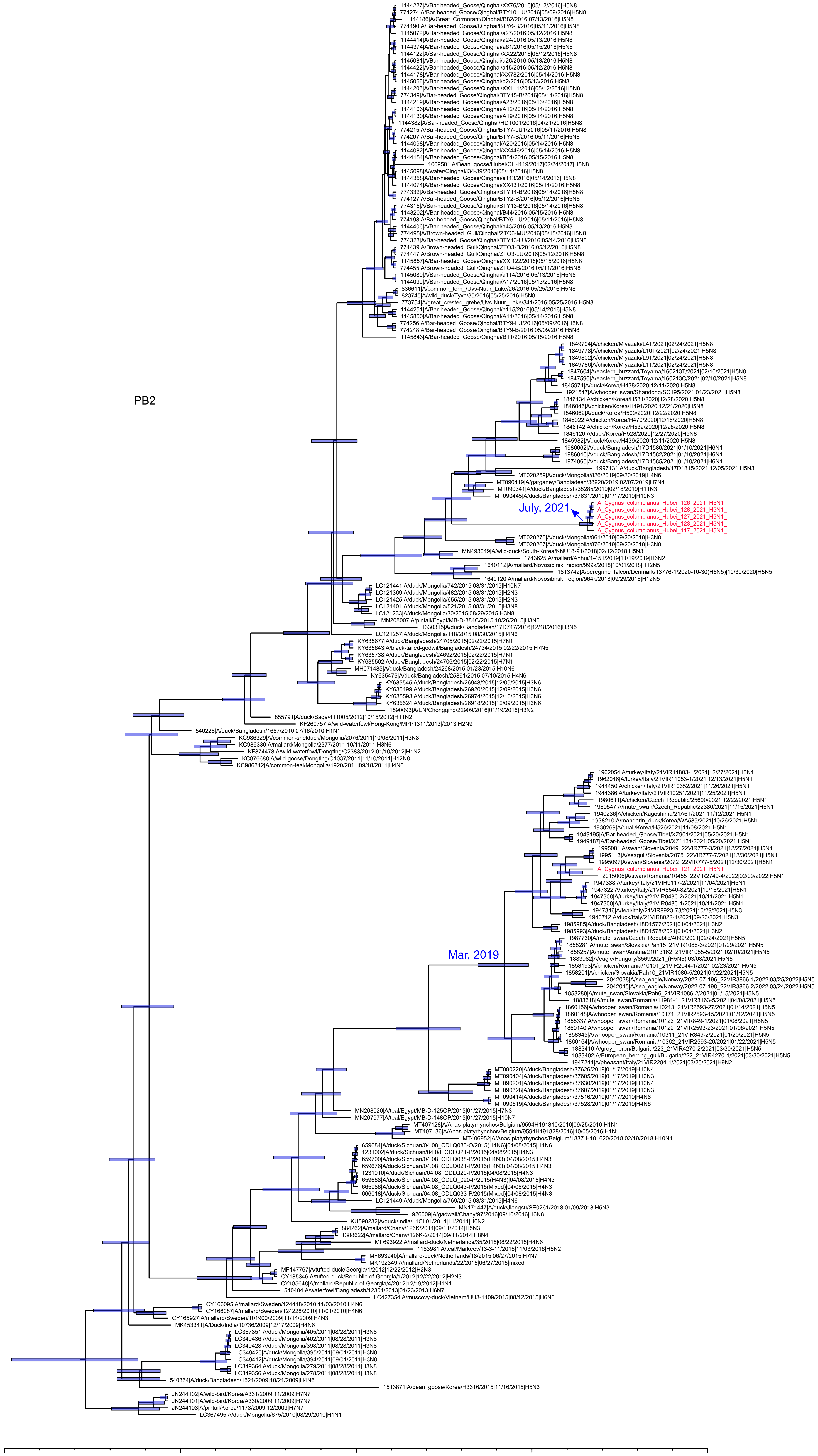
PB2
July, 2021
Mar, 2019
2005
2010
2015
2020
2025

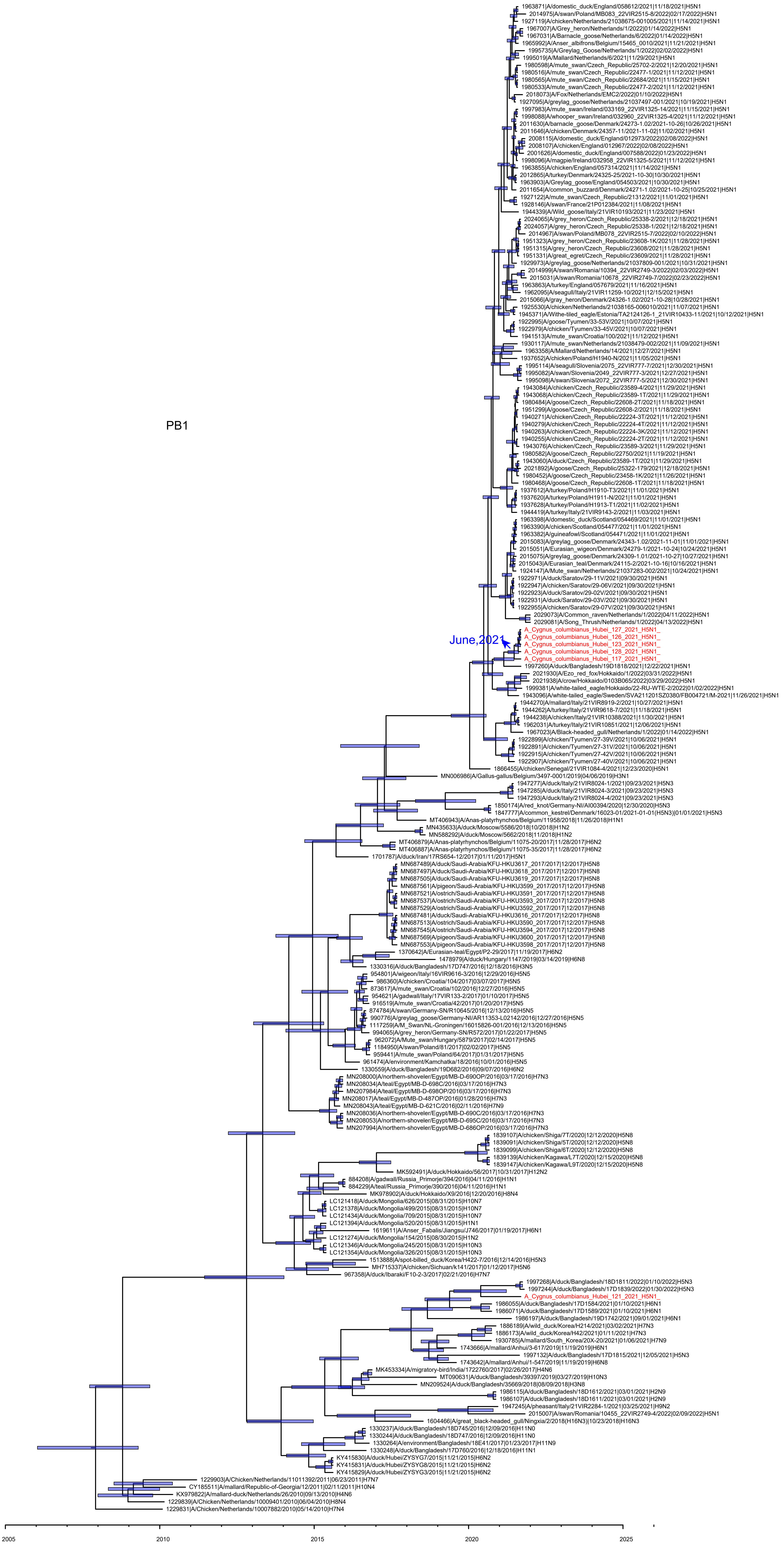
PB1
June,2021
A_Cygnus_columbianus_Hubei_127_2021_H5N1_
A_Cygnus_columbianus_Hubei_126_2021_H5N1_
A_Cygnus_columbianus_Hubei_123_2021_H5N1_
A_Cygnus_columbianus_Hubei_128_2021_H5N1_
A_Cygnus_columbianus_Hubei_117_2021_H5N1_
A_Cygnus_columbianus_Hubei_121_2021_H5N1_
2005
2010
2015
2020
2025

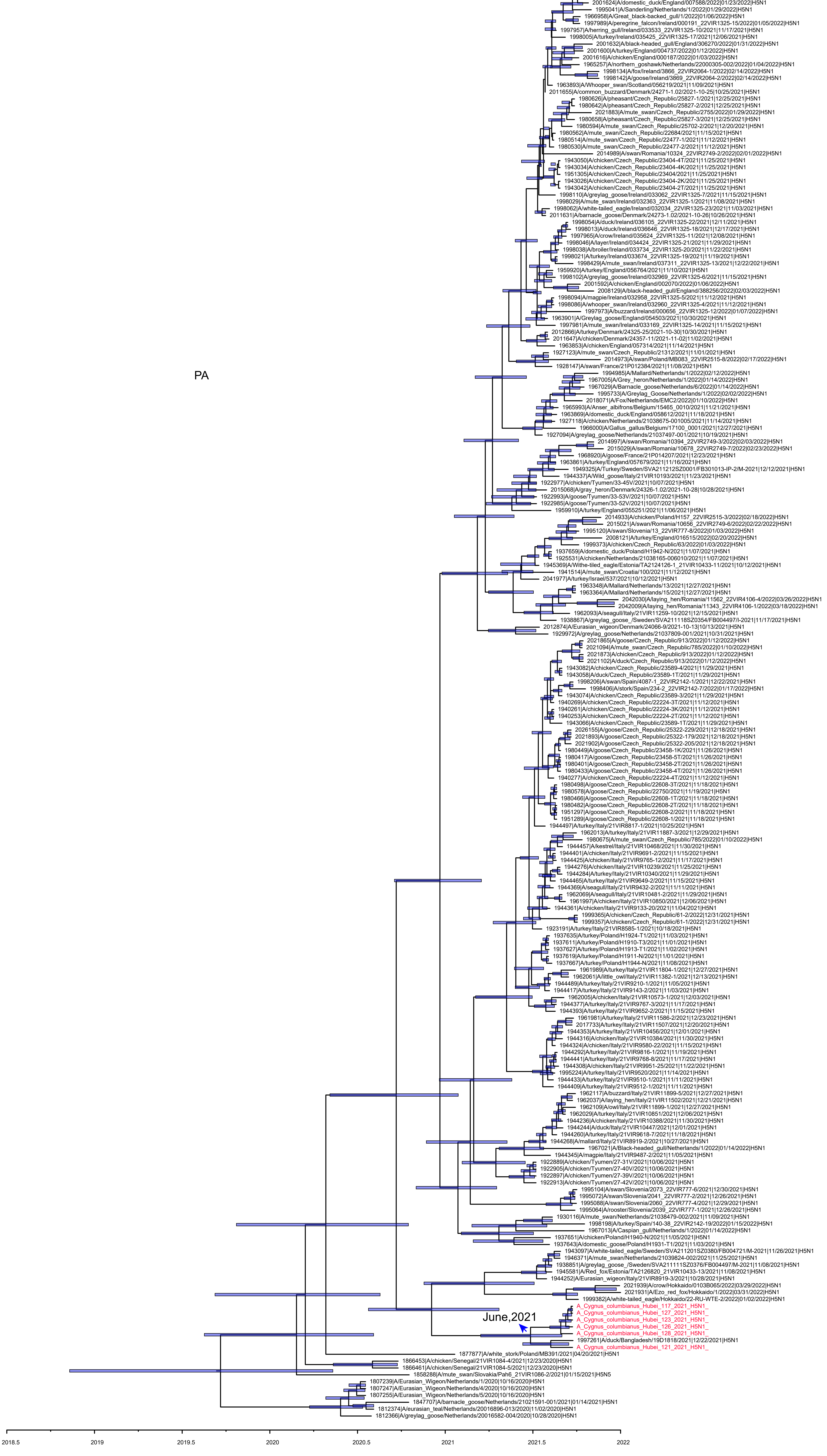
PA
June,2021
2018.5
2019
2019.5
2020
2020.5
2021
2021.5
2022

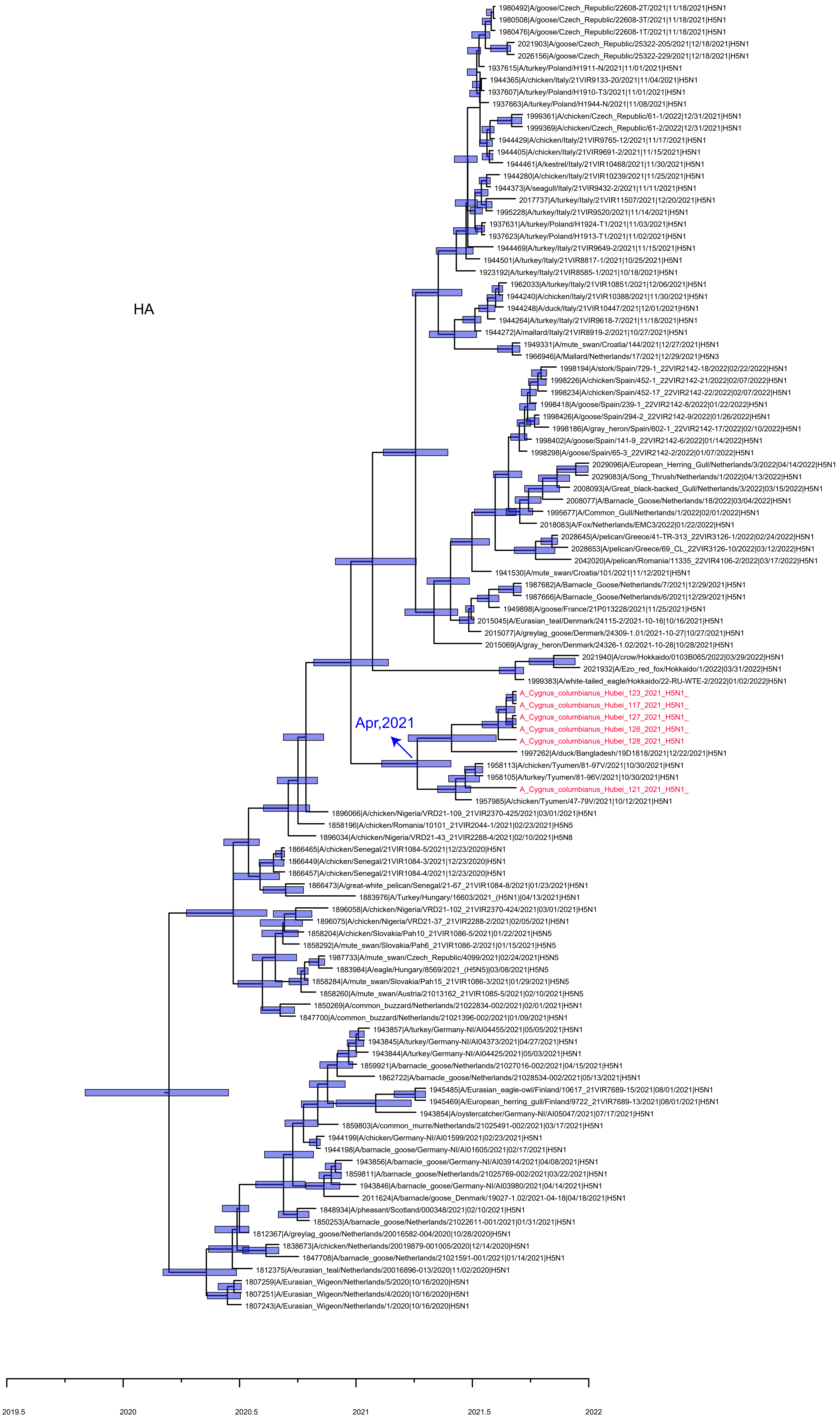

HA
1980492|A/goose/Czech_Republic/22608-2T/2021|11/18/2021|H5N1
1980508|A/goose/Czech_Republic/22608-3T/2021|11/18/2021|H5N1
1980476|A/goose/Czech_Republic/22608-1T/2021|11/18/2021|H5N1
2021903|A/goose/Czech_Republic/25322-205/2021|12/18/2021|H5N1
2026156|A/goose/Czech_Republic/25322-229/2021|12/18/2021|H5N1
1937615|A/turkey/Poland/H1911-N/2021|11/01/2021|H5N1
1944365|A/chicken/Italy/21VIR9133-20/2021|11/04/2021|H5N1
1937607|A/turkey/Poland/H1910-T3/2021|11/01/2021|H5N1
1937663|A/turkey/Poland/H1944-N/2021|11/08/2021|H5N1
1999361|A/chicken/Czech_Republic/61-1/2022|12/31/2021|H5N1
1999369|A/chicken/Czech_Republic/61-2/2022|12/31/2021|H5N1
1944429|A/chicken/Italy/21VIR9765-12/2021|11/17/2021|H5N1
1944405|A/chicken/Italy/21VIR9691-2/2021|11/15/2021|H5N1
1944461|A/kestrel/Italy/21VIR10468/2021|11/30/2021|H5N1
1944280|A/chicken/Italy/21VIR10239/2021|11/25/2021|H5N1
1944373|A/seagull/Italy/21VIR9432-2/2021|11/11/2021|H5N1
2017737|A/turkey/Italy/21VIR11507/2021|12/20/2021|H5N1
1995228|A/turkey/Italy/21VIR9520/2021|11/14/2021|H5N1
1937631|A/turkey/Poland/H1924-T1/2021|11/03/2021|H5N1
1937623|A/turkey/Poland/H1913-T1/2021|11/02/2021|H5N1
1944469|A/turkey/Italy/21VIR9649-2/2021|11/15/2021|H5N1
1944501|A/turkey/Italy/21VIR8817-1/2021|10/25/2021|H5N1
1923192|A/turkey/Italy/21VIR8585-1/2021|10/18/2021|H5N1
1962033|A/turkey/Italy/21VIR10851/2021|12/06/2021|H5N1
1944240|A/chicken/Italy/21VIR10388/2021|11/30/2021|H5N1
1944248|A/duck/Italy/21VIR10447/2021|12/01/2021|H5N1
1944264|A/turkey/Italy/21VIR9618-7/2021|11/18/2021|H5N1
1944272|A/mallard/Italy/21VIR8919-2/2021|10/27/2021|H5N1
1949331|A/mute_swan/Croatia/144/2021|12/27/2021|H5N1
1966946|A/Mallard/Netherlands/17/2021|12/29/2021|H5N3
1998194|A/stork/Spain/729-1_22VIR2142-18/2022|02/22/2022|H5N1
1998226|A/chicken/Spain/452-1_22VIR2142-21/2022|02/07/2022|H5N1
1998234|A/chicken/Spain/452-17_22VIR2142-22/2022|02/07/2022|H5N1
1998418|A/goose/Spain/239-1_22VIR2142-8/2022|01/22/2022|H5N1
1998426|A/goose/Spain/294-2_22VIR2142-9/2022|01/26/2022|H5N1
1998186|A/gray_heron/Spain/602-1_22VIR2142-17/2022|02/10/2022|H5N1
1998402|A/goose/Spain/141-9_22VIR2142-6/2022|01/14/2022|H5N1
1998298|A/goose/Spain/65-3_22VIR2142-2/2022|01/07/2022|H5N1
2029096|A/European_Herring_Gull/Netherlands/3/2022|04/14/2022|H5N1
2029083|A/Song_Thrush/Netherlands/1/2022|04/13/2022|H5N1
2008093|A/Great_black-backed_Gull/Netherlands/3/2022|03/15/2022|H5N1
2008077|A/Barnacle_Goose/Netherlands/18/2022|03/04/2022|H5N1
1995677|A/Common_Gull/Netherlands/1/2022|02/01/2022|H5N1
2018083|A/Fox/Netherlands/EMC3/2022|01/22/2022|H5N1
2028645|A/pelican/Greece/41-TR-313_22VIR3126-1/2022|02/24/2022|H5N1
2028653|A/pelican/Greece/69_CL_22VIR3126-10/2022|03/12/2022|H5N1
2042020|A/pelican/Romania/11335_22VIR4106-2/2022|03/17/2022|H5N1
1941530|A/mute_swan/Croatia/101/2021|11/12/2021|H5N1
1987682|A/Barnacle_Goose/Netherlands/7/2021|12/29/2021|H5N1
1987666|A/Barnacle_Goose/Netherlands/6/2021|12/29/2021|H5N1
1949898|A/goose/France/21P013228/2021|11/25/2021|H5N1
2015045|A/Eurasian_teal/Denmark/24115-2/2021-10-16|10/16/2021|H5N1
2015077|A/greylag_goose/Denmark/24309-1.01/2021-10-27|10/27/2021|H5N1
2015069|A/gray_heron/Denmark/24326-1.02/2021-10-28|10/28/2021|H5N1
2021940|A/crow/Hokkaido/0103B065/2022|03/29/2022|H5N1
2021932|A/Ezo_red_fox/Hokkaido/1/2022|03/31/2022|H5N1
1999383|A/white-tailed_eagle/Hokkaido/22-RU-WTE-2/2022|01/02/2022|H5N1
A_Cygnus_columbianus_Hubei_123_2021_H5N1_
A_Cygnus_columbianus_Hubei_117_2021_H5N1_
A_Cygnus_columbianus_Hubei_127_2021_H5N1_
A_Cygnus_columbianus_Hubei_126_2021_H5N1_
A_Cygnus_columbianus_Hubei_128_2021_H5N1
Apr,2021
1997262|A/duck/Bangladesh/19D1818/2021|12/22/2021|H5N1
1958113|A/chicken/Tyumen/81-97V/2021|10/30/2021|H5N1
1958105|A/turkey/Tyumen/81-96V/2021|10/30/2021|H5N1
A_Cygnus_columbianus_Hubei_121_2021_H5N1_
1957985|A/chicken/Tyumen/47-79V/2021|10/12/2021|H5N1
1896066|A/chicken/Nigeria/VRD21-109_21VIR2370-425/2021|03/01/2021|H5N1
1858196|A/chicken/Romania/10101_21VIR2044-1/2021|02/23/2021|H5N5
1896034|A/chicken/Nigeria/VRD21-43_21VIR2288-4/2021|02/10/2021|H5N8
1866465|A/chicken/Senegal/21VIR1084-5/2021|12/23/2020|H5N1
1866449|A/chicken/Senegal/21VIR1084-3/2021|12/23/2020|H5N1
1866457|A/chicken/Senegal/21VIR1084-4/2021|12/23/2020|H5N1
1866473|A/great-white_pelican/Senegal/21-67_21VIR1084-8/2021|01/23/2021|H5N1
1883976|A/Turkey/Hungary/16603/2021_(H5N1)|04/13/2021|H5N1
1896058|A/chicken/Nigeria/VRD21-102_21VIR2370-424/2021|03/01/2021|H5N1
1896075|A/chicken/Nigeria/VRD21-37_21VIR2288-2/2021|02/05/2021|H5N1
1858204|A/chicken/Slovakia/Pah10_21VIR1086-5/2021|01/22/2021|H5N5
1858292|A/mute_swan/Slovakia/Pah6_21VIR1086-2/2021|01/15/2021|H5N5
1987733|A/mute_swan/Czech_Republic/4099/2021|02/24/2021|H5N5
1883984|A/eagle/Hungary/8569/2021_(H5N5)|03/08/2021|H5N5
1858284|A/mute_swan/Slovakia/Pah15_21VIR1086-3/2021|01/29/2021|H5N5
1858260|A/mute_swan/Austria/21013162_21VIR1085-5/2021|02/10/2021|H5N5
1850269|A/common_buzzard/Netherlands/21022834-002/2021|02/01/2021|H5N1
1847700|A/common_buzzard/Netherlands/21021396-002/2021|01/09/2021|H5N1
1943857|A/turkey/Germany-NI/AI04455/2021|05/05/2021|H5N1
1943845|A/turkey/Germany-NI/AI04373/2021|04/27/2021|H5N1
1943844|A/turkey/Germany-NI/AI04425/2021|05/03/2021|H5N1
1859921|A/barnacle_goose/Netherlands/21027016-002/2021|04/15/2021|H5N1
1862722|A/barnacle_goose/Netherlands/21028534-002/2021|05/13/2021|H5N1
1945485|A/Eurasian_eagle-owl/Finland/10617_21VIR7689-15/2021|08/01/2021|H5N1
1945469|A/European_herring_gull/Finland/9722_21VIR7689-13/2021|08/01/2021|H5N1
1943854|A/oystercatcher/Germany-NI/AI05047/2021|07/17/2021|H5N1
1859803|A/common_murre/Netherlands/21025491-002/2021|03/17/2021|H5N1
1944199|A/chicken/Germany-NI/AI01599/2021|02/23/2021|H5N1
1944198|A/barnacle_goose/Germany-NI/AI01605/2021|02/17/2021|H5N1
1943856|A/barnacle_goose/Germany-NI/AI03914/2021|04/08/2021|H5N1
1859811|A/barnacle_goose/Netherlands/21025769-002/2021|03/22/2021|H5N1
1943846|A/barnacle_goose/Germany-NI/AI03980/2021|04/14/2021|H5N1
2011624|A/barnacle/goose_Denmark/19027-1.02/2021-04-18|04/18/2021|H5N1
1848934|A/pheasant/Scotland/000348/2021|02/10/2021|H5N1
1850253|A/barnacle_goose/Netherlands/21022611-001/2021|01/31/2021|H5N1
1812367|A/greylag_goose/Netherlands/20016582-004/2020|10/28/2020|H5N1
1838673|A/chicken/Netherlands/20019879-001005/2020|12/14/2020|H5N1
1847708|A/barnacle_goose/Netherlands/21021591-001/2021|01/14/2021|H5N1
1812375|A/eurasian_teal/Netherlands/20016896-013/2020|11/02/2020|H5N1
1807259|A/Eurasian_Wigeon/Netherlands/5/2020|10/16/2020|H5N1
1807251|A/Eurasian_Wigeon/Netherlands/4/2020|10/16/2020|H5N1
1807243|A/Eurasian_Wigeon/Netherlands/1/2020|10/16/2020|H5N1
2019.5
2020
2020.5
2021
2021.5
2022

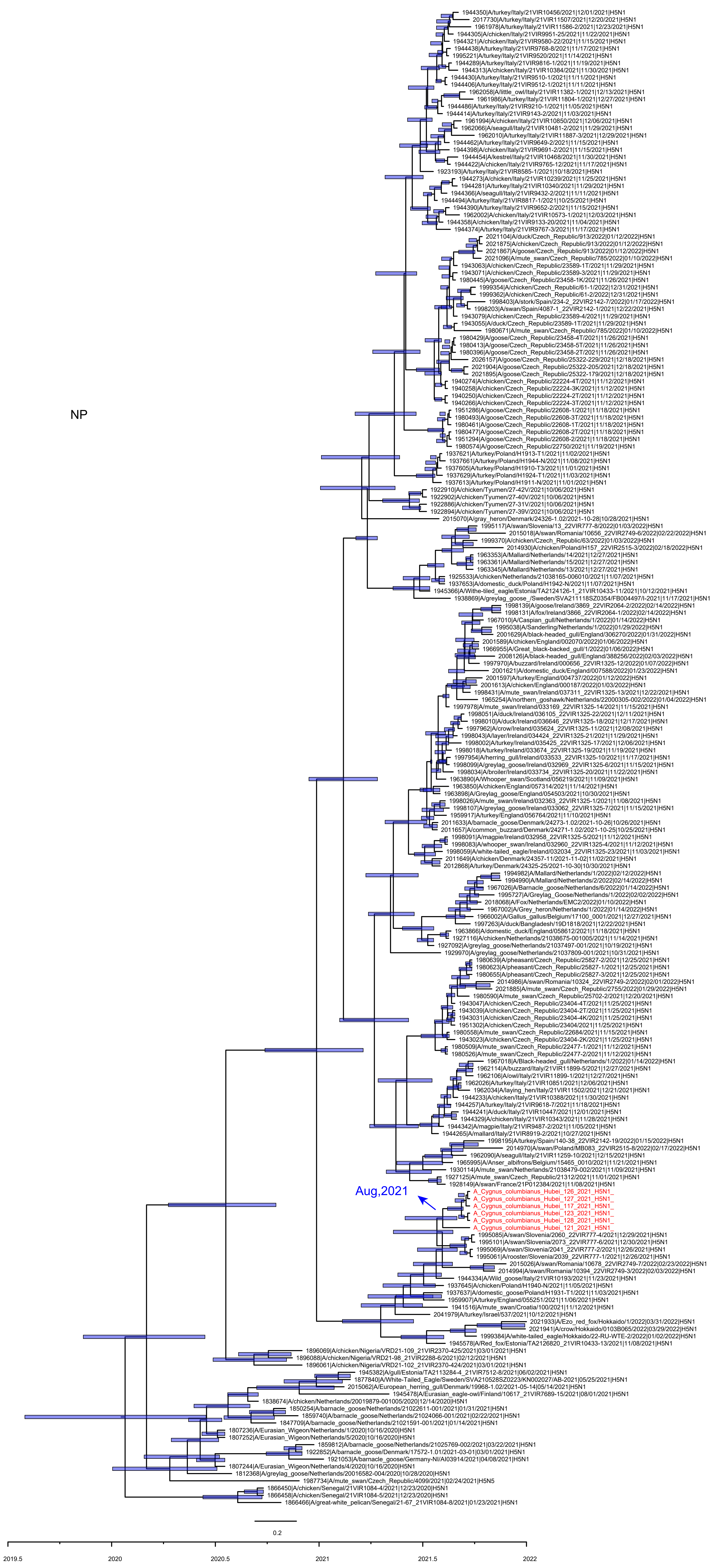
NP
1944350|A/turkey/Italy/21VIR10456/2021|12/01/2021|H5N1
2017730|A/turkey/Italy/21VIR11507/2021|12/20/2021|H5N1
1961978|A/turkey/Italy/21VIR11586-2/2021|12/23/2021|H5N1
1944305|A/chicken/Italy/21VIR9951-25/2021|11/22/2021|H5N1
1944321|A/chicken/Italy/21VIR9580-22/2021|11/15/2021|H5N1
1944438|A/turkey/Italy/21VIR9768-8/2021|11/17/2021|H5N1
1995221|A/turkey/Italy/21VIR9520/2021|11/14/2021|H5N1
1944289|A/turkey/Italy/21VIR9816-1/2021|11/19/2021|H5N1
1944313|A/chicken/Italy/21VIR10384/2021|11/30/2021|H5N1
1944430|A/turkey/Italy/21VIR9510-1/2021|11/11/2021|H5N1
1944406|A/turkey/Italy/21VIR9512-1/2021|11/11/2021|H5N1
1962058|A/little_owl/Italy/21VIR11382-1/2021|12/13/2021|H5N1
1961986|A/turkey/Italy/21VIR11804-1/2021|12/27/2021|H5N1
1944486|A/turkey/Italy/21VIR9210-1/2021|11/05/2021|H5N1
1944414|A/turkey/Italy/21VIR9143-2/2021|11/03/2021|H5N1
1961994|A/chicken/Italy/21VIR10850/2021|12/06/2021|H5N1
1962066|A/seagull/Italy/21VIR10481-2/2021|11/29/2021|H5N1
1962010|A/turkey/Italy/21VIR11887-3/2021|12/29/2021|H5N1
1944462|A/turkey/Italy/21VIR9649-2/2021|11/15/2021|H5N1
1944398|A/chicken/Italy/21VIR9691-2/2021|11/15/2021|H5N1
1944454|A/kestrel/Italy/21VIR10468/2021|11/30/2021|H5N1
1944422|A/chicken/Italy/21VIR9765-12/2021|11/17/2021|H5N1
1923193|A/turkey/Italy/21VIR8585-1/2021|10/18/2021|H5N1
1944273|A/chicken/Italy/21VIR10239/2021|11/25/2021|H5N1
1944281|A/turkey/Italy/21VIR10340/2021|11/29/2021|H5N1
1944366|A/seagull/Italy/21VIR9432-2/2021|11/11/2021|H5N1
1944494|A/turkey/Italy/21VIR8817-1/2021|10/25/2021|H5N1
1944390|A/turkey/Italy/21VIR9652-2/2021|11/15/2021|H5N1
1962002|A/chicken/Italy/21VIR10573-1/2021|12/03/2021|H5N1
1944358|A/chicken/Italy/21VIR9133-20/2021|11/04/2021|H5N1
1944374|A/turkey/Italy/21VIR9767-3/2021|11/17/2021|H5N1
2021104|A/duck/Czech_Republic/913/2022|01/12/2022|H5N1
2021875|A/chicken/Czech_Republic/913/2022|01/12/2022|H5N1
2021867|A/goose/Czech_Republic/913/2022|01/12/2022|H5N1
2021096|A/mute_swan/Czech_Republic/785/2022|01/10/2022|H5N1
1943063|A/chicken/Czech_Republic/23589-1T/2021|11/29/2021|H5N1
1943071|A/chicken/Czech_Republic/23589-3/2021|11/29/2021|H5N1
1980445|A/goose/Czech_Republic/23458-1K/2021|11/26/2021|H5N1
1999354|A/chicken/Czech_Republic/61-1/2022|12/31/2021|H5N1
1999362|A/chicken/Czech_Republic/61-2/2022|12/31/2021|H5N1
1998403|A/stork/Spain/234-2_22VIR2142-7/2022|01/17/2022|H5N1
1998203|A/swan/Spain/4087-1_22VIR2142-1/2021|12/22/2021|H5N1
1943079|A/chicken/Czech_Republic/23589-4/2021|11/29/2021|H5N1
1943055|A/duck/Czech_Republic/23589-1T/2021|11/29/2021|H5N1
1980671|A/mute_swan/Czech_Republic/785/2022|01/10/2022|H5N1
1980429|A/goose/Czech_Republic/23458-4T/2021|11/26/2021|H5N1
1980413|A/goose/Czech_Republic/23458-5T/2021|11/26/2021|H5N1
1980396|A/goose/Czech_Republic/23458-2T/2021|11/26/2021|H5N1
2026157|A/goose/Czech_Republic/25322-229/2021|12/18/2021|H5N1
2021904|A/goose/Czech_Republic/25322-205/2021|12/18/2021|H5N1
2021895|A/goose/Czech_Republic/25322-179/2021|12/18/2021|H5N1
1940274|A/chicken/Czech_Republic/22224-4T/2021|11/12/2021|H5N1
1940258|A/chicken/Czech_Republic/22224-3K/2021|11/12/2021|H5N1
1940250|A/chicken/Czech_Republic/22224-2T/2021|11/12/2021|H5N1
1940266|A/chicken/Czech_Republic/22224-3T/2021|11/12/2021|H5N1
1951286|A/goose/Czech_Republic/22608-1/2021|11/18/2021|H5N1
1980493|A/goose/Czech_Republic/22608-3T/2021|11/18/2021|H5N1
1980461|A/goose/Czech_Republic/22608-1T/2021|11/18/2021|H5N1
1980477|A/goose/Czech_Republic/22608-2T/2021|11/18/2021|H5N1
1951294|A/goose/Czech_Republic/22608-2/2021|11/18/2021|H5N1
1980574|A/goose/Czech_Republic/22750/2021|11/19/2021|H5N1
1937621|A/turkey/Poland/H1913-T1/2021|11/02/2021|H5N1
1937661|A/turkey/Poland/H1944-N/2021|11/08/2021|H5N1
1937605|A/turkey/Poland/H1910-T3/2021|11/01/2021|H5N1
1937629|A/turkey/Poland/H1924-T1/2021|11/03/2021|H5N1
1937613|A/turkey/Poland/H1911-N/2021|11/01/2021|H5N1
1922910|A/chicken/Tyumen/27-42V/2021|10/06/2021|H5N1
1922902|A/chicken/Tyumen/27-40V/2021|10/06/2021|H5N1
1922886|A/chicken/Tyumen/27-31V/2021|10/06/2021|H5N1
1922894|A/chicken/Tyumen/27-39V/2021|10/06/2021|H5N1
2015070|A/gray_heron/Denmark/24326-1.02/2021-10-28|10/28/2021|H5N1
1995117|A/swan/Slovenia/13_22VIR777-8/2022|01/03/2022|H5N1
2015018|A/swan/Romania/10656_22VIR2749-6/2022|02/22/2022|H5N1
1999370|A/chicken/Czech_Republic/63/2022|01/03/2022|H5N1
2014930|A/chicken/Poland/H157_22VIR2515-3/2022|02/18/2022|H5N1
1963353|A/Mallard/Netherlands/14/2021|12/27/2021|H5N1
1963361|A/Mallard/Netherlands/15/2021|12/27/2021|H5N1
1963345|A/Mallard/Netherlands/13/2021|12/27/2021|H5N1
1925533|A/chicken/Netherlands/21038165-006010/2021|11/07/2021|H5N1
1937653|A/domestic_duck/Poland/H1942-N/2021|11/07/2021|H5N1
1945366|A/Withe-tiled_eagle/Estonia/TA2124126-1_21VIR10433-11/2021|10/12/2021|H5N1
1938869|A/greylag_goose_/Sweden/SVA211118SZ0354/FB004497/I-2021|11/17/2021|H5N1
1998139|A/goose/Ireland/3869_22VIR2064-2/2022|02/14/2022|H5N1
1998131|A/fox/Ireland/3866_22VIR2064-1/2022|02/14/2022|H5N1
1967010|A/Caspian_gull/Netherlands/1/2022|01/14/2022|H5N1
1995038|A/Sanderling/Netherlands/1/2022|01/29/2022|H5N1
2001629|A/black-headed_gull/England/306270/2022|01/31/2022|H5N1
2001589|A/chicken/England/002070/2022|01/06/2022|H5N1
1966955|A/Great_black-backed_gull/1/2022|01/06/2022|H5N1
2008126|A/black-headed_gull/England/388256/2022|02/03/2022|H5N1
1997970|A/buzzard/Ireland/000656_22VIR1325-12/2022|01/07/2022|H5N1
2001621|A/domestic_duck/England/007588/2022|01/23/2022|H5N1
2001597|A/turkey/England/004737/2022|01/12/2022|H5N1
2001613|A/chicken/England/000187/2022|01/03/2022|H5N1
1998431|A/mute_swan/Ireland/037311_22VIR1325-13/2021|12/22/2021|H5N1
1965254|A/northern_goshawk/Netherlands/22000305-002/2022|01/04/2022|H5N1
1997978|A/mute_swan/Ireland/033169_22VIR1325-14/2021|11/15/2021|H5N1
1998051|A/duck/Ireland/036105_22VIR1325-22/2021|12/11/2021|H5N1
1998010|A/duck/Ireland/036646_22VIR1325-18/2021|12/17/2021|H5N1
1997962|A/crow/Ireland/035624_22VIR1325-11/2021|12/08/2021|H5N1
1998043|A/layer/Ireland/034424_22VIR1325-21/2021|11/29/2021|H5N1
1998002|A/turkey/Ireland/035425_22VIR1325-17/2021|12/06/2021|H5N1
1998018|A/turkey/Ireland/033674_22VIR1325-19/2021|11/19/2021|H5N1
1997954|A/herring_gull/Ireland/033533_22VIR1325-10/2021|11/17/2021|H5N1
1998099|A/greylag_goose/Ireland/032969_22VIR1325-6/2021|11/15/2021|H5N1
1998034|A/broiler/Ireland/033734_22VIR1325-20/2021|11/22/2021|H5N1
1963890|A/Whooper_swan/Scotland/056219/2021|11/09/2021|H5N1
1963850|A/chicken/England/057314/2021|11/14/2021|H5N1
1963898|A/Greylag_goose/England/054503/2021|10/30/2021|H5N1
1998026|A/mute_swan/Ireland/032363_22VIR1325-1/2021|11/08/2021|H5N1
1998107|A/greylag_goose/Ireland/033062_22VIR1325-7/2021|11/15/2021|H5N1
1959917|A/turkey/England/056764/2021|11/10/2021|H5N1
2011633|A/barnacle_goose/Denmark/24273-1.02/2021-10-26|10/26/2021|H5N1
2011657|A/common_buzzard/Denmark/24271-1.02/2021-10-25|10/25/2021|H5N1
1998091|A/magpie/Ireland/032958_22VIR1325-5/2021|11/12/2021|H5N1
1998083|A/whooper_swan/Ireland/032960_22VIR1325-4/2021|11/12/2021|H5N1
1998059|A/white-tailed_eagle/Ireland/032034_22VIR1325-23/2021|11/03/2021|H5N1
2011649|A/chicken/Denmark/24357-11/2021-11-02|11/02/2021|H5N1
2012868|A/turkey/Denmark/24325-25/2021-10-30|10/30/2021|H5N1
1994982|A/Mallard/Netherlands/1/2022|02/12/2022|H5N1
1994990|A/Mallard/Netherlands/2/2022|02/14/2022|H5N1
1967026|A/Barnacle_goose/Netherlands/6/2022|01/14/2022|H5N1
1995727|A/Greylag_Goose/Netherlands/1/2022|02/02/2022|H5N1
2018068|A/Fox/Netherlands/EMC2/2022|01/10/2022|H5N1
1967002|A/Grey_heron/Netherlands/1/2022|01/14/2022|H5N1
1966002|A/Gallus_gallus/Belgium/17100_0001/2021|12/27/2021|H5N1
1997263|A/duck/Bangladesh/19D1818/2021|12/22/2021|H5N1
1963866|A/domestic_duck/England/058612/2021|11/18/2021|H5N1
1927116|A/chicken/Netherlands/21038675-001005/2021|11/14/2021|H5N1
1927092|A/greylag_goose/Netherlands/21037497-001/2021|10/19/2021|H5N1
1929970|A/greylag_goose/Netherlands/21037809-001/2021|10/31/2021|H5N1
1980639|A/pheasant/Czech_Republic/25827-2/2021|12/25/2021|H5N1
1980623|A/pheasant/Czech_Republic/25827-1/2021|12/25/2021|H5N1
1980655|A/pheasant/Czech_Republic/25827-3/2021|12/25/2021|H5N1
2014986|A/swan/Romania/10324_22VIR2749-2/2022|02/01/2022|H5N1
2021885|A/mute_swan/Czech_Republic/2755/2022|01/29/2022|H5N1
1980590|A/mute_swan/Czech_Republic/25702-2/2021|12/20/2021|H5N1
1943047|A/chicken/Czech_Republic/23404-4T/2021|11/25/2021|H5N1
1943039|A/chicken/Czech_Republic/23404-2T/2021|11/25/2021|H5N1
1943031|A/chicken/Czech_Republic/23404-4K/2021|11/25/2021|H5N1
1951302|A/chicken/Czech_Republic/23404/2021|11/25/2021|H5N1
1980558|A/mute_swan/Czech_Republic/22684/2021|11/15/2021|H5N1
1943023|A/chicken/Czech_Republic/23404-2K/2021|11/25/2021|H5N1
1980509|A/mute_swan/Czech_Republic/22477-1/2021|11/12/2021|H5N1
1980526|A/mute_swan/Czech_Republic/22477-2/2021|11/12/2021|H5N1
1967018|A/Black-headed_gull/Netherlands/1/2022|01/14/2022|H5N1
1962114|A/buzzard/Italy/21VIR11899-5/2021|12/27/2021|H5N1
1962106|A/owl/Italy/21VIR11899-1/2021|12/27/2021|H5N1
1962026|A/turkey/Italy/21VIR10851/2021|12/06/2021|H5N1
1962034|A/laying_hen/Italy/21VIR11502/2021|12/21/2021|H5N1
1944233|A/chicken/Italy/21VIR10388/2021|11/30/2021|H5N1
1944257|A/turkey/Italy/21VIR9618-7/2021|11/18/2021|H5N1
1944241|A/duck/Italy/21VIR10447/2021|12/01/2021|H5N1
1944329|A/chicken/Italy/21VIR10343/2021|11/28/2021|H5N1
1944342|A/magpie/Italy/21VIR9487-2/2021|11/05/2021|H5N1
1944265|A/mallard/Italy/21VIR8919-2/2021|10/27/2021|H5N1
1998195|A/turkey/Spain/140-38_22VIR2142-19/2022|01/15/2022|H5N1
2014970|A/swan/Poland/MB083_22VIR2515-8/2022|02/17/2022|H5N1
1962090|A/seagull/Italy/21VIR11259-10/2021|12/15/2021|H5N1
1965995|A/Anser_albifrons/Belgium/15465_0010/2021|11/21/2021|H5N1
1930114|A/mute_swan/Netherlands/21038479-002/2021|11/09/2021|H5N1
1927125|A/mute_swan/Czech_Republic/21312/2021|11/01/2021|H5N1
1928149|A/swan/France/21P012384/2021|11/08/2021|H5N1
Aug,2021
A_Cygnus_columbianus_Hubei_126_2021_H5N1_
A_Cygnus_columbianus_Hubei_127_2021_H5N1_
A_Cygnus_columbianus_Hubei_117_2021_H5N1_
A_Cygnus_columbianus_Hubei_123_2021_H5N1_
A_Cygnus_columbianus_Hubei_128_2021_H5N1_
A_Cygnus_columbianus_Hubei_121_2021_H5N1_
1995085|A/swan/Slovenia/2060_22VIR777-4/2021|12/29/2021|H5N1
1995101|A/swan/Slovenia/2073_22VIR777-6/2021|12/30/2021|H5N1
1995069|A/swan/Slovenia/2041_22VIR777-2/2021|12/26/2021|H5N1
1995061|A/rooster/Slovenia/2039_22VIR777-1/2021|12/26/2021|H5N1
2015026|A/swan/Romania/10678_22VIR2749-7/2022|02/23/2022|H5N1
2014994|A/swan/Romania/10394_22VIR2749-3/2022|02/03/2022|H5N1
1944334|A/Wild_goose/Italy/21VIR10193/2021|11/23/2021|H5N1
1937645|A/chicken/Poland/H1940-N/2021|11/05/2021|H5N1
1937637|A/domestic_goose/Poland/H1931-T1/2021|11/03/2021|H5N1
1959907|A/turkey/England/055251/2021|11/06/2021|H5N1
1941516|A/mute_swan/Croatia/100/2021|11/12/2021|H5N1
2041979|A/turkey/Israel/537/2021|10/12/2021|H5N1
2021933|A/Ezo_red_fox/Hokkaido/1/2022|03/31/2022|H5N1
2021941|A/crow/Hokkaido/0103B065/2022|03/29/2022|H5N1
1999384|A/white-tailed_eagle/Hokkaido/22-RU-WTE-2/2022|01/02/2022|H5N1
1945578|A/Red_fox/Estonia/TA2126820_21VIR10433-13/2021|11/08/2021|H5N1
1896069|A/chicken/Nigeria/VRD21-109_21VIR2370-425/2021|03/01/2021|H5N1
1896088|A/chicken/Nigeria/VRD21-98_21VIR2288-6/2021|02/12/2021|H5N1
1896061|A/chicken/Nigeria/VRD21-102_21VIR2370-424/2021|03/01/2021|H5N1
1945382|A/gull/Estonia/TA2113284-4_21VIR7512-8/2021|06/02/2021|H5N1
1877840|A/White-Tailed_Eagle/Sweden/SVA210528SZ0223/KN002027/AB-2021|05/25/2021|H5N1
2015062|A/European_herring_gull/Denmark/19968-1.02/2021-05-14|05/14/2021|H5N1
1945478|A/Eurasian_eagle-owl/Finland/10617_21VIR7689-15/2021|08/01/2021|H5N1
1838674|A/chicken/Netherlands/20019879-001005/2020|12/14/2020|H5N1
1850254|A/barnacle_goose/Netherlands/21022611-001/2021|01/31/2021|H5N1
1859740|A/barnacle_goose/Netherlands/21024066-001/2021|02/22/2021|H5N1
1847709|A/barnacle_goose/Netherlands/21021591-001/2021|01/14/2021|H5N1
1807236|A/Eurasian_Wigeon/Netherlands/1/2020|10/16/2020|H5N1
1807252|A/Eurasian_Wigeon/Netherlands/5/2020|10/16/2020|H5N1
1859812|A/barnacle_goose/Netherlands/21025769-002/2021|03/22/2021|H5N1
1922852|A/barnacle_goose/Denmark/17572-1.01/2021-03-01|03/01/2021|H5N1
1921053|A/barnacle_goose/Germany-NI/AI03914/2021|04/08/2021|H5N1
1807244|A/Eurasian_Wigeon/Netherlands/4/2020|10/16/2020|H5N1
1812368|A/greylag_goose/Netherlands/20016582-004/2020|10/28/2020|H5N1
1987734|A/mute_swan/Czech_Republic/4099/2021|02/24/2021|H5N5
1866450|A/chicken/Senegal/21VIR1084-4/2021|12/23/2020|H5N1
1866458|A/chicken/Senegal/21VIR1084-5/2021|12/23/2020|H5N1
1866466|A/great-white_pelican/Senegal/21-67_21VIR1084-8/2021|01/23/2021|H5N1
0.2
2019.5
2020
2020.5
2021
2021.5
2022

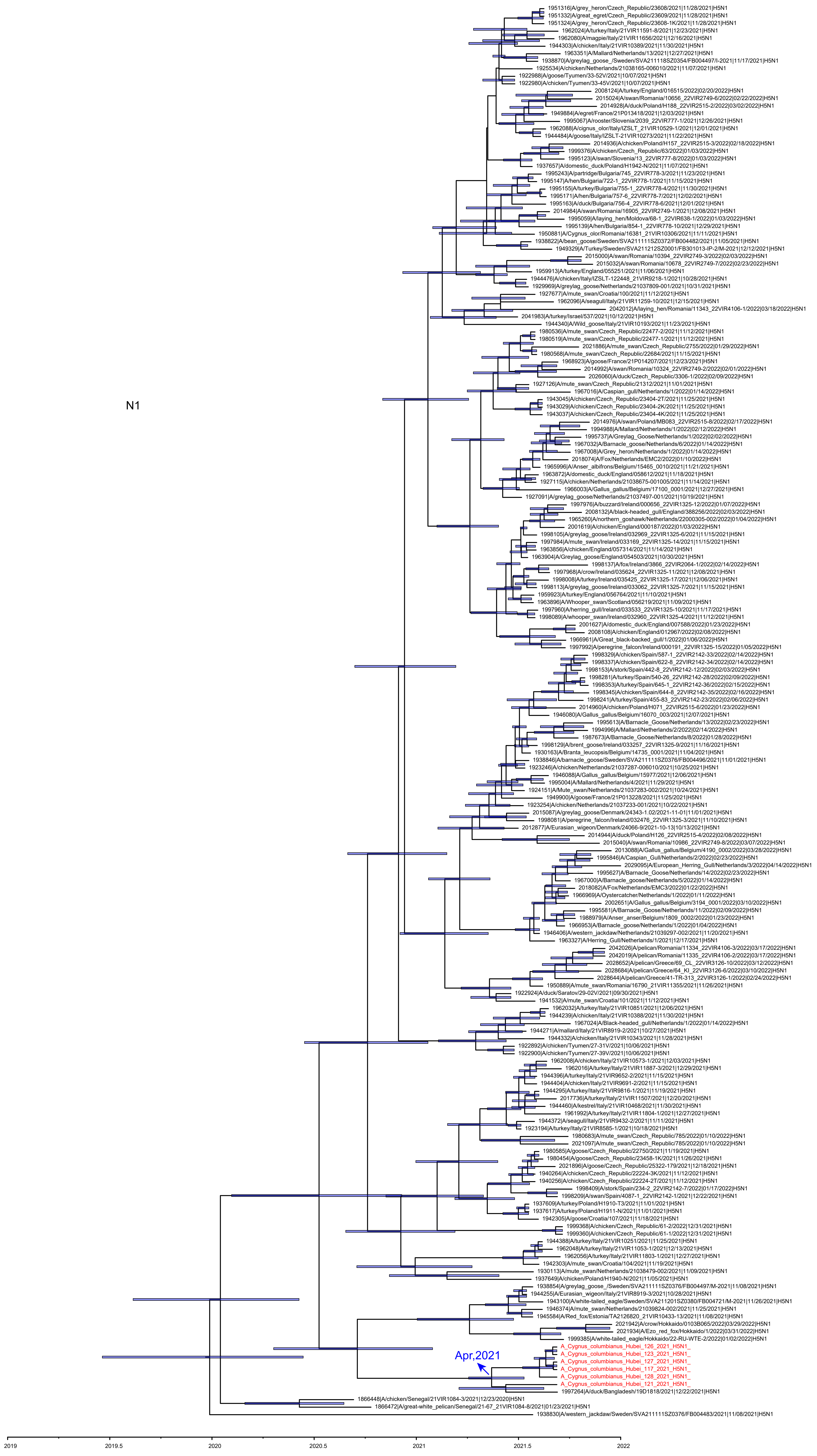
N1
Apr,2021
A_Cygnus_columbianus_Hubei_126_2021_H5N1_
A_Cygnus_columbianus_Hubei_123_2021_H5N1_
A_Cygnus_columbianus_Hubei_127_2021_H5N1_
A_Cygnus_columbianus_Hubei_117_2021_H5N1_
A_Cygnus_columbianus_Hubei_128_2021_H5N1_
A_Cygnus_columbianus_Hubei_121_2021_H5N1_
1997264|A/duck/Bangladesh/19D1818/2021|12/22/2021|H5N1
2021942|A/crow/Hokkaido/0103B065/2022|03/29/2022|H5N1
2021934|A/Ezo_red_fox/Hokkaido/1/2022|03/31/2022|H5N1
1999385|A/white-tailed_eagle/Hokkaido/22-RU-WTE-2/2022|01/02/2022|H5N1
1938830|A/western_jackdaw/Sweden/SVA211111SZ0376/FB004483/2021|11/08/2021|H5N1
2019
2019.5
2020
2020.5
2021
2021.5
2022

M

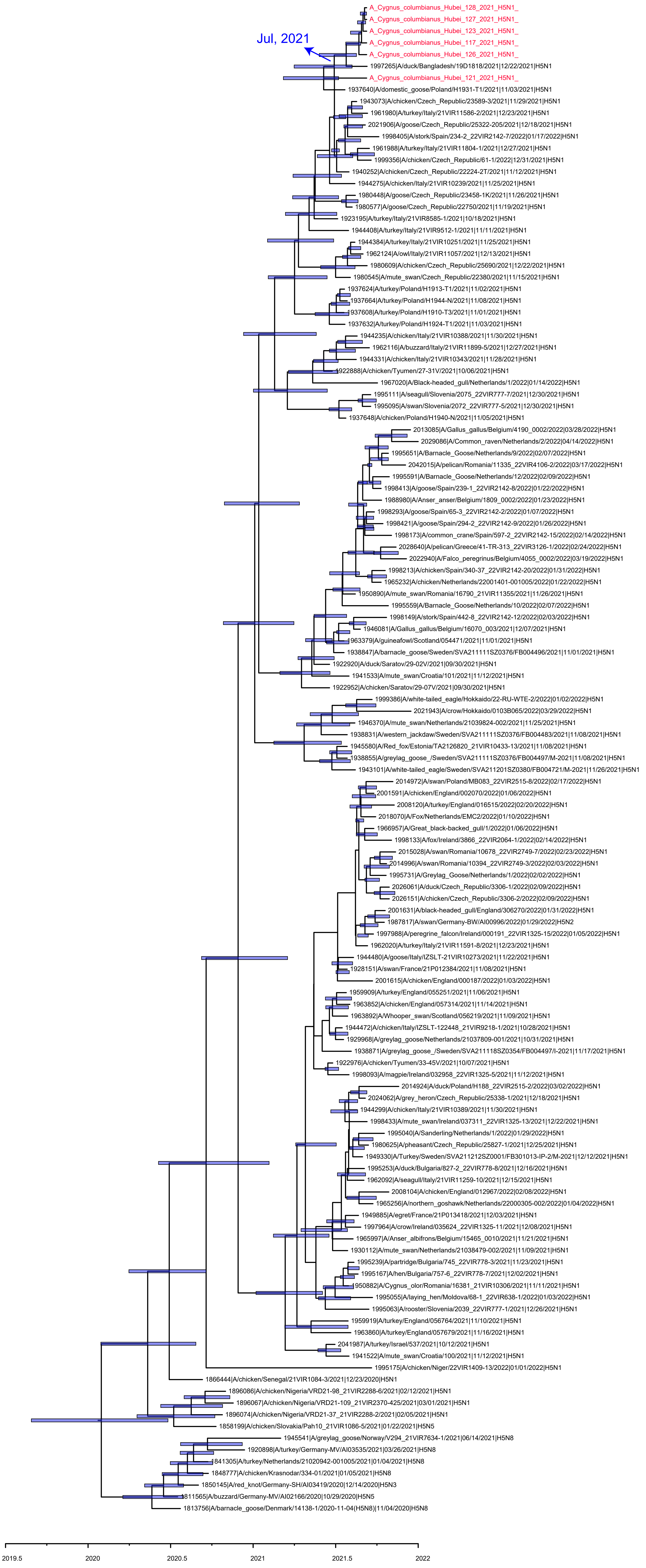

A_Cygnus_columbianus_Hubei_128_2021_H5N1_
A_Cygnus_columbianus_Hubei_127_2021_H5N1_
A_Cygnus_columbianus_Hubei_123_2021_H5N1_
Jul, 2021
A_Cygnus_columbianus_Hubei_117_2021_H5N1_
A_Cygnus_columbianus_Hubei_126_2021_H5N1_
1997265|A/duck/Bangladesh/19D1818/2021|12/22/2021|H5N1
A_Cygnus_columbianus_Hubei_121_2021_H5N1_
1937640|A/domestic_goose/Poland/H1931-T1/2021|11/03/2021|H5N1
1943073|A/chicken/Czech_Republic/23589-3/2021|11/29/2021|H5N1
1961980|A/turkey/Italy/21VIR11586-2/2021|12/23/2021|H5N1
2021906|A/goose/Czech_Republic/25322-205/2021|12/18/2021|H5N1
1998405|A/stork/Spain/234-2_22VIR2142-7/2022|01/17/2022|H5N1
1961988|A/turkey/Italy/21VIR11804-1/2021|12/27/2021|H5N1
1999356|A/chicken/Czech_Republic/61-1/2022|12/31/2021|H5N1
1940252|A/chicken/Czech_Republic/22224-2T/2021|11/12/2021|H5N1
1944275|A/chicken/Italy/21VIR10239/2021|11/25/2021|H5N1
1980448|A/goose/Czech_Republic/23458-1K/2021|11/26/2021|H5N1
1980577|A/goose/Czech_Republic/22750/2021|11/19/2021|H5N1
1923195|A/turkey/Italy/21VIR8585-1/2021|10/18/2021|H5N1
1944408|A/turkey/Italy/21VIR9512-1/2021|11/11/2021|H5N1
1944384|A/turkey/Italy/21VIR10251/2021|11/25/2021|H5N1
1962124|A/owl/Italy/21VIR11057/2021|12/13/2021|H5N1
1980609|A/chicken/Czech_Republic/25690/2021|12/22/2021|H5N1
1980545|A/mute_swan/Czech_Republic/22380/2021|11/15/2021|H5N1
1937624|A/turkey/Poland/H1913-T1/2021|11/02/2021|H5N1
1937664|A/turkey/Poland/H1944-N/2021|11/08/2021|H5N1
1937608|A/turkey/Poland/H1910-T3/2021|11/01/2021|H5N1
1937632|A/turkey/Poland/H1924-T1/2021|11/03/2021|H5N1
1944235|A/chicken/Italy/21VIR10388/2021|11/30/2021|H5N1
1962116|A/buzzard/Italy/21VIR11899-5/2021|12/27/2021|H5N1
1944331|A/chicken/Italy/21VIR10343/2021|11/28/2021|H5N1
1922888|A/chicken/Tyumen/27-31V/2021|10/06/2021|H5N1
1967020|A/Black-headed_gull/Netherlands/1/2022|01/14/2022|H5N1
1995111|A/seagull/Slovenia/2075_22VIR777-7/2021|12/30/2021|H5N1
1995095|A/swan/Slovenia/2072_22VIR777-5/2021|12/30/2021|H5N1
1937648|A/chicken/Poland/H1940-N/2021|11/05/2021|H5N1
2013085|A/Gallus_gallus/Belgium/4190_0002/2022|03/28/2022|H5N1
2029086|A/Common_raven/Netherlands/2/2022|04/14/2022|H5N1
1995651|A/Barnacle_Goose/Netherlands/9/2022|02/07/2022|H5N1
2042015|A/pelican/Romania/11335_22VIR4106-2/2022|03/17/2022|H5N1
1995591|A/Barnacle_Goose/Netherlands/12/2022|02/09/2022|H5N1
1998413|A/goose/Spain/239-1_22VIR2142-8/2022|01/22/2022|H5N1
1988980|A/Anser_anser/Belgium/1809_0002/2022|01/23/2022|H5N1
1998293|A/goose/Spain/65-3_22VIR2142-2/2022|01/07/2022|H5N1
1998421|A/goose/Spain/294-2_22VIR2142-9/2022|01/26/2022|H5N1
1998173|A/common_crane/Spain/597-2_22VIR2142-15/2022|02/14/2022|H5N1
2028640|A/pelican/Greece/41-TR-313_22VIR3126-1/2022|02/24/2022|H5N1
2022940|A/Falco_peregrinus/Belgium/4055_0002/2022|03/19/2022|H5N1
1998213|A/chicken/Spain/340-37_22VIR2142-20/2022|01/31/2022|H5N1
1965232|A/chicken/Netherlands/22001401-001005/2022|01/22/2022|H5N1
1950890|A/mute_swan/Romania/16790_21VIR11355/2021|11/26/2021|H5N1
1995559|A/Barnacle_Goose/Netherlands/10/2022|02/07/2022|H5N1
1998149|A/stork/Spain/442-8_22VIR2142-12/2022|02/03/2022|H5N1
1946081|A/Gallus_gallus/Belgium/16070_003/2021|12/07/2021|H5N1
1963379|A/guineafowl/Scotland/054471/2021|11/01/2021|H5N1
1938847|A/barnacle_goose/Sweden/SVA211111SZ0376/FB004496/2021|11/01/2021|H5N1
1922920|A/duck/Saratov/29-02V/2021|09/30/2021|H5N1
1941533|A/mute_swan/Croatia/101/2021|11/12/2021|H5N1
1922952|A/chicken/Saratov/29-07V/2021|09/30/2021|H5N1
1999386|A/white-tailed_eagle/Hokkaido/22-RU-WTE-2/2022|01/02/2022|H5N1
2021943|A/crow/Hokkaido/0103B065/2022|03/29/2022|H5N1
1946370|A/mute_swan/Netherlands/21039824-002/2021|11/25/2021|H5N1
1938831|A/western_jackdaw/Sweden/SVA211111SZ0376/FB004483/2021|11/08/2021|H5N1
1945580|A/Red_fox/Estonia/TA2126820_21VIR10433-13/2021|11/08/2021|H5N1
1938855|A/greylag_goose_/Sweden/SVA211111SZ0376/FB004497/M-2021|11/08/2021|H5N1
1943101|A/white-tailed_eagle/Sweden/SVA211201SZ0380/FB004721/M-2021|11/26/2021|H5N1
2014972|A/swan/Poland/MB083_22VIR2515-8/2022|02/17/2022|H5N1
2001591|A/chicken/England/002070/2022|01/06/2022|H5N1
2008120|A/turkey/England/016515/2022|02/20/2022|H5N1
2018070|A/Fox/Netherlands/EMC2/2022|01/10/2022|H5N1
1966957|A/Great_black-backed_gull/1/2022|01/06/2022|H5N1
1998133|A/fox/Ireland/3866_22VIR2064-1/2022|02/14/2022|H5N1
2015028|A/swan/Romania/10678_22VIR2749-7/2022|02/23/2022|H5N1
2014996|A/swan/Romania/10394_22VIR2749-3/2022|02/03/2022|H5N1
1995731|A/Greylag_Goose/Netherlands/1/2022|02/02/2022|H5N1
2026061|A/duck/Czech_Republic/3306-1/2022|02/09/2022|H5N1
2026151|A/chicken/Czech_Republic/3306-2/2022|02/09/2022|H5N1
2001631|A/black-headed_gull/England/306270/2022|01/31/2022|H5N1
1987817|A/swan/Germany-BW/AI00996/2022|01/29/2022|H5N2
1997988|A/peregrine_falcon/Ireland/000191_22VIR1325-15/2022|01/05/2022|H5N1
1962020|A/turkey/Italy/21VIR11591-8/2021|12/23/2021|H5N1
1944480|A/goose/Italy/IZSLT-21VIR10273/2021|11/22/2021|H5N1
1928151|A/swan/France/21P012384/2021|11/08/2021|H5N1
2001615|A/chicken/England/000187/2022|01/03/2022|H5N1
1959909|A/turkey/England/055251/2021|11/06/2021|H5N1
1963852|A/chicken/England/057314/2021|11/14/2021|H5N1
1963892|A/Whooper_swan/Scotland/056219/2021|11/09/2021|H5N1
1944472|A/chicken/Italy/IZSLT-122448_21VIR9218-1/2021|10/28/2021|H5N1
1929968|A/greylag_goose/Netherlands/21037809-001/2021|10/31/2021|H5N1
1938871|A/greylag_goose_/Sweden/SVA211118SZ0354/FB004497/I-2021|11/17/2021|H5N1
1922976|A/chicken/Tyumen/33-45V/2021|10/07/2021|H5N1
1998093|A/magpie/Ireland/032958_22VIR1325-5/2021|11/12/2021|H5N1
2014924|A/duck/Poland/H188_22VIR2515-2/2022|03/02/2022|H5N1
2024062|A/grey_heron/Czech_Republic/25338-1/2021|12/18/2021|H5N1
1944299|A/chicken/Italy/21VIR10389/2021|11/30/2021|H5N1
1998433|A/mute_swan/Ireland/037311_22VIR1325-13/2021|12/22/2021|H5N1
1995040|A/Sanderling/Netherlands/1/2022|01/29/2022|H5N1
1980625|A/pheasant/Czech_Republic/25827-1/2021|12/25/2021|H5N1
1949330|A/Turkey/Sweden/SVA211212SZ0001/FB301013-IP-2/M-2021|12/12/2021|H5N1
1995253|A/duck/Bulgaria/827-2_22VIR778-8/2021|12/16/2021|H5N1
1962092|A/seagull/Italy/21VIR11259-10/2021|12/15/2021|H5N1
2008104|A/chicken/England/012967/2022|02/08/2022|H5N1
1965256|A/northern_goshawk/Netherlands/22000305-002/2022|01/04/2022|H5N1
1949885|A/egret/France/21P013418/2021|12/03/2021|H5N1
1997964|A/crow/Ireland/035624_22VIR1325-11/2021|12/08/2021|H5N1
1965997|A/Anser_albifrons/Belgium/15465_0010/2021|11/21/2021|H5N1
1930112|A/mute_swan/Netherlands/21038479-002/2021|11/09/2021|H5N1
1995239|A/partridge/Bulgaria/745_22VIR778-3/2021|11/23/2021|H5N1
1995167|A/hen/Bulgaria/757-6_22VIR778-7/2021|12/02/2021|H5N1
1950882|A/Cygnus_olor/Romania/16381_21VIR10306/2021|11/11/2021|H5N1
1995055|A/laying_hen/Moldova/68-1_22VIR638-1/2022|01/03/2022|H5N1
1995063|A/rooster/Slovenia/2039_22VIR777-1/2021|12/26/2021|H5N1
1959919|A/turkey/England/056764/2021|11/10/2021|H5N1
1963860|A/turkey/England/057679/2021|11/16/2021|H5N1
2041987|A/turkey/Israel/537/2021|10/12/2021|H5N1
1941522|A/mute_swan/Croatia/100/2021|11/12/2021|H5N1
1995175|A/chicken/Niger/22VIR1409-13/2022|01/01/2022|H5N1
1866444|A/chicken/Senegal/21VIR1084-3/2021|12/23/2020|H5N1
1896086|A/chicken/Nigeria/VRD21-98_21VIR2288-6/2021|02/12/2021|H5N1
1896067|A/chicken/Nigeria/VRD21-109_21VIR2370-425/2021|03/01/2021|H5N1
1896074|A/chicken/Nigeria/VRD21-37_21VIR2288-2/2021|02/05/2021|H5N1
1858199|A/chicken/Slovakia/Pah10_21VIR1086-5/2021|01/22/2021|H5N5
1945541|A/greylag_goose/Norway/V294_21VIR7634-1/2021|06/14/2021|H5N8
1920898|A/turkey/Germany-MV/AI03535/2021|03/26/2021|H5N8
1841305|A/turkey/Netherlands/21020942-001005/2021|01/04/2021|H5N8
1848777|A/chicken/Krasnodar/334-01/2021|01/05/2021|H5N8
1850145|A/red_knot/Germany-SH/AI03419/2020|12/14/2020|H5N3
1811565|A/buzzard/Germany-MV/AI02166/2020|10/29/2020|H5N5
1813756|A/barnacle_goose/Denmark/14138-1/2020-11-04(H5N8)|11/04/2020|H5N8
2019.5
2020
2020.5
2021
2021.5
2022

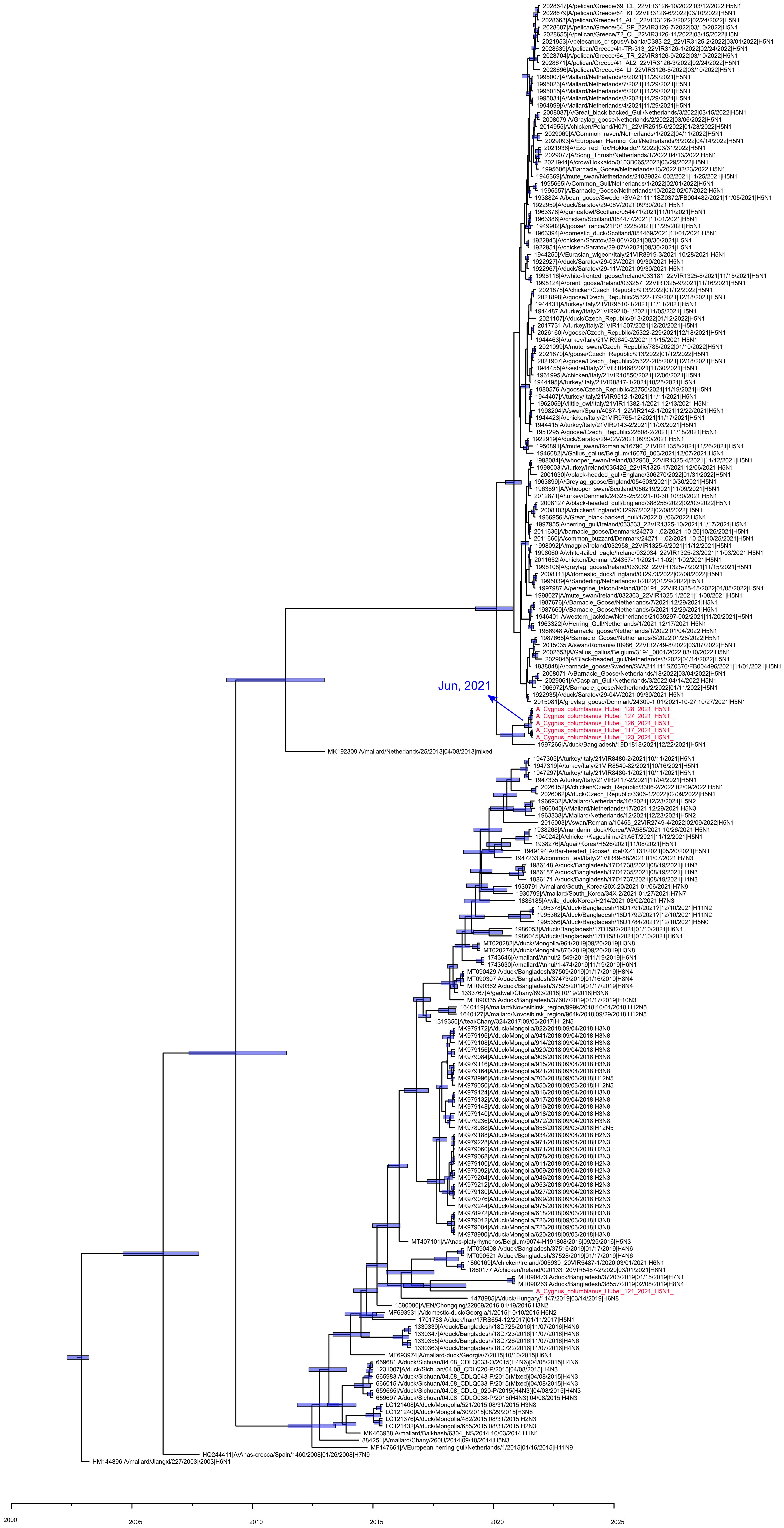

2028647|A/pelican/Greece/69_CL_22VIR3126-10/2022|03/12/2022|H5N1
2028679|A/pelican/Greece/64_KI_22VIR3126-6/2022|03/10/2022|H5N1
2028663|A/pelican/Greece/41_AL1_22VIR3126-2/2022|02/24/2022|H5N1
2028687|A/pelican/Greece/64_SP_22VIR3126-7/2022|03/10/2022|H5N1
2028655|A/pelican/Greece/72_CL_22VIR3126-11/2022|03/15/2022|H5N1
2021953|A/pelecanus_crispus/Albania/D383-22_22VIR3125-2/2022|03/01/2022|H5N1
2028639|A/pelican/Greece/41-TR-313_22VIR3126-1/2022|02/24/2022|H5N1
2028704|A/pelican/Greece/64_TR_22VIR3126-9/2022|03/10/2022|H5N1
2028671|A/pelican/Greece/41_AL2_22VIR3126-3/2022|02/24/2022|H5N1
2028696|A/pelican/Greece/64_LI_22VIR3126-8/2022|03/10/2022|H5N1
1995007|A/Mallard/Netherlands/5/2021|11/29/2021|H5N1
1995023|A/Mallard/Netherlands/7/2021|11/29/2021|H5N1
1995015|A/Mallard/Netherlands/6/2021|11/29/2021|H5N1
1995031|A/Mallard/Netherlands/8/2021|11/29/2021|H5N1
1994999|A/Mallard/Netherlands/4/2021|11/29/2021|H5N1
2008087|A/Great_black-backed_Gull/Netherlands/3/2022|03/15/2022|H5N1
2008079|A/Graylag_goose/Netherlands/2/2022|03/06/2022|H5N1
2014955|A/chicken/Poland/H071_22VIR2515-6/2022|01/23/2022|H5N1
2029069|A/Common_raven/Netherlands/1/2022|04/11/2022|H5N1
2029093|A/European_Herring_Gull/Netherlands/3/2022|04/14/2022|H5N1
2021936|A/Ezo_red_fox/Hokkaido/1/2022|03/31/2022|H5N1
2029077|A/Song_Thrush/Netherlands/1/2022|04/13/2022|H5N1
2021944|A/crow/Hokkaido/0103B065/2022|03/29/2022|H5N1
1995606|A/Barnacle_Goose/Netherlands/13/2022|02/23/2022|H5N1
1946369|A/mute_swan/Netherlands/21039824-002/2021|11/25/2021|H5N1
1995665|A/Common_Gull/Netherlands/1/2022|02/01/2022|H5N1
1995557|A/Barnacle_Goose/Netherlands/10/2022|02/07/2022|H5N1
1938824|A/bean_goose/Sweden/SVA211111SZ0372/FB004482/2021|11/05/2021|H5N1
1922959|A/duck/Saratov/29-08V/2021|09/30/2021|H5N1
1963378|A/guineafowl/Scotland/054471/2021|11/01/2021|H5N1
1963386|A/chicken/Scotland/054477/2021|11/01/2021|H5N1
1949902|A/goose/France/21P013228/2021|11/25/2021|H5N1
1963394|A/domestic_duck/Scotland/054469/2021|11/01/2021|H5N1
1922943|A/chicken/Saratov/29-06V/2021|09/30/2021|H5N1
1922951|A/chicken/Saratov/29-07V/2021|09/30/2021|H5N1
1944250|A/Eurasian_wigeon/Italy/21VIR8919-3/2021|10/28/2021|H5N1
1922927|A/duck/Saratov/29-03V/2021|09/30/2021|H5N1
1922967|A/duck/Saratov/29-11V/2021|09/30/2021|H5N1
1998116|A/white-fronted_goose/Ireland/033181_22VIR1325-8/2021|11/15/2021|H5N1
1998124|A/brent_goose/Ireland/033257_22VIR1325-9/2021|11/16/2021|H5N1
2021878|A/chicken/Czech_Republic/913/2022|01/12/2022|H5N1
2021898|A/goose/Czech_Republic/25322-179/2021|12/18/2021|H5N1
1944431|A/turkey/Italy/21VIR9510-1/2021|11/11/2021|H5N1
1944487|A/turkey/Italy/21VIR9210-1/2021|11/05/2021|H5N1
2021107|A/duck/Czech_Republic/913/2022|01/12/2022|H5N1
2017731|A/turkey/Italy/21VIR11507/2021|12/20/2021|H5N1
2026160|A/goose/Czech_Republic/25322-229/2021|12/18/2021|H5N1
1944463|A/turkey/Italy/21VIR9649-2/2021|11/15/2021|H5N1
2021099|A/mute_swan/Czech_Republic/785/2022|01/10/2022|H5N1
2021870|A/goose/Czech_Republic/913/2022|01/12/2022|H5N1
2021907|A/goose/Czech_Republic/25322-205/2021|12/18/2021|H5N1
1944455|A/kestrel/Italy/21VIR10468/2021|11/30/2021|H5N1
1961995|A/chicken/Italy/21VIR10850/2021|12/06/2021|H5N1
1944495|A/turkey/Italy/21VIR8817-1/2021|10/25/2021|H5N1
1980576|A/goose/Czech_Republic/22750/2021|11/19/2021|H5N1
1944407|A/turkey/Italy/21VIR9512-1/2021|11/11/2021|H5N1
1962059|A/little_owl/Italy/21VIR11382-1/2021|12/13/2021|H5N1
1998204|A/swan/Spain/4087-1_22VIR2142-1/2021|12/22/2021|H5N1
1944423|A/chicken/Italy/21VIR9765-12/2021|11/17/2021|H5N1
1944415|A/turkey/Italy/21VIR9143-2/2021|11/03/2021|H5N1
1951295|A/goose/Czech_Republic/22608-2/2021|11/18/2021|H5N1
1922919|A/duck/Saratov/29-02V/2021|09/30/2021|H5N1
1950891|A/mute_swan/Romania/16790_21VIR11355/2021|11/26/2021|H5N1
1946082|A/Gallus_gallus/Belgium/16070_003/2021|12/07/2021|H5N1
1998084|A/whooper_swan/Ireland/032960_22VIR1325-4/2021|11/12/2021|H5N1
1998003|A/turkey/Ireland/035425_22VIR1325-17/2021|12/06/2021|H5N1
2001630|A/black-headed_gull/England/306270/2022|01/31/2022|H5N1
1963899|A/Greylag_goose/England/054503/2021|10/30/2021|H5N1
1963891|A/Whooper_swan/Scotland/056219/2021|11/09/2021|H5N1
2012871|A/turkey/Denmark/24325-25/2021-10-30|10/30/2021|H5N1
2008127|A/black-headed_gull/England/388256/2022|02/03/2022|H5N1
2008103|A/chicken/England/012967/2022|02/08/2022|H5N1
1966956|A/Great_black-backed_gull/1/2022|01/06/2022|H5N1
1997955|A/herring_gull/Ireland/033533_22VIR1325-10/2021|11/17/2021|H5N1
2011636|A/barnacle_goose/Denmark/24273-1.02/2021-10-26|10/26/2021|H5N1
2011660|A/common_buzzard/Denmark/24271-1.02/2021-10-25|10/25/2021|H5N1
1998092|A/magpie/Ireland/032958_22VIR1325-5/2021|11/12/2021|H5N1
1998060|A/white-tailed_eagle/Ireland/032034_22VIR1325-23/2021|11/03/2021|H5N1
2011652|A/chicken/Denmark/24357-11/2021-11-02|11/02/2021|H5N1
1998108|A/greylag_goose/Ireland/033062_22VIR1325-7/2021|11/15/2021|H5N1
2008111|A/domestic_duck/England/012973/2022|02/08/2022|H5N1
1995039|A/Sanderling/Netherlands/1/2022|01/29/2022|H5N1
1997987|A/peregrine_falcon/Ireland/000191_22VIR1325-15/2022|01/05/2022|H5N1
1998027|A/mute_swan/Ireland/032363_22VIR1325-1/2021|11/08/2021|H5N1
1987676|A/Barnacle_Goose/Netherlands/7/2021|12/29/2021|H5N1
1987660|A/Barnacle_Goose/Netherlands/6/2021|12/29/2021|H5N1
1946401|A/western_jackdaw/Netherlands/21039297-002/2021|11/20/2021|H5N1
1963322|A/Herring_Gull/Netherlands/1/2021|12/17/2021|H5N1
1966948|A/Barnacle_goose/Netherlands/1/2022|01/04/2022|H5N1
1987668|A/Barnacle_Goose/Netherlands/8/2022|01/28/2022|H5N1
2015035|A/swan/Romania/10986_22VIR2749-8/2022|03/07/2022|H5N1
2002653|A/Gallus_gallus/Belgium/3194_0001/2022|03/10/2022|H5N1
2029045|A/Black-headed_gull/Netherlands/3/2022|04/14/2022|H5N1
1938848|A/barnacle_goose/Sweden/SVA211111SZ0376/FB004496/2021|11/01/2021|H5N1
2008071|A/Barnacle_Goose/Netherlands/18/2022|03/04/2022|H5N1
2029061|A/Caspian_Gull/Netherlands/3/2022|04/14/2022|H5N1
1966972|A/Barnacle_goose/Netherlands/2/2022|01/11/2022|H5N1
1922935|A/duck/Saratov/29-04V/2021|09/30/2021|H5N1
2015081|A/greylag_goose/Denmark/24309-1.01/2021-10-27|10/27/2021|H5N1
Jun, 2021
A_Cygnus_columbianus_Hubei_128_2021_H5N1_
A_Cygnus_columbianus_Hubei_127_2021_H5N1_
A_Cygnus_columbianus_Hubei_126_2021_H5N1_
A_Cygnus_columbianus_Hubei_117_2021_H5N1_
A_Cygnus_columbianus_Hubei_123_2021_H5N1_
1997266|A/duck/Bangladesh/19D1818/2021|12/22/2021|H5N1
MK192309|A/mallard/Netherlands/25/2013|04/08/2013|mixed
1947305|A/turkey/Italy/21VIR8480-2/2021|10/11/2021|H5N1
1947319|A/turkey/Italy/21VIR8540-82/2021|10/16/2021|H5N1
1947297|A/turkey/Italy/21VIR8480-1/2021|10/11/2021|H5N1
1947335|A/turkey/Italy/21VIR9117-2/2021|11/04/2021|H5N1
2026152|A/chicken/Czech_Republic/3306-2/2022|02/09/2022|H5N1
2026062|A/duck/Czech_Republic/3306-1/2022|02/09/2022|H5N1
1966932|A/Mallard/Netherlands/16/2021|12/23/2021|H5N2
1966940|A/Mallard/Netherlands/17/2021|12/29/2021|H5N3
1963338|A/Mallard/Netherlands/12/2021|12/23/2021|H5N2
2015003|A/swan/Romania/10455_22VIR2749-4/2022|02/09/2022|H5N1
1938268|A/mandarin_duck/Korea/WA585/2021|10/26/2021|H5N1
1940242|A/chicken/Kagoshima/21A6T/2021|11/12/2021|H5N1
1938276|A/quail/Korea/H526/2021|11/08/2021|H5N1
1949194|A/Bar-headed_Goose/Tibet/XZ1131/2021|05/20/2021|H5N1
1947233|A/common_teal/Italy/21VIR49-88/2021|01/07/2021|H7N3
1986148|A/duck/Bangladesh/17D1738/2021|08/19/2021|H1N3
1986187|A/duck/Bangladesh/17D1735/2021|08/19/2021|H1N3
1986171|A/duck/Bangladesh/17D1737/2021|08/19/2021|H1N3
1930791|A/mallard/South_Korea/20X-20/2021|01/06/2021|H7N9
1930799|A/mallard/South_Korea/34X-2/2021|01/27/2021|H7N7
1886185|A/wild_duck/Korea/H214/2021|03/02/2021|H7N3
1995378|A/duck/Bangladesh/18D1791/2021?|12/10/2021|H11N2
1995362|A/duck/Bangladesh/18D1792/2021?|12/10/2021|H11N2
1995356|A/duck/Bangladesh/18D1784/2021?|12/10/2021|H5N0
1986053|A/duck/Bangladesh/17D1582/2021|01/10/2021|H6N1
1986045|A/duck/Bangladesh/17D1581/2021|01/10/2021|H6N1
MT020282|A/duck/Mongolia/961/2019|09/20/2019|H3N8
MT020274|A/duck/Mongolia/876/2019|09/20/2019|H3N8
1743646|A/mallard/Anhui/2-549/2019|11/19/2019|H6N1
1743630|A/mallard/Anhui/1-474/2019|11/19/2019|H6N1
MT090429|A/duck/Bangladesh/37509/2019|01/17/2019|H8N4
MT090307|A/duck/Bangladesh/37473/2019|01/16/2019|H8N4
MT090362|A/duck/Bangladesh/37525/2019|01/17/2019|H8N4
1333767|A/gadwall/Chany/893/2018|10/19/2018|H3N8
MT090335|A/duck/Bangladesh/37607/2019|01/17/2019|H10N3
1640119|A/mallard/Novosibirsk_region/999k/2018|10/01/2018|H12N5
1640127|A/mallard/Novosibirsk_region/964k/2018|09/29/2018|H12N5
1319356|A/teal/Chany/324/2017|09/03/2017|H12N5
MK979172|A/duck/Mongolia/922/2018|09/04/2018|H3N8
MK979196|A/duck/Mongolia/941/2018|09/04/2018|H3N8
MK979108|A/duck/Mongolia/914/2018|09/04/2018|H3N8
MK979156|A/duck/Mongolia/920/2018|09/04/2018|H3N8
MK979084|A/duck/Mongolia/906/2018|09/04/2018|H3N8
MK979116|A/duck/Mongolia/915/2018|09/04/2018|H3N8
MK979164|A/duck/Mongolia/921/2018|09/04/2018|H3N8
MK978996|A/duck/Mongolia/703/2018|09/03/2018|H12N5
MK979050|A/duck/Mongolia/850/2018|09/03/2018|H12N5
MK979124|A/duck/Mongolia/916/2018|09/04/2018|H3N8
MK979132|A/duck/Mongolia/917/2018|09/04/2018|H3N8
MK979148|A/duck/Mongolia/919/2018|09/04/2018|H3N8
MK979140|A/duck/Mongolia/918/2018|09/04/2018|H3N8
MK979236|A/duck/Mongolia/972/2018|09/04/2018|H3N8
MK978988|A/duck/Mongolia/656/2018|09/03/2018|H12N5
MK979188|A/duck/Mongolia/934/2018|09/04/2018|H2N3
MK979228|A/duck/Mongolia/971/2018|09/04/2018|H2N3
MK979060|A/duck/Mongolia/871/2018|09/04/2018|H2N3
MK979068|A/duck/Mongolia/878/2018|09/04/2018|H2N3
MK979100|A/duck/Mongolia/911/2018|09/04/2018|H2N3
MK979092|A/duck/Mongolia/909/2018|09/04/2018|H2N3
MK979204|A/duck/Mongolia/946/2018|09/04/2018|H2N3
MK979212|A/duck/Mongolia/953/2018|09/04/2018|H2N3
MK979180|A/duck/Mongolia/927/2018|09/04/2018|H2N3
MK979076|A/duck/Mongolia/899/2018|09/04/2018|H2N3
MK979244|A/duck/Mongolia/975/2018|09/04/2018|H2N3
MK978972|A/duck/Mongolia/618/2018|09/03/2018|H3N8
MK979012|A/duck/Mongolia/726/2018|09/03/2018|H3N8
MK979004|A/duck/Mongolia/723/2018|09/03/2018|H3N8
MK978980|A/duck/Mongolia/620/2018|09/03/2018|H3N8
MT407101|A/Anas-platyrhynchos/Belgium/9074-H191808/2016|09/25/2016|H5N3
MT090408|A/duck/Bangladesh/37516/2019|01/17/2019|H4N6
MT090521|A/duck/Bangladesh/37528/2019|01/17/2019|H4N6
1860169|A/chicken/Ireland/005930_20VIR5487-1/2020|03/01/2021|H6N1
1860177|A/chicken/Ireland/020133_20VIR5487-2/2020|03/01/2021|H6N1
MT090473|A/duck/Bangladesh/37203/2019|01/15/2019|H7N1
MT090263|A/duck/Bangladesh/38557/2019|02/08/2019|H8N4
A_Cygnus_columbianus_Hubei_121_2021_H5N1_
1478985|A/duck/Hungary/1147/2019|03/14/2019|H6N8
1590090|A/EN/Chongqing/22909/2016|01/19/2016|H3N2
MF693931|A/domestic-duck/Georgia/1/2015|10/10/2015|H6N2
1701783|A/duck/Iran/17RS654-12/2017|01/11/2017|H5N1
1330339|A/duck/Bangladesh/18D725/2016|11/07/2016|H4N6
1330347|A/duck/Bangladesh/18D723/2016|11/07/2016|H4N6
1330355|A/duck/Bangladesh/18D724/2016|11/07/2016|H4N6
1330363|A/duck/Bangladesh/18D722/2016|11/07/2016|H4N6
MF693974|A/mallard-duck/Georgia/7/2015|10/10/2015|H6N1
659681|A/duck/Sichuan/04.08_CDLQ033-O/2015(H4N6)|04/08/2015|H4N6
1231007|A/duck/Sichuan/04.08_CDLQ20-P/2015|04/08/2015|H4N3
665983|A/duck/Sichuan/04.08_CDLQ043-P/2015(Mixed)|04/08/2015|H4N3
666015|A/duck/Sichuan/04.08_CDLQ033-P/2015(Mixed)|04/08/2015|H4N3
659665|A/duck/Sichuan/04.08_CDLQ_020-P/2015(H4N3)|04/08/2015|H4N3
659697|A/duck/Sichuan/04.08_CDLQ038-P/2015(H4N3)|04/08/2015|H4N3
LC121408|A/duck/Mongolia/521/2015|08/31/2015|H3N8
LC121240|A/duck/Mongolia/30/2015|08/29/2015|H3N8
LC121376|A/duck/Mongolia/482/2015|08/31/2015|H2N3
LC121432|A/duck/Mongolia/655/2015|08/31/2015|H2N3
MK463938|A/mallard/Balkhash/6304_NS/2014|10/03/2014|H1N1
884251|A/mallard/Chany/260U/2014|09/10/2014|H5N3
MF147661|A/European-herring-gull/Netherlands/1/2015|01/16/2015|H11N9
HQ244411|A/Anas-crecca/Spain/1460/2008|01/26/2008|H7N9
HM144896|A/mallard/Jiangxi/227/2003|/2003|H6N1
2000
2005
2010
2015
2020
2025

Table S1. H5N1 viruses were detected in migratory birds from Nov 4, 2021 to Nov 16, 2021, in Central China.

Taxa of isolates	Location	Time	Dead or rescued
A/Cygnus columbianus/Hubei/121/2021(H5N1)	Longgan Lake National Nature Reserve Administration	2021/11/04	Rescued
A/Cygnus columbianus/Hubei/117/2021(H5N1)	Longgan Lake National Nature Reserve Administration	2021/11/16	Dead
A/Cygnus columbianus/Hubei/123/2021(H5N1)	Longgan Lake National Nature Reserve Administration	2021/11/16	Dead
A/Cygnus columbianus/Hubei/127/2021(H5N1)	Longgan Lake National Nature Reserve Administration	2021/11/16	Dead
A/Cygnus columbianus/Hubei/128/2021(H5N1)	Longgan Lake National Nature Reserve Administration	2021/11/16	Dead
A/Cygnus columbianus/Hubei/126/2021(H5N1)	Longgan Lake National Nature Reserve Administration	2021/11/22	Dead

Table S2. Molecular dating analysis for the H5N1 viruses, related to Figure S2. Time of most recent common ancestors (tMRCA) between H5N1 viruses and their closest strains are shown.

Gene	Genotype	tMRCA	95% HPD interval	tMRCA to the closest outlier	95% HPD interval
PB2	G1	Jul.2021	[May.2021, Sep.2021]	Sep.2017	[Mar.2017, Apr.2018]
	G2	/	/	Jan.2021	[Sep.2020, Jul.2021]
PB1	G1	Jun.2021	[Apr.2021, Aug.2021]	Feb.2021	[Oct.2020, Jun.2021]
	G2	/	/	May.2021	[Jul.2019, Mar.2021]
PA	G1<br>G2	Jun.2021	[Mar.2021, Aug.2021]	Dec,2020	[Jul.2020, Apr.2021]
HA	G1<br>G2	Apr.2021	[Feb.2021, May.2021]	Dec,2020	[Jul.2020, Apr.2021]
NP	G1<br>G2	Aug.2021	[Jun.2021, Sep.2021]	Aug.2021	[May.2021, Sep.2021]
N1	G1<br>G2	Apr.2021	[Feb.2021, Jun.2021]	Sep.2020	[Apr,2020, Jan.2021]
M	G1<br>G2	Jul.2021	[Apr.2021, Aug.2021]	June.2021	[Mar.2021, Jul.2021]
NS	G1	Jun.2021	[Apr.2021, Aug.2021]	Oct.2020	[Apr,2020, Apr.2021]
	G2	/	/	May.2017	[Apr.2016, Sep.2018]

Table S3. Key molecular markers of the six H5N1 viruses in the study.

Protein	Amino acid/ motif	G1	G2	Phenotypic consequences
HA[a]	Cleavage site	PLREKRRKR/G	PLREKRRKR/G	Feature of HPAIV
	S123P	P	P	Increased virus binding to the human receptor (α-2, 6)
	T160A	A	A	
	D94N	N	A	
PB2	L89V	V	V	Increase polymerase activity in mammals
	G309D	D	D	
	T339K	K	L	
	R477G	G	G	
PB1	S622G	G	G	Increase polymerase activity and virulence in mammals
PA	S515T	T	T	Increase polymerase activity in mammals
M1	N30D	D	D	Increase virulence in mammals
	I43M	M	M	
	T215A	A	A	
NS1	P42S	S	S	Increase virulence in mammals
	L103F	F	F	
	I106M	M	M	
	C138F	F	F	Decreased interferon response

[a] The numbering of HA is relative to A/New York/392/2004(H3N2).
